# Characterizing cyanopeptides and transformation products in freshwater: integrating targeted, suspect, and non-targeted analysis with in silico modeling

**DOI:** 10.1007/s00216-025-05999-6

**Published:** 2025-07-12

**Authors:** Audrey Roy-Lachapelle, Morgan Solliec, Christian Gagnon

**Affiliations:** 1 Environment and Climate Change Canada, Montréal, QC Canada; 2 Ministère de l’Environnement de la Lutte contre les changements climatiques de la Faune et des Parcs, Laval, QC Canada

**Keywords:** Cyanobacteria, Cyanopeptides, Cyanotoxins, Transformation products, Non-targeted analysis, Suspect screening

## Abstract

Harmful algal blooms (HABs) pose significant risks to environmental and public health, primarily through cyanotoxin production. Influenced by anthropogenic and climatic factors, cyanobacteria require advanced methods for identifying and characterizing their secondary metabolites. This study presents a multi-step approach to investigate the most abundant cyanopeptides in freshwater samples from agricultural and urban areas, aiming to improve their characterization and understand their environmental fate. A targeted method was developed to quantify 28 cyanopeptides across seven families, being one of the most extensive quantitative analyses of cyanopeptides. Significant concentrations of 14 congeners were detected, ranging from 0.038 to 5.68 µg L^−1^. A suspect screening method was developed and applied to expand detection, integrating CyanoMetDB and in silico modeling for the prediction of molecular features, increasing confidence in characterization. This approach enabled the identification of 26 uncommon cyanopeptides, including the newly characterized [DMAdda^5^, GluOMe^6^]microcystin-LHty. Additionally, a novel non-targeted analysis method was developed, combining compound class search, in silico modeling, and the enviPath UG & Co KG biotransformation prediction tool. This new strategy led to the identification of seven new transformation products and potential microcystins, including a new dopamine-modified microcystin-YR and the new linear [seco-1/7][Asp^3^]microcystin-LR. By integrating targeted, suspect, and non-targeted approaches, this study significantly enhanced cyanopeptide detection and characterization, providing valuable insights for environmental monitoring and public health protection.

**Graphical Abstract:**

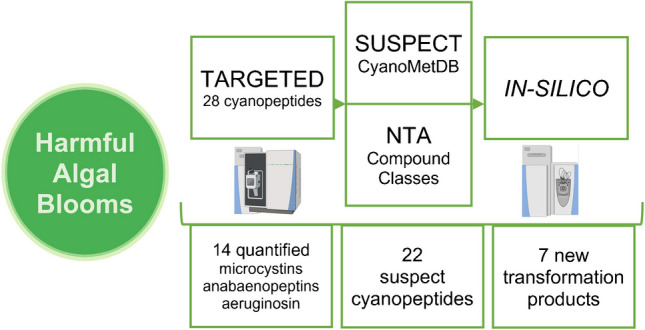

**Supplementary Information:**

The online version contains supplementary material available at 10.1007/s00216-025-05999-6.

## Introduction

Cyanobacteria play a major role in aquatic ecosystems, contributing to primary production and nutrient cycling [1]. However, the increasing frequency and intensity of harmful algal blooms (HABs) have raised concerns about the presence of cyanotoxins released in various water bodies. Cyanotoxins, produced by specific species of cyanobacteria, pose a significant threat to both aquatic ecosystems and public health [2]. Their increasing occurrence is influenced by anthropogenic factors such as nutrient inputs from agricultural and urban activities, as well as climate change, making them indirect contaminants of concern [3]. As the landscape of cyanobacterial secondary metabolites (CSMs) evolves, the identification and characterization of less-known and unknown cyanotoxins become essential for conducting thorough risk assessments, comprehensive studies, and environmental monitoring [2, 4].

CSMs comprise several distinct families with different modes of action. Among them, a diverse array of peptide families known as cyanopeptides include some of the greatest diversity of CSMs produced by cyanobacteria, with hundreds of reported congeners [5]. The most well-known are microcystins, a widely studied and prevalent family of cyclic peptides that inhibit protein phosphatases, leading to hepatotoxicity upon ingestion [6]. Beyond this well-characterized group, multiple families of cyanopeptides can be abundant in HABs, exhibiting bioactivities often related to microcystins, though not all are well characterized [7]. They are known to inhibit various types of enzymes, leading to disruptions in cellular functions, potential health risks, and ecological impacts. Among these families, nodularins, linear pentapeptides, act as hepatotoxins by inhibition of protein phosphatases like microcystins. Anabaenopeptins, a family increasingly recognized as prevalent in HABs, are recognized for their inhibition of carboxypeptidases A + B, serine protease, and serine/threonine phosphatases PP1 and PP2 [8, 9]. Cyanopeptolins demonstrate strong inhibition of chymotrypsin and elastase. Aeruginosins are characterized by their ability to inhibit various types of serine proteases. Microginins exhibit both protease inhibition and antimicrobial properties [10, 11]. Additionally, cyclic peptides like aerucyclamides add to the diversity of cyanobacterial peptides by demonstrating a range of bioactivities similar to those of microcystins [8].

The limited availability of certified standards poses a significant challenge in reporting cyanotoxins within the framework of public health recommendations, given their diversity including more than 300 congeners of microcystins [5, 12]. For example, only 15 certified standards for microcystins are commercially available, and there are only a few dozen certified standards and bioreagent products for some families of cyanotoxins and cyanopeptides. In addition, the variability of cyanotoxin groups and congeners produced by HABs can vary widely in a spatiotemporal manner [13]. Indeed, the influence of anthropogenic, environmental, and climatic factors leads to variations in the cyanobacterial species within HABs and consequently causes variations in the CSMs, including multiple compound families and numerous congeners. In this context, further investigation using suspect screening and non-targeted analysis (NTA) strategies is necessary to identify both known and unknown cyanotoxins and their transformation products, to better track the evolution of cyanobacterial blooms. This comprehensive approach enables a deeper understanding of the persistence of cyanotoxins, their fate and transformation within aquatic systems, and their specific effects on both the environment and public health.

Various analytical methods have been developed for the quantification of several cyanotoxin families and other cyanopeptides-related CSMs. These methods typically utilize liquid chromatography coupled with tandem mass spectrometry (LC–MS/MS) and high-resolution mass spectrometry (HRMS), aiming to broaden the scope of environmental monitoring for cyanotoxins. Many have included anatoxin and cylindrospermopsin in a list of microcystins, nodularin-R, and anabaenopeptins [14–19]. Additionally, several methods have included different families of CSMs such as cyanopeptolins, aeruginosins, microginins, and aerucyclamides with a maximum of 25 compounds analyzed simultaneously with new commercially available standards and in-house isolated cyanopeptides [20, 21].

Recent advancements in cost-effective, user-friendly HRMS systems have improved sensitivity, resolution, and analysis speed. These developments have enabled breakthroughs in environmental contaminant investigations, including cyanotoxins and CSMs identification, even in the absence of certified standards, although challenges remain for their validation. In addition, the integration of advanced data processing tools, databases, open-access platforms, and predictive algorithms enhances identification capabilities [22]. The identification of new cyanotoxins and CSMs has improved with resources like CyanoMetDB, an open-access database listing over 2600 cyanotoxins and CSMs facilitating suspect screening by UHPLC-HRMS [5, 21, 23–26]. Databases such as GNPS and mzCloud™ further aid suspect screening in water studies, although they are limited by available spectra [21, 25, 27]. NTA-based methods, like spectral searches of cyanopeptide fragments, have helped identify both known and unknown compounds using modern HRMS [17, 25, 28, 29]. Additionally, in-house Python coding has automated spectral searches for novel microcystins [30]. In addition, molecular networking via GNPS has enabled the identification of numerous cyanopeptides by clustering mass spectra based on structural similarity [31–33]. In recent developments, in silico modeling, integrated with CyanoMetDB and mzCloud™, has enhanced confidence in cyanopeptide identification by comparing predicted and experimental spectra [34]. The in silico experiment employs predictive modeling of fragmentation spectra based on molecular structure, followed by comparison with the experimental spectrum to improve the precision in compound identification. However, non-standardized methods for NTA remain complex and require manual confirmation from expert judgment, highlighting the need for more efficient characterization of diverse cyanotoxins, including unknowns and transformed products.

In this study, we propose a multi-step investigation of cyanopeptides in freshwater samples to improve their characterization and study their environmental fate. A comprehensive analysis of complex CSM mixtures was conducted, covering both intra- and extracellular fractions at low concentrations in surface water samples from agricultural and urban-impacted water bodies. A targeted method for the multi-class quantification of 28 cyanopeptides including microcystins, a nodularin, anabaenopeptins, aeruginosins, microginins, a cyanopeptolin, and an aeruginoguanidine was developed and enabled one of the most extensive quantitative analyses for cyanopeptides to date during episodes of HABs. A suspect screening method based on data-dependent acquisition was developed and applied to expand cyanopeptide detection in the samples, using CyanoMetDB within the Compound Discoverer 3.3 software. This approach involved applying an in silico methodology to increase confidence in proposed compounds. By predicting molecular features, in silico methods facilitate the identification of unknowns and support the investigation of potential cyanopeptides. Additionally, a new NTA approach combining compound class search and in silico modeling was developed, allowing for the identification and categorization of novel cyanopeptides and investigation of transformation products based on their structure similarities through fragmentation. These features help the categorization of detected features within specific compound groups of analogs, facilitating the data treatment and efficiently identifying unknown cyanopeptides by adding predictive structural characterization. This strategy was also supported by the use of enviPath model for biotransformation prediction, enabling the identification of potential transformation products and their metabolic pathways. This NTA method aimed to complement both targeted and suspect screening methods by expanding the scope of compound detection, allowing for in-depth analysis of unknowns and transformation products that would have otherwise remained undetected. This study demonstrates the potential of NTA-HRMS for identifying a broader range of cyanopeptides and other CSMs, paving the way for improved environmental monitoring and risk assessment.

## Materials and methods

### Chemicals, reagents, and stock solutions

Microcystins (MC-LR, [Asp^3^]-LR, [Asp^3^]-RR, -RR, -YR, -LA, -WR, -LW, -LF, -LY, -HtyR and -HilR), and Anabaenopeptins (AP-A, -B), Microginin 527 methyl ester (MG-527-ME), MG-690-ME, and Nodularin-R (NOD-R) (purity ≥ 95%) were purchased from Enzo Life Science (Farmingdale, NY, USA). AP-C, AP-J, AP-915, Ferintoic Acid A (FA-A), Oscillamide Y (OC-Y), MG-FR1, MG-FR2, Aeruginosins (AG-98A, −98B), and Aeruginoguanidine 98 A (AGU-98A) (purity ≥ 90%) as bioreagents were purchased from Cyano Biotech GmbH (Berlin, Germany). Cyanopeptolin 1041 (CP-1041) (purity ≥ 95%) was purchased from LTK Laboratories, Inc. (St. Paul, MN, USA). The following internal standards (IS): ^15^N_10_-MC-LR (97%), ^15^N_10_-MC-RR (98%), ^15^N_10_-MC-YR (98%), and ^15^N_10_-MC-LA (97%) were purchased from Cambridge Isotopes Laboratories, Inc. (Tewksbury, MA, USA). 2-Mercaptoethanol for HPLC derivatization (purity ≥ 99%) was purchased from MilliporeSigma Canada Ltd (Oakville, ON, Canada).

Individual stock solutions were made by dilution of 100 µg of solid standards in 1 mL of 80% methanol (MeOH) in water and stored at − 20 °C for a maximum of 12 months. The integrity of stock solutions was checked every month by injecting diluted solutions of each standard to prevail degradation and methylation of the standards. The intensity of standards in diluted solution was monitored, and variability was controlled to ensure it did not exceed 20%. Primary working solutions were prepared at a concentration of 1000 µg L^−1^ for targeted cyanotoxins and internal standards (IS) by dilution with water of individual stock solution aliquots. Subsequent working solutions were prepared daily by dilution in water to give solutions of desired concentration (1 to 250 µg L^−1^) and a final MeOH content of 10%. Primary working solutions and subsequent working solutions were kept at − 20 °C for a maximum of 3 months. All organic solvents and water used for dilutions were of LC–MS purity grade from Fisher Scientific (Whitby, ON, Canada).

### Sample collection, filtration, and extraction

The collected samples originated from the southern region of the province of Québec in the watershed of Lac Saint-Pierre and near agricultural runoff sources and urban centers. These sampling points under agriculture influence were chosen for their potential to undergo harmful algal blooms (HABs). Ten samples were collected in duplicates from Lac Saint-Pierre and the Yamaska River, as shown in Fig. S1 of the Supplementary Information. Furthermore, a sample from Lac Fortune, a lake known for its cyanobacteria blooms (Sample 11), and a lake sample from southern Quebec (Sample 12) were included to diversify the sampling and more effectively validate the methods developed in this study. At each sampling location, a duplicate set of samples was collected in 250 mL polyethylene terephthalate glycol-modified (PETG) bottles (Thermo Fisher Scientific™ Nalgene™, Waltham, MA, USA), previously conditioned with deionized water and MeOH, and rinsed three times with the surface water from the site [1]. The bottles were then filled to the brim, sealed, kept on ice in a cooler during the field trip and stored at − 20 °C. Prior to extraction, the samples underwent a cell lysis process involving three freeze-thawing cycles to effectively release the cyanotoxins. In this manner, the total fraction was analyzed to address potentially released concentrations in the rivers, ensuring a thorough assessment of the samples. The samples were subsequently filtered through 0.45 μm pore size hydrophilic CLARIFY-PTFE 25 mm syringe filters (Phenomenex, Torrance, CA, USA). The filters employed were carefully chosen to mitigate the potential adsorption of targeted compounds onto materials, as CLARIFY-PTFE filters offer low nonspecific binding and broad chemical compatibility.

The filtered samples were subjected to a solid-phase extraction (SPE) step for both cleanup and preconcentration using a 20-position manifold, and the SPE procedure was adapted from previous studies [35]. Samples were extracted using 50 mL aliquots spiked with 25 µL of 1 ppm ISs on HLB OASIS cartridges with a 6 mL volume and 200 mg of sorbent (Waters, Millford, MA, USA). The conditioning step comprised involving the cartridge with 5 mL of MeOH, followed by an additional 5 mL of deionized water. Sample loading onto the cartridge columns was done by gravity using 25 mL PTFE SPE reservoirs. Cartridges were then washed with 5 mL of deionized water containing 20% MeOH (v/v). Elution was carried out with 5 mL of MeOH into conical-bottom 15 mL glass tubes. Eluates were dried completely under a stream of nitrogen at 10 psi and kept at 55 °C for 130 min using RapidVap® Vertex™ Evaporator (Labonco Corporation, Kansas City, MO, USA). The extracts were reconstituted to a total volume of 500 µL of 10% MeOH in LC/MS grade water to maintain the same proportion of MeOH as at the start of the calibration separation while achieving a final ISs concentration of 50 µg L^−1^. Each SPE batch included a blank and a sample pool spiked with standards (MCs, APs, MGs, AGs, AGU-98A and CP-1041) at a final concentration of 25 µg L^−1^ for recovery correction and quantitative quality control. The recovery values are generally better than 75% except for CP-1041, and matrix suppression and enhancement are less than 15%; values are presented in Table S1. The extracted samples were transferred into 2 mL glass vials and stored at − 20 °C until analysis within 7 days.

### Instrumental conditions for target analysis

For quantification purposes, the samples were injected into the Vanquish Horizon Binary UHPLC system coupled to the TSQ Altis™ Plus mass spectrometer (Thermo Fisher Scientific, Waltham, MA, USA) with the triple quadrupole configuration used for better sensitivity. The UHPLC system was operated using Chromeleon 7.3 Software (Thermo Fisher Scientific, Waltham, MA, USA). The chromatographic separation of a 10 µL injected sample was done using a Hypersil Gold™ (150 × 2.1 mm, 1.9 µm particle size) column maintained at 40 °C, utilizing eluents A: water, and B: acetonitrile (ACN) with the addition of 0.1% formic acid at a flow rate of 400 μL min^−1^. The chromatographic gradient conditions are detailed in Fig. S2, with a total run duration of 15 min. Compounds detection was carried out using the selected reaction monitoring (SRM) mode with positive ionization and control was executed using TraceFinder 5.1 software (Thermo Fisher Scientific, Waltham, MA, USA). All details regarding ionization and detection parameters for quantitative analysis are presented in Table S2 and Table S3.

### Instrumental conditions for suspect screening and NTA

The suspect screening and NTA of samples were performed using Vanquish Horizon Binary UHPLC system coupled to the Q-Exactive Plus mass spectrometer (Thermo Fisher Scientific, Waltham, MA). The UHPLC system was operated using Chromeleon 7.3 Software (Thermo Fisher Scientific, Waltham, MA, USA). The chromatographic separation of a 10 µL injected sample was done using a Hypersil Gold™ (150 × 2.1 mm, 3 µm particle size) column maintained at 30 °C, employing water (A) and ACN (B) with the addition of 0.1% formic acid at a flow rate of 200 μL min^−1^. The chromatographic gradient conditions are detailed in Fig. S2, with a total duration of 30 min. A longer chromatographic gradient and larger particle size column were used to ensure a minimum of 7 acquisition points per peak, considering the dual scan time of the mass spectrometer, which is required for reliable peak characterization in non-targeted analysis [22].

Compounds detection was carried out using the data-dependent acquisition mode (DDA) with positive ionization, and control was executed using Xcalibur 4.4 software (Thermo Fisher Scientific, Waltham, MA, USA). Each DDA cycle included one full scan (FS) event with a resolving power set at 70,000 at full width at half maximum (FWHM) at *m/z* 200, scanning from *m/z* 300 to 1400. Upon reaching a threshold of 1.5E5, a top 5 mass isolation was triggered for fragmentation, with detection performed at a resolving power set at 17,500 FWHM (*m/z* 200). Normalized collision energies (NCE) were stepped at 15, 30, and 45 to guarantee optimal fragmentation of ions. All details regarding ionization and detection parameters for the DDA analyses are presented in Table S2.

Instrument calibration in positive mode was performed every 7 days through direct infusion of an LTQ Velos ESI Positive Ion Calibration Solution (Pierce Biotechnology Inc., Rockford, IL). Throughout the 7 days post-calibration, mass accuracy for all target compounds was maintained within the 5-ppm range.

### Target analysis validation and quantification

In all optimization experiments, analytes were spiked into a water matrix consisting of analyte-free surface water sampled before the HABs season in early spring. A prescreen was done with a previous method that confirmed the absence of cyanopeptides in the samples [16]. Five replicates were spiked at low-level concentration from the linearity range (25 µg L^−1^). Data treatment was done using the TraceFinder 5.1 software. A six-point calibration curve was used for quantification purposes, covering the following levels (1, 10, 25, 50, 100 and 250 µg L^−1^) in accordance with the method quantification limits (MQL) and a quality control (QC) of 25 µg L^−1^ was employed for signal recovery correction and accuracy verification. The ISs were added at mid-level from the calibration curve with 50 µg L^−1^. Samples were subjected to a quantitative analysis to monitor 28 known cyanotoxins (microcystins: -[Asp^3^]LR, -[Asp^3^]RR, -LR, -RR, -YR, -LA, -LY, -LW, -LF, -WR, -HtyR and –HilR; anabaenopeptins: -A, -B, -C, -J, −915, ferintoic acid A, oscillamide Y; microginins: -FR1, -FR2, −527-ME, −690-ME; aeruginosin 98 A and −98B; aeruginoguanidine 98 A; cyanopeptolin 1041 and nodularin-R). The method detection and quantification limits (MDL and MQL) are presented in Table S1, Supplementary Information, and reached low ng L^−1^ levels, enabling detection at low environmental concentrations for cyanopeptides. Accuracy and precision are presented in Table S4, with two levels of concentration (2.5 and 50 µg L^−1^), and are satisfactory with a bias of less than 15%. Samples with notable and interesting results, such as elevated concentrations of cyanotoxins and the presence of less common cyanopeptides like anabaenopeptins, were chosen for additional suspect screening and NTA seeking unknown congeners and potential transformation products (TPs).

### Suspect screening data processing

The DDA data were processed using Compound Discoverer 3.3 (CD3.3—Thermo Fisher Scientific, Waltham, MA, USA). The CyanoMetDB 2023 database was imported into a mass list in CD3.3, and the compound structures were manually added by converting the canonical SMILES as mol files. Adding the structures enabled Fragment Ion Search (FISh) scoring, a software option that performs in silico fragmentation using the Mass Frontier™ Spectral Interpretation Software integrated in CD3.3.

The workflow was built for searching for suspect compounds using database searches, in this instance CyanoMetDB 2023. The data processing parameters from the workflow nodes are detailed in Table S5. Different filters are subsequently applied after data processing for feature selection to narrow down the list of masses detected in DDA and facilitate structural interpretation. These filters include:

- Mass list matching: Ensures consistency with the database,
- MS^2^ detection: Confirms the presence of MS^2^ spectra,
- Peak rating: Retains chromatograms with a rating > 4.00, indicating satisfactory quality,
- Isotopic pattern verification: Checks isotopic patterns match with predicted composition,
- Retention time comparison: Eliminates features with significant retention time deviations based on polarity.

The remaining features are then subjected to FISh scoring, followed by manual analysis of fragmentation spectra. To ensure quality control, three blank types (travel, sample preparation, and instrument) with distilled water were monitored for potential contamination. Samples were pooled and spiked with the 28 cyanopeptide standards at 1 µg L^−1^ and 25 µg L^−1^, and with internal standards (IS) at 50 µg L^−1^. These controls underwent all stages of extraction, analysis, and interpretation to ensure method robustness. All targeted cyanopeptides were detected, giving acceptable in silico coverage (> 50%) and high compound class coverage (> 85%), where the percentage of coverage (%) represents the proportion of predicted molecular fragments matching the observed spectra.

### NTA data processing

A new NTA method was developed to identify new congeners and TPs without relying on existing databases. For this purpose, a compound class list for each compound was generated, incorporating the fragment structures proposed by FISh scoring. To achieve this, standards used for quantitative analysis were injected at a concentration of 250 µg L^−1^, using the DDA method employed for the samples. Results were processed with the same workflow in CD3.3. The identified compounds were subjected to in silico FISh experiment to elucidate the structure of fragments in the MS^2^ spectra. Supplementary Information 2 includes compound classes with their FISh-generated fragments, exact masses (*m/z*), molecular formulas, and charge state. A new workflow was built for compound searches based on these compound classes, with data processing parameters outlined in Table S5. Following data processing, the same filters used for suspect screening are subsequently applied. These filters include selecting compound classes with in silico coverage of 30% or higher. The selected features underwent subsequent manual analysis of fragmentation for new cyanopeptides or TPs.

### In silico fragmentation and spectra interpretation

The compiled putative cyanopeptides and TPs were compared with their retention times and calculated partition coefficients (Log P) values, as predicted by the Molinspiration Cheminformatics predictor tool (Bratislava University, Slovakia); see Table S6 for Log P of targeted compounds. Molinspiration offers a free physico-chemical properties calculator providing reliable predictions with well-established and validated computational methods [36]. This comparison ensured plausible chromatographic separation based on structural similarities.

The detected features were then subjected to in silico fragmentation using FISh scoring based on the predicted structures. These experiments employed a 5-ppm mass tolerance and a signal-to-noise ratio threshold of 5. Features passing the in silico coverage threshold of 30% were subjected to manual interpretation in two steps:

- Search for characteristic fragments: Initial confirmation involved identifying characteristic fragments of well-known cyanopeptide families to confirm the status of the compound as a congener [25, 29, 37–41],
- Detailed spectral assessment determines the amino acid sequence and the potential identity of the congener. This interpretation was also demonstrated in a prior study [29].

The minimum number of fragments required for the confident identification of a cyanopeptide depends on its molecular structure. Since the position of each amino acid must be resolved in the mass spectra, fragment ions corresponding to individual amino acids or peptide sequences are essential for unambiguous structural characterization and differentiation of isomers [29]. To assess the accuracy of spectral matches, we applied the Schymanski classification system, which evaluates the confidence of spectral identifications based on the extent of fragmentation coverage, using a ranking system from Level 1 to Level 5. Level 1 indicates high-confidence identification based on complete and thorough fragmentation data (e.g., full sequence coverage, often confirmed by comparison to standards or reference databases), while Level 5 was presenting identification primarily based on the exact mass of the parent ion alone, with minimal or no fragmentation data available [42].

To facilitate TP searches, potential cyanopeptide TPs were predicted using enviPath UG & Co KG, an open-access tool for modeling biotransformation pathways of environmental contaminants [43, 44]. EnviPath was chosen for its unique combination of open-access predictive algorithms, expert-curated transformation pathways, and the ability to integrate user-generated data, making it more versatile and adaptable for environmental degradation studies. Additionally, its user-friendly interface enables more efficient use, allowing for faster data processing.

### Derivatization with mercaptoethanol

The use of the derivatization of cyanobacterial samples by mercaptoethanol was previously developed to differentiate microcystins that contain the isobaric amino acids* N*-methyldehydro-alanine (Mdha) and dehydrobutyrine (Dhb) by LC–MS analysis [45]. It selectively reduces Mdha to thiazolidine while leaving Dhb unaffected, enabling their distinct identification based on their chemical behavior during derivatization. This process tags reactive microcystins with an additional mass of 78 Da, helping to confirm the presence of microcystins with Mdha residues. Derivatization was performed on microcystin-positive samples confirmed by target analysis, following the procedure outlined by Miles et al. [45].

## Results and discussion

### Quantification of cyanopeptides

A quantitative screening was initially conducted for 28 cyanopeptides across seven families to detect the most common compounds produced by planktonic and benthic cyanobacterial blooms in various water sources across Québec, Canada. Positive samples then underwent further suspect screening and NTA, focusing on samples more likely to contain cyanobacterial secondary metabolites (CSMs). The sampling locations showed distinct cyanopeptide profiles (Tables 1 and 2) and varying dilution factors, which reflect the hydrological characteristics of small water bodies, large lakes, and rivers.

**Table 1:** Quantification results for microcystins (MCs) (µg L^−1^)

Site No	Dates	MC-RR	MC-YR	MC-HtyR	[Asp^3^]-MC-LR	MC-LR	MC-HilR	MC-LA	MC-LY
1	2019–08-27	< LOD	< LOD	< LOD	< LOD	0.169 ± 0.003	< LOD	< LOD	< LOD
2	2019–08-27	< LOD	0.36 ± 0.01	0.446 ± 0.007	0.552 ± 0.005	0.278 ± 0.004	< LOD	< LOD	< LOD
3	2019–08-27	< LOD	0.941 ± 0.006	< LOD	0.178 ± 0.004	0.152 ± 0.004	< LOD	< LOD	< LOD
4	2019–08-27	< LOD	< LOD	< LOD	< LOD	0.038 ± 0.002	< LOD	< LOD	< LOD
5	2019–08-27	< LOD	< LOD	< LOD	< LOD	0.085 ± 0.002	< LOD	< LOD	< LOD
6	2022–07-20	< LOD	< LOD	< LOD	< LOD	< LOD	< LOD	< LOD	< LOD
7	2022–07-20	< LOD	< LOD	< LOD	< LOD	< LOD	< LOD	< LOD	< LOD
8	2022–07-20	< LOD	< LOD	< LOD	< LOD	< LOD	< LOD	< LOD	< LOD
9	2023–08-21	< LOD	< LOD	< LOD	< LOD	0.23 ± 0.03	< LOD	< LOD	< LOD
10	2023–09-06	< LOD	< LOD	< LOD	< LOD	< LOD	< LOD	< LOD	< LOD
11	2021–09-13	< LOD	4.25 ± 0.05	0.083 ± 0.001	2.08 ± 0.03	< LOD	< LOD	< LOD	< LOD
12	2021–08-27	0.038 ± 0.001	< LOD	0.126 ± 0.005	0.028 ± 0.001	1.91 ± 0.06	0.022 ± 0.001	0.084 ± 0.002	0.010 ± 0.001

**Table 2:** Quantification results for aeruginosin 98 A (AG-98A) and anabaenopeptins (APs) (µg L^−1^)

Site No	Dates	AG-98A	AP-A	AP-B	AP-C	FA-A	OC-Y
1	2019–08-27	< LOD	< LOD	< LOD	< LOD	< LOD	< LOD
2	2019–08-27	< LOD	< LOD	< LOD	< LOD	< LOD	< LOD
3	2019–08-27	< LOD	< LOD	< LOD	< LOD	< LOD	< LOD
4	2019–08-27	< LOD	< LOD	< LOD	< LOD	< LOD	< LOD
5	2019–08-27	< LOD	< LOD	< LOD	< LOD	< LOD	< LOD
6	2022–07-20	< LOD	0.78 ± 0.02	0.258 ± 0.002	< LOD	< LOD	0.399 ± 0.005
7	2022–07-20	< LOD	1.03 ± 0.04	0.332 ± 0.002	< LOD	< LOD	0.52 ± 0.02
8	2022–07-20	< LOD	0.79 ± 0.01	0.314 ± 0.004	< LOD	< LOD	0.44 ± 0.01
9	2023–08-21	< LOD	< LOD	< LOD	< LOD	< LOD	< LOD
10	2023–09-06	< LOD	0.154 ± 0.002	< LOD	< LOD	< LOD	0.39 ± 0.05
11	2021–09-13	< LOD	5.68 ± 0.04	2.93 ± 0.01	2.04 ± 0.08	0.019 ± 0.008	0.88 ± 0.01
12	2021–08-27	0.035 ± 0.001	< LOD	< LOD	< LOD	< LOD	< LOD

In Lake Saint-Pierre (Sites 1 to 5), four microcystin congeners (microcystin-YR, microcystin-HtyR, [Asp^3^]microcystin-LR, and microcystin-LR) were detected at concentrations approaching 1 µg L^−1^. This marks the first detection of significant microcystin concentrations in a lake historically dominated by *Lyngbya wollei* proliferations [46]*.* In the Yamaska River (Sites 6 to 10), a tributary of Lake Saint-Pierre, anabaenopeptins (anabaenopeptin A, anabaenopeptin B, and oscillamide Y) were detected at concentrations up to 1 µg L^−1^. This is the first report of anabaenopeptins in the Yamaska watershed at levels near World Health Organization's guidelines for microcystins in potable water, indicating a significant cyanobacterial presence. Notably, Site 9, near the mouth of Lake Saint-Pierre, exhibited only microcystin-LR, suggesting a mix of water from both the lake and river.

The Yamaska River showed a dominance of anabaenopeptins during cyanobacterial blooms, while Lake Saint-Pierre primarily featured microcystins. Although cyanobacteria species were not identified, the varying cyanopeptide profiles suggest environmental conditions are driving different cyanobacteria or CSM production [16, 17, 23, 47]. Sample 11 revealed a greater diversity of cyanopeptides, with five anabaenopeptins (-A, -B, -C, FA-A, and OC-Y) and three microcystins (-YR, -HtyR, and -[Asp^3^]LR) ranging from 0.019 to 5.68 µg L^−1^. This finding highlights the presence of less common cyanopeptide families, some of which are newly detected in some locations within the lake.

At site 12, primarily featured microcystins, seven congeners (-RR, -YR, -HtyR, -[Asp^3^]LR, -LR, -HilR, -LA, and -LY) were detected at concentrations ranging from 0.01 to 1.91 µg L^−1^. Additionally, a rare aeruginosin 98 A congener was detected at 0.035 µg L^−1^—its first report in Québec. These findings highlight the diversity of cyanotoxin profiles, with newly reported cyanopeptides in the area, influenced by local environmental conditions and cyanobacterial prevalence. The presence of a broad range of cyanopeptides, many at toxicologically significant levels when measured as microcystin equivalents, underscores the potential risks to human health and the environment. Often, these compounds go undetected, leaving gaps in understanding their full ecotoxicological and public health impacts.

### Suspect screening and in silico on CSMs from CyanoMetDB

A total of 26 cyanopeptides were detected across six sites, including 22 congeners: twelve microcystins, six anabaenopeptins, one microginin, one aeruginopeptin, one cyanopeptolin, and one aeruginosin. The structures are illustrated in Fig. S3. Table 3 provides detailed data on the identified cyanopeptides, including precursor ions (M + H^+^), theoretical Log P, retention times (confirming polarity), signal intensities (indicating relative abundance), and the in silico coverage showing the percentage of similarity (range?) between theoretical and experimental spectra. The following sections break down the identification and spectral interpretation for each potential cyanopeptide, grouped by family. Since this is a suspect screening, thorough characterization remains essential given the limited spectral information available. CyanoMetDB only provides exact precursor masses, and many compounds have been reported only once in the literature or lack available mass spectra.

**Table 3:** Cyanopeptides identification using in silico fragmentation through FISh scoring experiment

Compounds	M + H^+^	RT^1^ (min)	Log P	Signal intensity	In silico coverage %	Site no
[Ser7]Microcystin-LR	999.5503	10.22	− 4.76	1.89E + 06	50%	2
[DMAdda^5^, GluOMe^6^]Microcystin-LHty	1016.5310	16.24	1.5	1.51E + 06	35%	2
[seco-4/5][D-Asp^3^]Microcystin-HtyR*	1063.5370	9.68	− 4.63	1.62E + 07	41%	2
Microginin 690	691.3350	9.11	− 0.78	4.07E + 07	44%	3
Aeruginopeptin 228A	1045.4854	10.25	− 1.28	4.64E + 06	32%	3
Anabaenopeptin F	851.4758	9.10	− 0.97	4.35E + 08	66%	7
Anabaenopeptin E	851.4755	9.30	− 0.63	2.67E + 07	55%	7
Anabaenopeptin H	923.5311	9.29	0.48	1.18E + 07	44%	7
Anabaenopeptin HU892	893.5226	9.92	0.33	9.00E + 06	58%	10
Anabaenopeptin SA3	823.4698	8.67	− 0.39	1.75E + 07	34%	10
Anabaenopeptin 679*	680.3766	9.95	1.1	1.95E + 07	63%	10
[D-Asp^3^]Microcystin-MR	999.4937	10.92	− 3.62	5.06E + 06	43%	11
[D-Asp^3^]Microcystin-M(O)R	1015.4898	10.35	− 4.76	1.42E + 07	46%	11
[DMAdda^5^]Microcystin-YR	1031.5168	9.60	− 4.37	1.29E + 06	42%	11
[D-Ser^1^, D-Asp^3^]Microcystin-HtyR	1061.5292	10.83	− 3.54	1.49E + 07	31%	11
[D-Asp^3^, DMAdda^5^]Microcystin-LR	967.5246	9.77	− 3.38	6.30E + 06	45%	11
Anabaenopeptin F	851.4768	9.10	− 0.97	7.87E + 07	66%	11
Anabaenopeptin E	851.4768	9.30	− 0.63	1.95E + 07	58%	11
Anabaenopeptin SA3	823.4705	8.68	− 0.39	1.71E + 07	36%	11
Anabaenopeptin 679*	637.3700	9.96	1.1	3.39E + 06	70%	11
Cyanopeptolin 1081	1082.5393	9.97	− 2.49	6.68E + 06	36%	11
Aeruginosin A	617.3458	9.76	1.93	2.92E + 06	52%	11
[Mdha-GSH^7^]Microcystin-LR*	1302.6398	10.28	− 5.95	5.57E + 07	35%	12
[epoxyAdda^5^]Microcystin-LR*	1011.5501	11.25	− 2.84	5.41E + 07	37%	12
[DMAdda^5^]Microcystin-LR*	981.5108	9.83	− 4.22	6.43E + 07	35%	12
[seco-1/2]Microcystin-LR*	1013.5672	10.88	− 5.03	9.92E + 06	46%	12

^1^Retention time

^*^Potential transformation product or metabolite

#### Microcystins

Twelve distinct microcystin congeners were identified in samples 2, 11, and 12, all of which were already dominated by the presence of microcystins (Tables 1 and 3). Detailed fragment interpretation for each microcystin found by CyanoMetDB is presented in Table S7. Notably, the search for the key fragment ion *m/z* 135 assists the initial screening process of microcystins. This ion represents a characteristic fragment ion resulting from the cleavage of Adda moiety, a unique amino acid present in all microcystin variants [41].

**Fig. 1:**
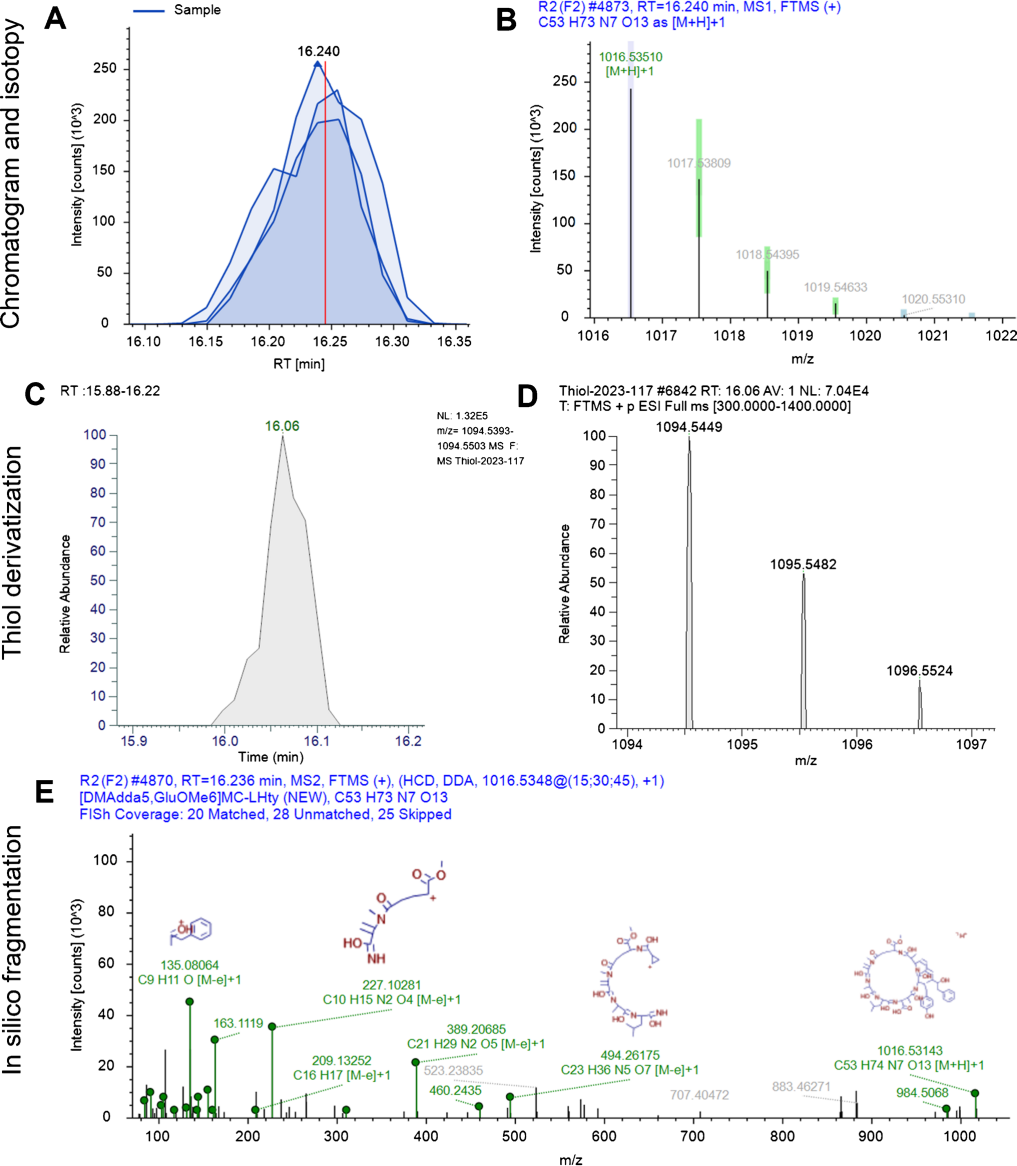
Structure characterization of new [DMAdda^5^, GluOMe^6^]microcystin-LHty. **A** Extracted ion chromatogram of ion *m/z* 1016.5314 and **B** isotopic pattern of most intense precursor ion. **C** Extracted ion chromatogram of thiol derivative ion *m/z* 1094.5599 and **D** isotopic pattern of thiol derivative. **E** Fragmentation spectrum and in silico matching with FISh coverage

In sample 2, three potential microcystins were identified. [Ser^7^]microcystin-LR was confirmed based on the spectra in Fig. S4, supported by the absence of a thiol group at position 7, which is replaced by a serine. This congener is close to [Asp^3^]microcystin-LR and microcystin-LR, which were also detected in the sample. [DMAdda^5^, GluOMe^6^]microcystin-LHty, a newly discovered congener, shares the same molecular formula as the CyanoMetDB-proposed microcystin-LHty but includes DMAdda at position 5 and GluOMe at position 6 as shown in Fig. 1 and detailed in SI-2. The presence of LHty in the structure is unusual and indicates a potentially greater structural diversity within cyanobacterial blooms, typically dominated by more common variants and their close analogs. Such diversity can affect how we assess the ecological and health risks associated with blooms, as different variants may have varying levels of toxicity, stability, or environmental persistence. Linear [seco-4/5][D-Asp^3^]microcystin-HtyR, with the structure Adda^5^-Glu^6^-Mdha^7^-Ala^1^-Hty^2^-Asp^3^-Arg^4^, was also identified, with [D-Asp^3^]microcystin-HtyR hydrolyzed between Arg^4^ and Adda^5^ (Fig. S5). Linear microcystin congeners are often non-toxic due to the importance of the cyclic structure for bioactivity, but more toxicological data is needed [48, 49].

In sample 11, five microcystins were proposed. [D-Asp^3^]microcystin-M(O)R, an oxidized form of [D-Asp^3^]microcystin-MR (Figs. S6 and S7), exhibited a signal three times stronger than the unoxidized version, likely due to degradation within environmental or laboratory conditions. Methionine-containing microcystins are rarely reported, and this is their first detection in Québec watersheds [50]. [DMAdda^5^]microcystin-YR was identified as a demethylated form of microcystin-YR at the Adda function, supported by its spectral similarity to microcystin-YR, which is the dominating congener of sample 11 (Fig. S8). The demethylation may result from incomplete methylation during biosynthesis or ADMAdda instability [41]. [D-Ser^1^, D-Asp^3^]microcystin-HtyR (Fig. S9) was proposed as a candidate closely related to the common microcystin-HtyR, a congener detected in this sample. The last microcystin detected in the sample, [D-Asp^3^, DMAdda^5^]microcystin-LR, is closely related to [Asp^3^]microcystin-LR, with spectra detailed in Fig. S10. The abundance of [Asp^3^]microcystin-LR in this sample suggests that incomplete methylation or ADMAdda artifacts from environmental transformation may explain the formation of this congener.

In sample 12, four uncommon microcystins were proposed. The [Mdha-GSH^7^]microcystin-LR variant was identified by the absence of a thiol derivative, as glutathione (GSH) bound to the *α,β*-unsaturated carbonyl of the Mdha moiety (Fig. S11) [51]. This GSH conjugate, part of the cellular detoxification pathway for microcystin-LR, retains toxicity [51]. [epoxyAdda^5^]microcystin-LR was found to be likely an oxidized variant of microcystin-LR generated via metabolism or autooxidation (Fig. S12) [41]. [DMAdda^5^]microcystin-LR was proposed with spectra (Fig. S13), and given the abundance of microcystin-LR, it may result from incomplete methylation during biosynthesis. Lastly, [seco-1/2] microcystin-LR (Fig. S14), a linear form of microcystin-LR hydrolyzed between positions 1 and 2, is a non-toxic biogenetic precursor [48, 49]. Sample 12 exemplifies microcystin diversity, with microcystin-LR predominating alongside a precursor, and metabolized, oxidized, and demethylated variants. Semi-quantitative calculations, relative to microcystin-LR and excluding [seco-1/2], yielded a total concentration of 0.32 µg L^−1^, which significantly contributes to the total concentration of 1.91 µg L^−1^ for microcystin-LR. Failing to monitor important TPs and microcystin-LR analogs with similar toxicity can have serious consequences for public health, as it may lead to the omission of drinking water and recreational water advisories, such as consumption bans or swimming restrictions.

The distinct profiles of microcystin congeners across these microcystin-rich samples (2, 11, and 12) reveal insights into the dynamics of cyanobacterial blooms. Sample 2 emphasizes uncommon variants, which may suggest localized environmental conditions favoring their production, while sample 11 showed the occurrence of oxidation and methylation modifications of different congeners, and sample 12 showcased metabolized and transformed forms of microcystin-LR. This suggests that focusing solely on the dominant congener may underestimate the total toxicity, as other variants and degradation products can also contribute to harmful effects.

#### Anabaenopeptins

Six different anabaenopeptin (AP) congeners were detected in samples 7, 10, and 11, where this cyanopeptide family was prevalent (Tables 2 and 3). A key feature in all identified anabaenopeptins is the presence of fragments at *m/z* 84 and 129, representing the immonium ion and residue of lysine, respectively. These fragments are essential markers for identifying AP and consistently appear in all intact anabaenopeptins (Fig. S3). A detailed analysis of AP fragments is provided in Table S8.

In samples 7 and 11, anabaenopeptin F and anabaenopeptin E were identified. Although they share the same molecular formula, they differ by a methyl shift between positions 3 and 4 (Ile^3^-Hty^4^ for anabaenopeptin F in Fig. S15 and SI-3 and Val^3^-MeHty^4^ for anabaenopeptin E in Fig. S16). The co-occurrence of anabaenopeptin F and anabaenopeptin E has been linked with the presence of anabaenopeptin B and oscillamide Y, which were also found in the samples, both common in freshwater blooms dominated by anabaenopeptins [17, 29, 47]. Additionally, anabaenopeptin H was detected in sample 7, with the structure Arg^1^-CO-Lys^2^-Ile^3^-Hty^4^-MeHty^5^-Ile^6^ (Fig. S17). This congener is not detected as frequently as its B and F counterparts, but its similar structure and association with *Oscillatoria* suggest that its presence is plausible [52].

In sample 10, three anabaenopeptins were identified. Anabaenopeptin HU892 features the structure Arg^1^-CO-Lys^2^-Val^3^-Hph^4^-MeHty^5^-Ile^6^ (Fig. S18). This congener belongs to the presence of an unusual subgroup, characterized by an aliphatic amino acid at the carboxyl end and an *N*-methyl-homoaromatic amino acid at position 2 [53]. Anabaenopeptin SA3 and anabaenopeptin 679 were found in samples 10 and 11, which show similar fragmentation patterns (Figs. S19 and S20). The proposed structure for anabaenopeptin SA3 is Lys^1^-CO-Lys^2^-Val^3^-Hty^4^-MeAla^5^-Phe^6^. Regarding anabaenopeptin 679, its biosynthesis remains unclear, but the absence of the Lys-CO side chain at position 1 compared to anabaenopeptin SA3 suggests a potential degradation pathway or a different biosynthesis route by cyanobacteria, possibly the *Planktothrix* species [54, 55]. Further research is needed to confirm their production mechanisms.

#### Other cyanopeptides

In addition to the dominant identified cyanopeptide families like microcystins and anabaenopeptins, four cyanopeptides rarely documented in the literature were tentatively identified: microginin 690 and aeruginopeptin 228 A in sample 3, and cyanopeptolin 1081 and aeruginosin A in sample 11. Detailed spectral interpretations are provided in Table S9.

Microginin 690 is a linear oligopeptide featuring a decanoic acid derivative (Adha) at its N-terminus, with the structure Adha^1^-Tyr^2^-MeMet(O)^3^-Tyr^4^ (Fig. S3, spectra in Fig. S21). Although rarely documented, Zervou et al. linked microginin 690 to *Microcystis aeruginosa*, making its presence in sample 3 highly probable [56]. Aeruginopeptin 228 A, also identified in sample 3, is a depsipeptide known for its cytotoxic properties. It has been associated with *Microcystis aeruginosa*, strengthening its identification in this sample [57]. Its structure, Hpla^1^-Gln^2^-Thr^3^-Tyr^4^-Ahp^5^-Thr^6^-MePhe^7^-Ile^8^, is detailed from spectra in Fig. S22. To our knowledge, this marks the first detection of an aeruginopeptin variant in a Canadian water source.

Cyanopeptolin 1081 is a cyclic depsipeptide with Thr at the first position, linked through an ester bond on a beta-hydroxyl group, and contains an Ahp residue within the cycle (Fig. S3). A few studies indicate that cyanopeptolins cause neurotoxic effects in model organisms, demonstrating a similar toxic impact to that of microcystins [58, 59]. Its structure, Tyr^1^-GA-Gln^2^-Thr^3^-Leu^4^-Ahp^5^-Thr^6^-NMe-Phe^7^-Ile^8^, was confirmed through spectra analysis (Fig. S23) and aligns with the findings of Bober et al., who recently identified this cyanopeptolin congener [60]. To our knowledge, this is a rare report of a cyanopeptolin variant in river systems.

Finally, aeruginosin A proved difficult to confirm, as it was detected repeatedly in all samples with four closely related hits between retention times 8.3 and 9.7 min, corresponding to ions *m/z* 617.3439, 617.3434, 617.3443, and 617.3443, with in silico scores ranging from 49 to 55%. The ion at 9.7 min was finally proposed as aeruginosin A against other signals (Fig. S24). Aeruginosin A is a linear tetrapeptide (Fig. S3) featuring the Choi moiety (2-carboxy-6-hydroxy-octahydroindole), a common component of all aeruginosin congeners. This moiety is linked to a hydrophobic amino acid, a 4-hydroxyphenyl lactate derivative at the N-terminus, and an arginine derivative at the C-terminus [61]. Aeruginosin A is recognized for its inhibitory effects on fVIIa and thrombin [37].

#### Level of confidence in identification for suspect screening

Tables 1 and S6 provide retention times and Log P values for known standards as well as tentatively identified compounds. While Log P is not automatically correlated with retention times due to structural properties influencing chromatographic behavior, it serves as a useful indicator to assess whether the retention time of tentatively identified compounds falls within a plausible range. For instance, [DMAdda^5^]microcystin-YR (Log P − 4.37) at 9.6 min can be compared with MC-LR (Log P − 3.64) at 11.17 min, where the more hydrophobic MC-LR shows a longer retention time. Similarly, AP-F (Log P − 0.97) at 9.1 min aligns with AP-B (Log P − 1.47) at 8.73 min, where the lower Log P of AP-B results in a shorter retention time. Such comparisons help increase confidence in identifications during the suspect screening process.

Additionally, according to the Schymanski classification system, cyanopeptides identified through suspect screening using CyanoMetDB are assigned to Level 2b because their identification relies on partial fragmentation coverage and comparisons with a database of known compounds and published mass spectra [42]. In suspect screening, the exact identity of the compound is not predetermined, and the identification is based on mass spectra, fragmentation patterns, and database matches rather than complete fragmentation data. As a result, the identification of cyanopeptides at this level of confidence remains tentative.

### NTA and in silico on compound classes scoring

A new NTA strategy was developed and employed, using a compound class coverage method, to search for uncommon cyanopeptide congeners and TPs. Seven TPs were identified, linked to eight microcystins and anabaenopeptins, as summarized in Table 4. This table lists the proposed TP’s chemical formula, precursor ion (M + H^+^), retention time, class coverage, in silico coverage, related degradation compounds, and the sample in which the TPs were detected. Additionally, the enviPath model was used to cross-check the identity of parent compounds and draw potential degradation pathways, providing additional confidence in the proposed transformations [44].

**Table 4:** Transformation products (TPs) identification using non-targeted analysis

ID^1^	Chemical formula	M + H^+^	RT^2^ (min)	Class coverage %	In silico coverage %	Related compounds^3^	Sample
TP1	C34H48N6O6	637.3703	8.94	43.8	62.5	AP-B, AP-C	11
TP2*	C35H49N7O7	680.3755	9.95	43.8	46.5	AP-B, AP-C, AP-E, AP-SA3	10, 11
TP3	C36H50N6O8	695.3759	10.44	40.0	40.9	OC-Y	11
TP4	C36H50N6O9	711.3710	7.61	40.0	37.7	OC-Y	7, 11
TP5	C46H74N10O9	911.5712	10.93	58.7	42.6	[Asp^3^]MC-LR	2, 11
TP6*	C48H74N10O13	999.5491	9.87	70.8	39.5	[Asp^3^]MC-LR	11
TP7*	C52H72N10O14	1061.5301	10.42	37.3	36.0	MC-YR	3, 11

^1^Transformation products identification

^2^Retention time

^3^Acronyms definition: *AP-B*, anabaenopeptin B; *AP-C*, anabaenopeptin C; *AP-E*, anabaenopeptin E; *AP-SA3*, anabaenopeptin SA3; *OC-Y*, oscillamide Y; *[Asp*^*3*^*]MC-LR*, [Asp^3^]microcystin-LR; *MC-YR*, microcystin-YR

^*^Potential transformation product or metabolite

#### Transformation products (TPs) elucidation

**Fig. 2:**
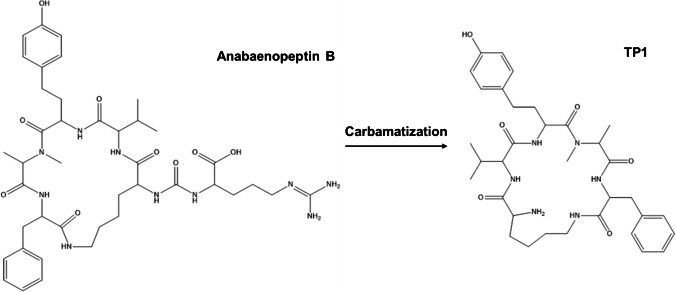
Proposed transformation product 1 (TP1) formation by carbamatization of anabaenopeptin B

**Fig. 3:**
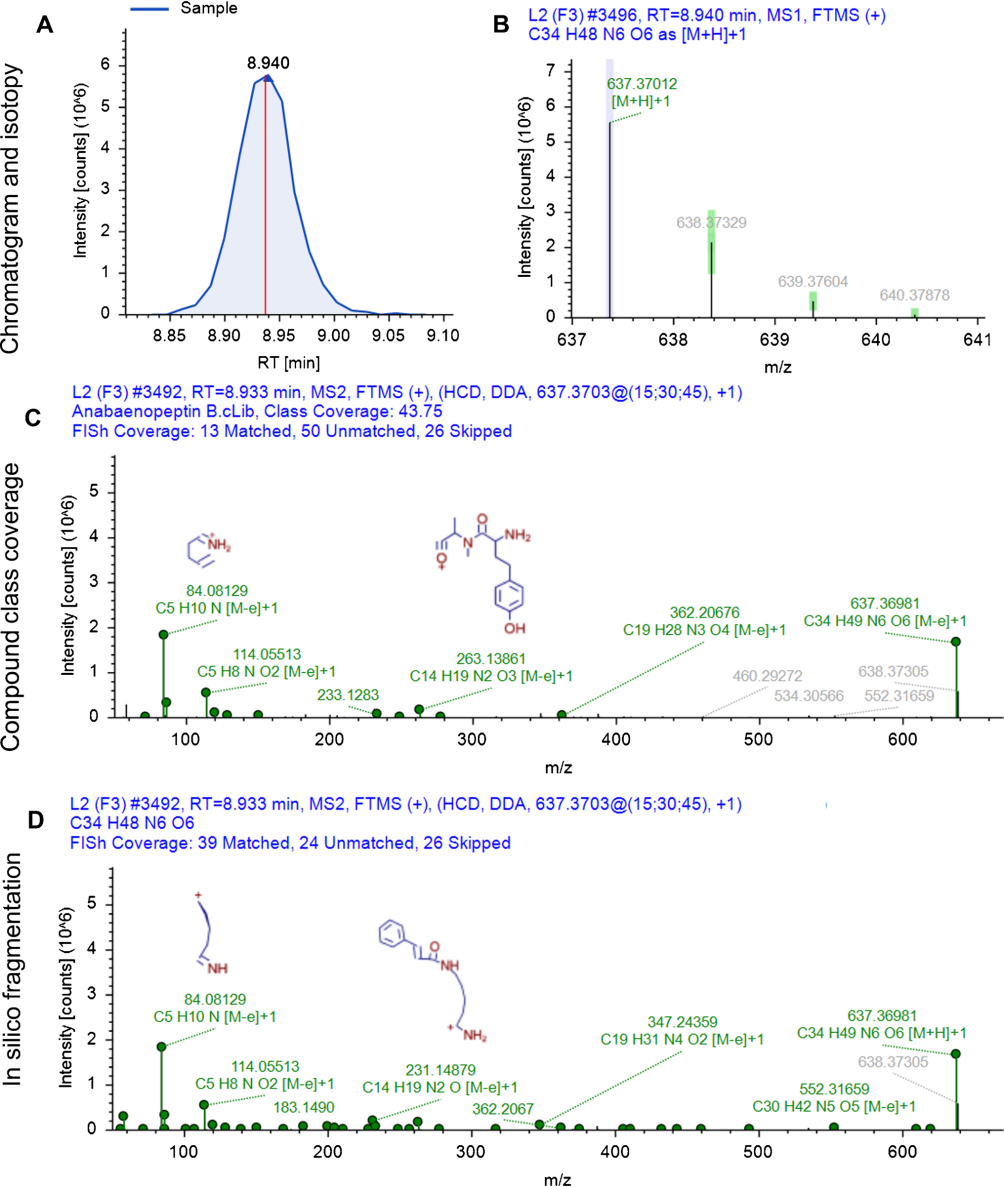
Example of structure characterization for the transformation product 1 (TP1). **A** Extracted ion chromatogram of ion *m/z* 637.3703, **B** isotopic pattern of most intense precursor ion, **C** fragmentation spectrum and compound class coverage compared to anabaenopeptin B, and **D** fragmentation spectrum and in silico matching with FISh coverage

The first TP (*m/z* 637.3703) in sample 11 likely results from the formation of carbamates (carbamatization) of anabaenopeptin B and anabaenopeptin C, involving the loss of the arginine chain and CO from the cyclic structure, with the configuration Lys^2^-Val^3^-HTyr^4^-Ala^5^-Phe^6^ (Fig. 2). The spectra (Fig. 3) share key fragments when compared to anabaenopeptin B used as standard in quality control, framing its structure within amino acid positions 2 to 6. However, key AA1 ions at *m/z* 129, 158, 175, and 201 are missing, suggesting the Arg group has been lost. TP2 (*m/z* 680.3755) is a proposed ketonization product common to anabaenopeptin B, -C, -E, and -SA3, found in samples 10 and 11 (Figs. S25 and S26). This TP likely corresponds to anabaenopeptin 679, a degradation product of anabaenopeptin SA3, based on the characteristic fragmentation pattern and retention time. Based on enviPath, this TP can originate from multiple anabaenopeptins with the cyclic structure Lys^2^-Val^3^-Hty^4^-MeAla^5^-Phe^6^, supporting the hypothesis that anabaenopeptin 679 is a degradation product of anabaenopeptin SA3. TP3 (*m/z* 695.3759), found in sample 11, is a putative carbamatization product of oscillamide Y, resulting in the loss of the AA1 side chain (Figs. S27 and S28). The spectrum shares the same key fragments as oscillamide Y, used as a standard in quality control, framing its structure within amino acid positions 2 to 6. However, the spectra lack key fragments at *m/z* 129, 158, 175, and 201, suggesting the loss of Tyr at AA1. TP4 (*m/z* 711.3710) is proposed as a product of two consecutive degradation reactions of oscillamide Y, identified in samples 7 and 11 (Figs. S29 and S30). First, hydroxylation at the tertiary aliphatic group was proposed in Ile at AA3, followed by carbamatization, leading to the loss of AA1. Spectra of TP4 closely resemble those of TP3, also lacking Tyr fragments.

TP5 (*m/z* 911.5712), found in samples 2 and 11, is proposed as a degradation product of [Asp^3^]microcystin-LR (Fig. S31). It forms through a three-step process (Fig. S32), including ring opening between AA6 and AA7. Only fragments below *m/z* 300 were detected, aligning with decarboxylation at AA3 and AA6 and demethylation at AA7. TP6 (*m/z* 999.5491) in sample 11 is a proposed linearization product of [Asp^3^]microcystin-LR, shown in Figs. S33 and S34. It represents a linear [seco-1/7][D-Asp^3^]microcystin-LR, which could be considered a potential new microcystin congener. The degradation pathway mirrors that described by Ding et al. for microcystin-LR [62]. TP7 (*m/z* 1061.5301), detected in samples 3 and 11 (Figs. S35 and S36), is a proposed hydroxylation product of microcystin-YR. Tyr at AA2 is hydroxylated to form dopamine, while the rest of the structure remains intact. The detection of dopamine formation from microcystin-YR is novel, suggesting a previously unrecognized transformation pathway or a new microcystin congener. Although enviPath suggests this compound as a TP, its identification remains uncertain due to the lack of information linking dopamine to the structure of microcystins. Therefore, this compound will be identified as a potential TP or metabolite, and further studies are needed to investigate the emergence of this form.

#### Level of confidence in identification for NTA

According to the Schymanski classification system, the TPs identified through this NTA method are assigned to Level 3, as their identification relies solely on MS^2^ data interpretation and context [42]. In NTA, the exact identity of the compound is not predetermined, and the identification relies on mass spectra, fragmentation patterns, and comparisons to existing databases or theoretical fragmentations. The identification of TPs at this level remains therefore tentative. However, this identification remains a preliminary proposal for the study of the transformation of these compound families, which are still very poorly understood. Further experimental validation and additional contextual evidence are necessary to confirm the structure of the identified TPs.

### Cyanopeptides discovery and methodology challenges

#### Cyanopeptides diversity

The discovery of a wide diversity of cyanopeptides in investigated waters uncovered both uncommon and novel congeners, as well as new TPs. Using the quantitative method with standards, a total of 14 cyanopeptides, including 8 microcystins, were identified across samples. By exploiting CyanoMetDB and in silico modeling for suspect screening and NTA, 22 uncommon cyanopeptides were detected, and 7 new TPs were identified through enviPath and compound class matching. Two new microcystin congeners, [DMAdda^5^, GluOMe^6^]microcystin-LHty and linear [seco-1/7][D-Asp^3^]microcystin-LR, were tentatively identified. Identifying new TPs, especially from less studied cyanopeptides like anabaenopeptins, highlights the need to study TPs to better understand their fate and ecological impact. This finding underscores the risks of focusing exclusively on well-characterized cyanopeptides in toxicological assessments. Furthermore, the identification of uncommon cyanopeptide families such as aeruginosins, cyanopeptolins, microginins, and anabaenopeptins challenges the conventional focus on microcystins. For instance, the notable prevalence of anabaenopeptins in the Yamaska River, compared to microcystins in Lake Saint-Pierre, suggests a shift in the distribution of cyanopeptides where the two watercourses differ in physico-chemical characteristics, likely due to various environmental factors. This discrepancy highlights the need for further investigation through expanded sampling and detailed cyanobacteria characterization (taxonomy and genomics) to refine our understanding of cyanopeptide distribution.

#### Methods comparison for cyanopeptides discovery

Our developed targeted and suspect screening methods are powerful tools for ensuring accurate quantification and confident identification of known and predicted cyanopeptides. Indeed, this study utilized one of the most extensive targeted methods for cyanopeptide analysis to date, quantifying 28 different compounds across seven families, in addition to a robust suspect screening approach that integrated in silico modeling to enhance the detection of predicted compounds within CyanoMetDB. Using this suspect screening methodology, we could indirectly identify a new congener, [DMAdda^5^, GluOMe^6^]microcystin-LHty. Unique spectral features that did not match the database were revealed when combined with manual interpretation. However, both approaches are limited by predefined compound lists; therefore, our developed NTA method complemented both targeted and suspect screening methods by broadening the scope of compound detection, discovering novel TPs and potential new congeners including [seco-1/7][Asp^3^]microcystin-LR and dopamine-modified microcystin-YR that would have otherwise gone undetected.

#### Challenges of in silico modeling

In silico modeling was employed to streamline and assist data interpretation but increased the risk of false positives without deeper spectral interpretation, due to similar chromatographic and spectral profiles. A minimum match score of 30% was required for in silico fragments; however, not all candidates met this threshold. The limitations were highlighted by the frequent absence of characteristic fragments. For example, in Tables S9 to S11, ions marked with an asterisk were not generated by FISh scoring but were key contributions to structural confirmation. This absence challenges accurate identification in complex matrices where overlapping signals may mask specific fragment ions. As a result, relying solely on computational predictions can yield incomplete or misleading results. This limitation was addressed using experimental data from known cyanopeptides, combined with expert interpretation to ensure better certainty in identification. In a previous study by Zervou et al., the use of in silico approaches has been explored without including standards, relying instead on a spectral database (mzCloud™) limited to a few microcystins congeners [34]. This strategy was successful for microcystins elucidation but restricted the exploration of CSMs due to the lack of available experimental data. Compound class matching with integrated experimental data also aided in discovering new cyanopeptides and studying previously unexplored TPs. Each cyanopeptide family shares a similar substructure, enabling the targeting of characteristic low- and medium-mass fragments, increasing confidence in structural identification within analogs. Future research on CSMs identification should focus on improving in silico algorithms and developing experimental open databases. Also, integrating databases with machine learning could improve identification accuracy, automate large dataset processing, and support toxicological studies and monitoring programs. This would expand geographic coverage and enable better risk assessments and management of newly identified compounds.

## Conclusion

In this study, we first optimized and applied a targeted method to quantify 28 cyanopeptides across seven families. The targeted method is one of the most extensive quantification methods for cyanopeptides to date and successfully quantified 14 known cyanopeptides with significant concentrations ranging from 0.038 to 5.68 µg L^−1^ in agriculture and urban-impacted water bodies. The suspect screening method, an approach that integrated in silico modeling and the CyanoMetDB open-source database, enabled the identification of 26 uncommon cyanopeptides including the characterization of a new microcystin, [DMAdda^5^, GluOMe^6^]microcystin-LHty. However, both targeted and suspect screening are limited by predefined compound lists; an NTA method was newly developed as a complement for broadening the scope of unknown compound detection. This approach successfully characterized 7 novel TPs and potential congeners including a new linear microcystin, [seco-1/7][Asp^3^]microcystin-LR, and a new dopamine form of microcystin-YR that would have otherwise gone undetected. These findings contribute significantly to the growing understanding of CSMs diversity in freshwater systems. The integration of suspect screening using CyanoMetDB and NTA with compound class matching, combined with in silico modeling, significantly enhanced the detection capabilities for both uncommon and unknown cyanopeptides. Furthermore, the use of the biotransformation prediction tool, enviPath, enabled a more effective identification of TPs in complex environmental samples. The detection of novel microcystins and other cyanopeptides raises important concerns about their potential ecological impact and human health risks, particularly in light of emerging evidence of additive toxicity from TPs and metabolites in HABs. This underscores the need for comprehensive screening protocols to improve the identification, characterization, and management of emerging toxins. The next steps in development should focus on improving the accuracy of in silico modeling and validating these methods with certified standards to expand the NTA capacity for complex CSMs mixtures. Additionally, future investigations should aim to expand the geographical scope of cyanopeptides monitoring and conduct experiments to assess the health risks associated with these newly identified compounds.

## Supplementary Information

Below is the link to the electronic supplementary material.

Supplementary file1 (DOCX 9.76 MB)

Supplementary file2 (DOCX 95 KB)

## Data Availability

All data are included in the paper.

## References

[1] Cottingham KL, Ewing HA, Greer ML, Carey CC, Weathers KC. Cyanobacteria as biological drivers of lake nitrogen and phosphorus cycling. Ecosphere. 2015;6:1–19. 10.1890/ES14-00174.1.

[2] Chorus I, Welker M. Toxic cyanobacteria in water: A Guide to their public health consequences, monitoring and management. 2nd ed. London: CRC Press; 2021.

[3] Zepernick BN, Wilhelm SW, Bullerjahn GS, Paerl HW. Climate change and the aquatic continuum: A cyanobacterial comeback story. Environ Microbiol Rep. 2023;15:3–12. 10.1111/1758-2229.13122. 36096485 10.1111/1758-2229.13122 PMC10103762

[4] Plaas HE, Paerl HW. Toxic cyanobacteria: A growing threat to water and air quality. Environ Sci Technol. 2021;55:44–64. 10.1021/acs.est.0c06653. 33334098 10.1021/acs.est.0c06653

[5] Jones MR, Pinto E, Torres MA, Dörr F, Mazur-Marzec H, Szubert K, Tartaglione L, Dell’Aversano C, Miles CO, Beach DG, McCarron P, Sivonen K, Fewer DP, Jokela J, Janssen EM-L. CyanoMetDB, a comprehensive public database of secondary metabolites from cyanobacteria. Water Res. 2021;196:117017. 10.1016/j.watres.2021.117017. 33765498 10.1016/j.watres.2021.117017

[6] Ito E, Takai A, Kondo F, Masui H, Imanishi S, Harada K. Comparison of protein phosphatase inhibitory activity and apparent toxicity of microcystins and related compounds. Toxicon. 2002;40:1017–25. 10.1016/S0041-0101(02)00099-5. 12076656 10.1016/s0041-0101(02)00099-5

[7] Du X, Liu H, Yuan L, Wang Y, Ma Y, Wang R, Chen X, Losiewicz M, Guo H, Zhang H. The diversity of cyanobacterial toxins on structural characterization, distribution and identification: A systematic review. Toxins. 2019;11:530. 10.3390/toxins11090530. 31547379 10.3390/toxins11090530 PMC6784007

[8] Janssen EM-L. Cyanobacterial peptides beyond microcystins – A review on co-occurrence, toxicity, and challenges for risk assessment. Water Res. 2019;151:488–99. 10.1016/j.watres.2018.12.048. 30641464 10.1016/j.watres.2018.12.048

[9] Pawlik-Skowrońska B, Bownik A, Pogorzelec M, Kulczycka J, Sumińska A. First report on adverse effects of cyanobacterial anabaenopeptins, aeruginosins, microginin and their mixtures with microcystin and cylindrospermopsin on aquatic plant physiology: An experimental approach. Toxicon. 2023;107333. 10.1016/j.toxicon.2023.107333 10.1016/j.toxicon.2023.107333 37951248

[10] Silva-Stenico ME, Silva CSP, Lorenzi AS, Shishido TK, Etchegaray A, Lira SP, Moraes LAB, Fiore MF. Non-ribosomal peptides produced by Brazilian cyanobacterial isolates with antimicrobial activity. Microbiol Res. 2011;166:161–75. 10.1016/j.micres.2010.04.002. 20630723 10.1016/j.micres.2010.04.002

[11] Ahmad S, Saleem M, Riaz N, Lee YS, Diri R, Noor A, Almasri D, Bagalagel A, Elsebai MF. The natural polypeptides as significant elastase inhibitors. Front Pharmacol. 2020;11:688. 10.3389/fphar.2020.00688. 32581778 10.3389/fphar.2020.00688 PMC7291377

[12] Chorus I, Fastner J, Welker M. Cyanobacteria and cyanotoxins in a changing environment: concepts, controversies, challenges. Water. 2021;13:2463. 10.3390/w13182463.

[13] Wang S, Zhang X, Wang C, Chen N. Multivariable integrated risk assessment for cyanobacterial blooms in eutrophic lakes and its spatiotemporal characteristics. Water Res. 2023;228:119367. 10.1016/j.watres.2022.119367. 36417795 10.1016/j.watres.2022.119367

[14] Beversdorf L, Weirich C, Bartlett S, Miller T. Variable Cyanobacterial Toxin and Metabolite profiles across six eutrophic lakes of differing physiochemical characteristics. Toxins. 2017;9:62. 10.3390/toxins9020062. 28208628 10.3390/toxins9020062 PMC5331441

[15] Bartlett SL, Brunner SL, Klump JV, Houghton EM, Miller TR. Spatial analysis of toxic or otherwise bioactive cyanobacterial peptides in Green Bay. Lake Michigan J Great Lakes Res. 2018;44:924–33. 10.1016/j.jglr.2018.08.016. 30983692 10.1016/j.jglr.2018.08.016 PMC6456082

[16] Roy-Lachapelle A, Vo Duy S, Munoz G, Dinh QT, Bahl E, Simon DF, Sauvé S. Analysis of multiclass cyanotoxins (microcystins, anabaenopeptins, cylindrospermopsin and anatoxins) in lake waters using on-line SPE liquid chromatography high-resolution Orbitrap mass spectrometry. Anal Methods. 2019;11:5289–300. 10.1039/C9AY01132C.

[17] Zervou S-K, Kaloudis T, Gkelis S, Hiskia A, Mazur-Marzec H. Anabaenopeptins from cyanobacteria in freshwater bodies of Greece. Toxins. 2021;14:4. 10.3390/toxins14010004. 35050981 10.3390/toxins14010004 PMC8781842

[18] Romera-García E, Helmus R, Ballesteros-Gómez A, Visser PM. Multi-class determination of intracellular and extracellular cyanotoxins in freshwater samples by ultra-high performance liquid chromatography coupled to high resolution mass spectrometry. Chemosphere. 2021;274:129770. 10.1016/j.chemosphere.2021.129770. 33549883 10.1016/j.chemosphere.2021.129770

[19] You L, Tong X, Te SH, Tran NH, bteSukarji NH, He Y, Gin KY-H. Multi-class secondary metabolites in cyanobacterial blooms from a tropical water body: Distribution patterns and real-time prediction. Water Res. 2022;212:118129. 10.1016/j.watres.2022.118129. 35121419 10.1016/j.watres.2022.118129

[20] Zervou S-K, Moschandreou K, Paraskevopoulou A, Christophoridis C, Grigoriadou E, Kaloudis T, Triantis TM, Tsiaoussi V, Hiskia A. Cyanobacterial toxins and peptides in Lake Vegoritis. Greece Toxins. 2021;13:394. 10.3390/toxins13060394. 34205997 10.3390/toxins13060394 PMC8230288

[21] Jones M, Janssen E. Quantification of multi-class cyanopeptides in Swiss Lakes with automated extraction, enrichment and analysis by online-SPE HPLC-HRMS/MS. Chimia. 2022;76:133. 10.2533/chimia.2022.133. 38069759 10.2533/chimia.2022.133

[22] Hollender J, Schymanski EL, Ahrens L, Alygizakis N, Béen F, Bijlsma L, Brunner AM, Celma A, Fildier A, Fu Q, Gago-Ferrero P, Gil-Solsona R, Haglund P, Hansen M, Kaserzon S, Kruve A, Lamoree M, Margoum C, Meijer J, Merel S, Rauert C, Rostkowski P, Samanipour S, Schulze B, Schulze T, Singh RR, Slobodnik J, Steininger-Mairinger T, Thomaidis NS, Togola A, Vorkamp K, Vulliet E, Zhu L, Krauss M. NORMAN guidance on suspect and non-target screening in environmental monitoring. Environ Sci Eur. 2023;35:75. 10.1186/s12302-023-00779-4.

[23] Filatova D, Jones MR, Haley JA, Núñez O, Farré M, Janssen EM-L. Cyanobacteria and their secondary metabolites in three freshwater reservoirs in the United Kingdom. Environ Sci Eur. 2021;33:29. 10.1186/s12302-021-00472-4.

[24] Varriale F, Tartaglione L, Zervou S-K, Miles CO, Mazur-Marzec H, Triantis TM, Kaloudis T, Hiskia A, Dell’Aversano C. Untargeted and targeted LC-MS and data processing workflow for the comprehensive analysis of oligopeptides from cyanobacteria. Chemosphere. 2023;311:137012. 10.1016/j.chemosphere.2022.137012. 36397634 10.1016/j.chemosphere.2022.137012

[25] Otto JFM, Kiel C, Nejstgaard JC, Pohnert G, Berger SA, Ueberschaar N. Tracking a broad inventory of cyanotoxins and related secondary metabolites using UHPLC-HRMS. J Hazardous Mater Adv. 2023;12:100370. 10.1016/j.hazadv.2023.100370.

[26] Janssen EM-L, Jones MR, Pinto E, Dörr F, Torres MA, Rios Jacinavicius F, Mazur-Marzec H, Szubert K, Konkel R, Tartaglione L, Dell’Aversano C, Miglione A, McCarron P, Beach DG, Miles CO, Fewer DP, Sivonen K, Jokela J, Wahlsten M, Niedermeyer THJ, Schanbacher F, Leão P, Preto M, D’Agostino PM, Baunach M, Dittmann E, Reher R. S75 | CyanoMetDB | Comprehensive database of secondary metabolites from cyanobacteria. 2023. 10.5281/zenodo.7922070.

[27] Picardo M, Sanchís J, Núñez O, Farré M. Suspect screening of natural toxins in surface and drinking water by high performance liquid chromatography and high-resolution mass spectrometry. Chemosphere. 2020;261:127888. 10.1016/j.chemosphere.2020.127888. 33113669 10.1016/j.chemosphere.2020.127888

[28] Ortiz X, Korenkova E, Jobst KJ, MacPherson KA, Reiner EJ. A high throughput targeted and non-targeted method for the analysis of microcystins and anatoxin-A using on-line solid phase extraction coupled to liquid chromatography–quadrupole time-of-flight high resolution mass spectrometry. Anal Bioanal Chem. 2017;409:4959–69. 10.1007/s00216-017-0437-0. 28634756 10.1007/s00216-017-0437-0

[29] Roy-Lachapelle A, Solliec M, Sauvé S, Gagnon C. A data-independent methodology for the structural characterization of microcystins and anabaenopeptins leading to the identification of four new congeners. Toxins. 2019;11:619. 10.3390/toxins11110619. 31717734 10.3390/toxins11110619 PMC6891544

[30] Baliu-Rodriguez D, Peraino NJ, Premathilaka SH, Birbeck JA, Baliu-Rodriguez T, Westrick JA, Isailovic D. Identification of novel microcystins using high-resolution MS and MS ^*n*^ with Python code. Environ Sci Technol. 2022;56:1652–63. 10.1021/acs.est.1c04296. 35018784 10.1021/acs.est.1c04296 PMC12989180

[31] Kim Tiam S, Gugger M, Demay J, Le Manach S, Duval C, Bernard C, Marie B. Insights into the diversity of secondary metabolites of *Planktothrix* using a biphasic approach combining global genomics and metabolomics. Toxins. 2019;11:498. 10.3390/toxins11090498. 31461939 10.3390/toxins11090498 PMC6784222

[32] Kust A, Řeháková K, Vrba J, Maicher V, Mareš J, Hrouzek P, Chiriac M-C, Benedová Z, Tesařová B, Saurav K. Insight into unprecedented diversity of cyanopeptides in eutrophic ponds using an MS/MS networking approach. Toxins. 2020;12:561. 10.3390/toxins12090561. 32878042 10.3390/toxins12090561 PMC7551678

[33] Saha S, Esposito G, Urajová P, Mareš J, Ewe D, Caso A, Macho M, Delawská K, Kust A, Hrouzek P, Juráň J, Costantino V, Saurav K. Discovery of unusual cyanobacterial tryptophan-containing anabaenopeptins by MS/MS-based molecular networking. Molecules. 2020;25:3786. 10.3390/molecules25173786. 32825321 10.3390/molecules25173786 PMC7503407

[34] Zervou S-K, Hammoud NA, Godin S, Hiskia A, Szpunar J, Lobinski R. Detection of secondary cyanobacterial metabolites using LC-HRMS in Lake Karaoun. Sci Total Environ. 2023;892:164725. 10.1016/j.scitotenv.2023.164725. 37290649 10.1016/j.scitotenv.2023.164725

[35] Mashile GP, Nomngongo PN. Recent Application of Solid Phase Based Techniques for Extraction and Preconcentration of Cyanotoxins in Environmental Matrices. Crit Rev Anal Chem. 2017;47:119–26. 10.1080/10408347.2016.1225255. 27541433 10.1080/10408347.2016.1225255

[36] Molinspiration Cheminformatics. Calculation of molecular properties and bioactivity score. 2025. http://www.molinspiration com/cgi-bin/properties. Accessed 13 Jan 2025.

[37] Ersmark K, Del Valle JR, Hanessian S. Chemistry and biology of the aeruginosin family of serine protease inhibitors. Angew Chem Int Ed. 2008;47:1202–23. 10.1002/anie.200605219. 10.1002/anie.200605219 18076006

[38] Puddick J, Prinsep M, Wood S, Kaufononga S, Cary S, Hamilton D. High levels of structural diversity observed in microcystins from *Microcystis* CAWBG11 and characterization of six new microcystin congeners. Mar Drugs. 2014;12:5372–95. 10.3390/md12115372. 25402827 10.3390/md12115372 PMC4245536

[39] Spoof L, Błaszczyk A, Meriluoto J, Cegłowska M, Mazur-Marzec H. Structures and activity of new anabaenopeptins produced by Baltic Sea cyanobacteria. Mar Drugs. 2015;14:8. 10.3390/md14010008. 26729139 10.3390/md14010008 PMC4728505

[40] Bogialli S, Bortolini C, Di Gangi IM, Di Gregorio FN, Lucentini L, Favaro G, Pastore P. Liquid chromatography-high resolution mass spectrometric methods for the surveillance monitoring of cyanotoxins in freshwaters. Talanta. 2017;170:322–30. 10.1016/j.talanta.2017.04.033. 28501176 10.1016/j.talanta.2017.04.033

[41] Bouaïcha N, Miles C, Beach D, Labidi Z, Djabri A, Benayache N, Nguyen-Quang T. Structural diversity, characterization and toxicology of microcystins. Toxins. 2019;11:714. 10.3390/toxins11120714. 31817927 10.3390/toxins11120714 PMC6950048

[42] Schymanski EL, Jeon J, Gulde R, Fenner K, Ruff M, Singer HP, Hollender J. Identifying small molecules via high resolution mass spectrometry: communicating confidence. Environ Sci Technol. 2014;48:2097–8. 10.1021/es5002105. 24476540 10.1021/es5002105

[43] Wicker J, Lorsbach T, Gütlein M, Schmid E, Latino D, Kramer S, Fenner K. enviPath – The environmental contaminant biotransformation pathway resource. Nucleic Acids Res. 2016;44:D502–8. 10.1093/nar/gkv1229. 26582924 10.1093/nar/gkv1229 PMC4702869

[44] Hafner J, Lorsbach T, Schmidt S, Brydon L, Dost K, Zhang K, Fenner K, Wicker JS. Advancements in biotransformation pathway prediction: enhancements, datasets, and novel functionalities in enviPath. J Cheminform. 2024;16(1):93. 10.1186/s13321-024-00881-6. 39107805 10.1186/s13321-024-00881-6 PMC11304562

[45] Miles CO, Sandvik M, Nonga HE, Rundberget T, Wilkins AL, Rise F, Ballot A. Thiol derivatization for LC-MS identification of microcystins in complex matrices. Environ Sci Technol. 2012;46:8937–44. 10.1021/es301808h. 22834560 10.1021/es301808h

[46] Lévesque D, Cattaneo A, Hudon C, Gagnon P. Predicting the risk of proliferation of the benthic cyanobacterium *Lyngbya wollei* in the St. Lawrence River. Can J Fish Aquat Sci. 2012;69:1585–95. 10.1139/f2012-087.

[47] Flores C, Caixach J. High levels of anabaenopeptins detected in a cyanobacteria bloom from NE spanish Sau-Susqueda-El Pasteral reservoirs system by LC–HRMS. Toxins. 2020;12:541. 32842578 10.3390/toxins12090541 PMC7551688

[48] Choi BW, Namikoshi M, Sun F, Rinehart KL, Carmichael WW, Kaup AM, Evans WR, Beasley VR. Isolation of linear peptides related to the hepatotoxins nodularin and microcystins. Tetrahedron Lett. 1993;34:7881–4. 10.1016/S0040-4039(00)61500-9.

[49] Namikoshi M, Sun F, Choi BW, Rinehart KL, Carmichael WW, Evans WR, Beasley VR. Seven more microcystins from Homer lake cells: Application of the general method for structure assignment of peptides containing.alpha.,beta.-dehydroamino acid unit(s). J Org Chem. 1995;60:3671–9. 10.1021/jo00117a017.

[50] Miles CO, Melanson JE, Ballot A. Sulfide Oxidations for LC-MS Analysis of methionine-containing microcystins in *Dolichospermum flos-aquae* NIVA-CYA 656. Environ Sci Technol. 2014;48:13307–15. 10.1021/es5029102. 25333659 10.1021/es5029102

[51] Kondo F, Ikai Y, Oka H, Okumura M, Ishikawa N, Harada K, Matsuura K, Murata H, Suzuki M. Formation, characterization, and toxicity of the glutathione and cysteine conjugates of toxic heptapeptide microcystins. Chem Res Toxicol. 1992;5:591–6. 10.1021/tx00029a002. 1445998 10.1021/tx00029a002

[52] Itou Y, Suzuki S, Ishida K, Murakami M. Anabaenopeptins G and H, potent carboxypeptidase A inhibitors from the cyanobacterium *Oscillatoria agardhii* (NIES-595). Bioorg Med Chem Lett. 1999;9:1243–6. 10.1016/S0960-894X(99)00191-2. 10340607 10.1016/s0960-894x(99)00191-2

[53] Gesner-Apter S, Carmeli S. Protease inhibitors from a water bloom of the cyanobacterium *Microcystis aeruginosa*. J Nat Prod. 2009;72:1429–36. 10.1021/np900340t. 19650639 10.1021/np900340t

[54] Monteiro PR, doAmaral SC, Siqueira AS, Xavier LP, Santos AV. Anabaenopeptins: What we know so far. Toxins. 2021;13:522. 10.3390/toxins13080522. 34437393 10.3390/toxins13080522 PMC8402340

[55] Harms H, Kurita KL, Pan L, Wahome PG, He H, Kinghorn AD, Carter GT, Linington RG. Discovery of anabaenopeptin 679 from freshwater algal bloom material: Insights into the structure–activity relationship of anabaenopeptin protease inhibitors. Bioorg Med Chem Lett. 2016;26:4960–5. 10.1016/j.bmcl.2016.09.008. 27641470 10.1016/j.bmcl.2016.09.008

[56] Zervou S-K, Gkelis S, Kaloudis T, Hiskia A, Mazur-Marzec H. New microginins from cyanobacteria of Greek freshwaters. Chemosphere. 2020;248:125961. 10.1016/j.chemosphere.2020.125961. 32059332 10.1016/j.chemosphere.2020.125961

[57] Harada K, Mayumi T, Shimada T, Suzuki M, Kondo F, Watanabe MF. Occurrence of four depsipeptides, aeruginopeptins, together with microcystins from toxic cyanobacteria. Tetrahedron Lett. 1993;34:6091–4. 10.1016/S0040-4039(00)61736-7.

[58] Faltermann S, Zucchi S, Kohler E, Blom JF, Pernthaler J, Fent K. Molecular effects of the cyanobacterial toxin cyanopeptolin (CP1020) occurring in algal blooms: Global transcriptome analysis in zebrafish embryos. Aquat Toxicol. 2014;149:33–9. 10.1016/j.aquatox.2014.01.018. 24561424 10.1016/j.aquatox.2014.01.018

[59] Lenz KA, Miller TR, Ma H. Anabaenopeptins and cyanopeptolins induce systemic toxicity effects in a model organism the nematode Caenorhabditis elegans. Chemosphere. 2019;214:60–9. 10.1016/j.chemosphere.2018.09.076. 30253257 10.1016/j.chemosphere.2018.09.076

[60] Bober B, Chrapusta-Srebrny E, Bialczyk J. Novel cyanobacterial metabolites, cyanopeptolin 1081 and anabaenopeptin 899, isolated from an enrichment culture dominated by *Woronichinia naegeliana* (Unger) Elenkin. Eur J Appl Physiol. 2021;56:244–54. 10.1080/09670262.2020.1813809.

[61] Liu J, Zhang M, Huang Z, Fang J, Wang Z, Zhou C, Qiu X. Diversity, biosynthesis and bioactivity of aeruginosins, a family of vyanobacteria-derived nonribosomal linear tetrapeptides. Mar Drugs. 2023;21:217. 10.3390/md21040217. 37103356 10.3390/md21040217 PMC10143770

[62] Ding Q, Liu K, Xu K, Sun R, Zhang J, Yin L, Pu Y. Further understanding of degradation pathways of microcystin-LR by an indigenous *Sphingopyxis* sp. in environmentally relevant pollution concentrations. Toxins. 2018;10:536. 10.3390/toxins10120536. 30558170 10.3390/toxins10120536 PMC6315713

