# Supplementary material for: Characterizing cyanopeptides and transformation products in freshwater: integrating targeted, suspect, and non-targeted analysis with in silico modeling
Source: Anal Bioanal Chem. 2025 Jul 12;417(21):4829–46. doi: 10.1007/s00216-025-05999-6 (PMC12367966; doi:10.1007/s00216-025-05999-6)
Supplement: Supplementary file 1 — Supplementary file1 (DOCX 9.76 MB) [file 216_2025_5999_MOESM1_ESM.docx]

**SUPPLEMENTARY INFORMATION**

*Page S-5, Figure S1.* Sampling sites for Lac Saint-Pierre and Yamaska River.

*Page S-6, Table S1*. Validation parameters of the quantitative method including method detection and quantification limits (MDL and MQL), extraction recoveries and matrix effects.

*Page S-7, Figure S2*. Chromatographic gradient conditions for the quantitative analysis workflow (QqQ) and the suspect screening and non-targeted analysis workflow (HRMS).

*Page S-8, Table S2*. Ionization and ion selection parameters for the quantitative analysis workflow (QqQ) and the suspect screening and non-targeted analysis workflow (HRMS).

*Page S-9, Table S3*. Selected reaction monitoring (SRM) parameters for targeted compounds quantitative analysis.

*Page S-11, Table S4*. Validation parameters of the quantitative method including accuracy and precision.

*Page S-12, Table S5*. Compound Discoverer 3.3 workflows.

*Page S-13, Table S6*. Calculated Log P of targeted compounds.

*Page S-14, Figure S3.* Chemical structures of microcystins (MC-LR), anabaenopeptins (AP-A) and other found cyanopeptides : Microginin 690, Aeruginopeptin 228A, Cyanopeptolin 1081 and Aeruginosin A.

*Page S-15, Table S7*. Microcystins (MCs) specific ions from MS^2^ fragmentation.

*Page S-16, Figure S4*. Structure characterization of [Ser^7^]microcystin-LR. A) Extracted ion chromatogram of ion *m/z* 999.5503 and B) isotopic pattern of most intense precursor ion. C) Fragmentation spectrum and *in-silico* matching with FISh coverage.

*Page S-17, SI-2*. Spectral interpretation of [DMAdda^5^, GluOMe^6^]microcystin-LHty.

*Page S-18, Figure S5*. Structure characterization of [seco-4/5][D-Asp^3^]microcystin-HtyR. A) Extracted ion chromatogram of ion *m/z* 1063.5370 and B) isotopic pattern of most intense precursor ion. C) Extracted ion chromatogram of thiol derivative ion *m/z* 1141.5552 and D) isotopic pattern of thiol derivative. E) Fragmentation spectrum and *in-silico* matching with FISh coverage.

*Page S-19, Figure S6*. Structure characterization of [D-Asp^3^]microcystin-MR. A) Extracted ion chromatogram of ion *m/z* 999.4937 and B) isotopic pattern of most intense precursor ion. C) Extracted ion chromatogram of thiol derivative ion *m/z* 1077.5037 and D) isotopic pattern of thiol derivative. E) Fragmentation spectrum and *in-silico* matching with FISh coverage.

*Page S-20, Figure S7*. Structure characterization of [D-Asp^3^]microcystin-M(O)R. A) Extracted ion chromatogram of ion *m/z* 1015.4898 and B) isotopic pattern of most intense precursor ion. C) Extracted ion chromatogram of thiol derivative ion *m/z* 1093.4993 and D) isotopic pattern of thiol derivative. E) Fragmentation spectrum and *in-silico* matching with FISh coverage.

*Page S-21, Figure S8*. Structure characterization of [DMAdda^5^]microcystin-YR. A) Extracted ion chromatogram of ion *m/z* 1031.5168 and B) isotopic pattern of most intense precursor ion. C) Extracted ion chromatogram of thiol derivative ion *m/z* 1109.5294 and D) isotopic pattern of thiol derivative. E) Fragmentation spectrum and *in-silico* matching with FISh coverage.

*Page S-22, Figure S9*. Structure characterization of [D-Ser^1^, D-Asp^3^]microcystin-HtyR. A) Extracted ion chromatogram of ion *m/z* 1061.5292 and B) isotopic pattern of most intense precursor ion. C) Extracted ion chromatogram of thiol derivative ion *m/z* 1139.5392 and D) isotopic pattern of thiol derivative. E) Fragmentation spectrum and *in-silico* matching with FISh coverage.

*Page S-23, Figure S10*. Structure characterization of [D-Ser^1^, D-Asp^3^]microcystin-HtyR. A) Extracted ion chromatogram of ion *m/z* 967.5246 and B) isotopic pattern of most intense precursor ion. C) Extracted ion chromatogram of thiol derivative ion *m/z* 1045.5334 and D) isotopic pattern of thiol derivative. E) Fragmentation spectrum and *in-silico* matching with FISh coverage.

*Page S-24, Figure S11*. Structure characterization of [Mdha-GSH^7^]microcystin-LR. A) Extracted ion chromatogram of ion *m/z* 1302.6398 and B) isotopic pattern of most intense precursor ion. C) Fragmentation spectrum and *in-silico* matching with FISh coverage.

*Page S-25, Figure S12*. Structure characterization of [epoxyAdda^5^]microcystin-LR. A) Extracted ion chromatogram of ion *m/z* 1011.5501 and B) isotopic pattern of most intense precursor ion. C) Extracted ion chromatogram of thiol derivative ion *m/z* 1089.5625 and D) isotopic pattern of thiol derivative. E) Fragmentation spectrum and *in-silico* matching with FISh coverage.

*Page S-26, Figure S13*. Structure characterization of [DMAdda^5^]microcystin-LR. A) Extracted ion chromatogram of ion *m/z* 981.5108 and B) isotopic pattern of most intense precursor ion. C) Extracted ion chromatogram of thiol derivative ion *m/z* 1059.5524 and D) isotopic pattern of thiol derivative. E) Fragmentation spectrum and *in-silico* matching with FISh coverage.

*Page S-27, Figure S14*. Structure characterization of [seco-1/2]microcystin-LR. A) Extracted ion chromatogram of ion *m/z* 1013.5672 and B) isotopic pattern of most intense precursor ion. C) Extracted ion chromatogram of thiol derivative ion *m/z* 1091.5706 and D) isotopic pattern of thiol derivative. E) Fragmentation spectrum and *in-silico* matching with FISh coverage.

*Page S-28, Table S8*. Anabaenopeptins (APs) specific ions from MS^2^ fragmentation (with associated sample).

*Page S-29, Figure S15*. Structure characterization of anabaenopeptin F. A) Extracted ion chromatogram of ion *m/z* 851.4758 and B) isotopic pattern of most intense precursor ion. E) Fragmentation spectrum and *in-silico* matching with FISh coverage.

*Page S-30, SI-3*. Spectral interpretation of anabaenopeptin F.

*Page S-31, Figure S16*. Structure characterization of anabaenopeptin E. A) Extracted ion chromatogram of ion *m/z* 851.4755 and B) isotopic pattern of most intense precursor ion. E) Fragmentation spectrum and *in-silico* matching with FISh coverage.

*Page S-32, Figure S17*. Structure characterization of anabaenopeptin H. A) Extracted ion chromatogram of ion *m/z* 923.5311 and B) isotopic pattern of most intense precursor ion. E) Fragmentation spectrum and *in-silico* matching with FISh coverage.

*Page S-33, Figure S18*. Structure characterization of anabaenopeptin HU892. A) Extracted ion chromatogram of ion *m/z* 893.5226 and B) isotopic pattern of most intense precursor ion. E) Fragmentation spectrum and *in-silico* matching with FISh coverage.

*Page S-34, Figure S19*. Structure characterization of anabaenopeptin SA3. A) Extracted ion chromatogram of ion *m/z* 823.4703 and B) isotopic pattern of most intense precursor ion. E) Fragmentation spectrum and *in-silico* matching with FISh coverage.

*Page S-35, Figure S20*. Structure characterization of anabaenopeptin 679. A) Extracted ion chromatogram of ion *m/z* 637.3700 and B) isotopic pattern of most intense precursor ion. E) Fragmentation spectrum and *in-silico* matching with FISh coverage.

*Page S-36, Table S9*. Other cyanopeptides specific ions from MS^2^ fragmentation.

*Page S-37, Figure S21*. Structure characterization of microginin 690. A) Extracted ion chromatogram of ion *m/z* 691.3350 and B) isotopic pattern of most intense precursor ion. E) Fragmentation spectrum and *in-silico* matching with FISh coverage.

*Page S-38, Figure S22*. Structure characterization of aeruginopeptin 228A. A) Extracted ion chromatogram of ion *m/z* 1045.4854 and B) isotopic pattern of most intense precursor ion. E) Fragmentation spectrum and *in-silico* matching with FISh coverage.

*Page S-39, Figure S23*. Structure characterization of cyanopeptolin 1081. A) Extracted ion chromatogram of ion *m/z* 1082.5393 and B) isotopic pattern of most intense precursor ion. E) Fragmentation spectrum and *in-silico* matching with FISh coverage.

*Page S-40, Figure S24*. Structure characterization of aeruginosin A. A) Extracted ion chromatogram of ion *m/z* 617.3458 and B) isotopic pattern of most intense precursor ion. E) Fragmentation spectrum and *in-silico* matching with FISh coverage.

*Page S-41, Figure S25*. Structure characterization of transformation product 2 (TP2). A) Extracted ion chromatogram of ion *m/z* 680.3755, B) isotopic pattern of most intense precursor ion, C) Fragmentation spectrum and compound class coverage compared to anabaenopeptin B and D) Fragmentation spectrum and *in-silico* matching with FISh coverage.

*Page S-42, Figure S26*. Proposed transformation product 2 (TP2) formation by ketonization of anabaenopeptin B.

*Page S-43, Figure S27*. Structure characterization of transformation product 3 (TP3). A) Extracted ion chromatogram of ion *m/z* 695.3759, B) isotopic pattern of most intense precursor ion, C) Fragmentation spectrum and compound class coverage compared to oscillamide Y and D) Fragmentation spectrum and *in-silico* matching with FISh coverage.

*Page S-44, Figure S28*. Proposed transformation product 3 (TP3) formation by carbamatization of oscillamide Y.

*Page S-45, Figure S29*. Structure characterization of transformation product 4 (TP4). A) Extracted ion chromatogram of ion *m/z* 711.3710, B) isotopic pattern of most intense precursor ion, C) Fragmentation spectrum and compound class coverage compared to oscillamide Y and D) Fragmentation spectrum and *in-silico* matching with FISh coverage.

*Page S-46, Figure S30*. Proposed transformation product 4 (TP4) formation in two steps of oscillamide Y.

*Page S-47, Figure S31*. Structure characterization of transformation product 5 (TP5). A) Extracted ion chromatogram of ion *m/z* 911.5712, B) isotopic pattern of most intense precursor ion, C) Extracted ion chromatogram of thiol derivative ion *m/z* 989.5826 and D) isotopic pattern of thiol derivative. E) Fragmentation spectrum and compound class coverage compared to [Asp^3^]microcystin-LR and F) Fragmentation spectrum and *in-silico* matching with FISh coverage.

*Page S-48, Figure S32*. Proposed transformation product 5 (TP5) formation in two steps of [Asp^3^]microcystin-LR.

*Page S-49, Figure S33*. Structure characterization of transformation product 6 (TP6). A) Extracted ion chromatogram of ion *m/z* 999.5491, B) isotopic pattern of most intense precursor ion, C) Extracted ion chromatogram of thiol derivative ion *m/z* 1077.5518 and D) isotopic pattern of thiol derivative. E) Fragmentation spectrum and compound class coverage compared to [Asp^3^]microcystin-LR and F) Fragmentation spectrum and *in-silico* matching with FISh coverage.

*Page S-50, Figure S34*. Proposed transformation product 6 (TP6) formation by linearization of [Asp^3^]microcystin-LR.

*Page S-51, Figure S35*. Structure characterization of transformation product 7 (TP7). A) Extracted ion chromatogram of ion *m/z* 1061.5301, B) isotopic pattern of most intense precursor ion, C) Extracted ion chromatogram of thiol derivative ion *m/z* 1139.5427 and D) isotopic pattern of thiol derivative. E) Fragmentation spectrum and compound class coverage compared to microcystin-YR and F) Fragmentation spectrum and *in-silico* matching with FISh coverage.

*Page S-52, Figure S36*. Proposed transformation product 7 (TP7) formation by linearization of microcystin-YR.

*Page S-53*. References

**
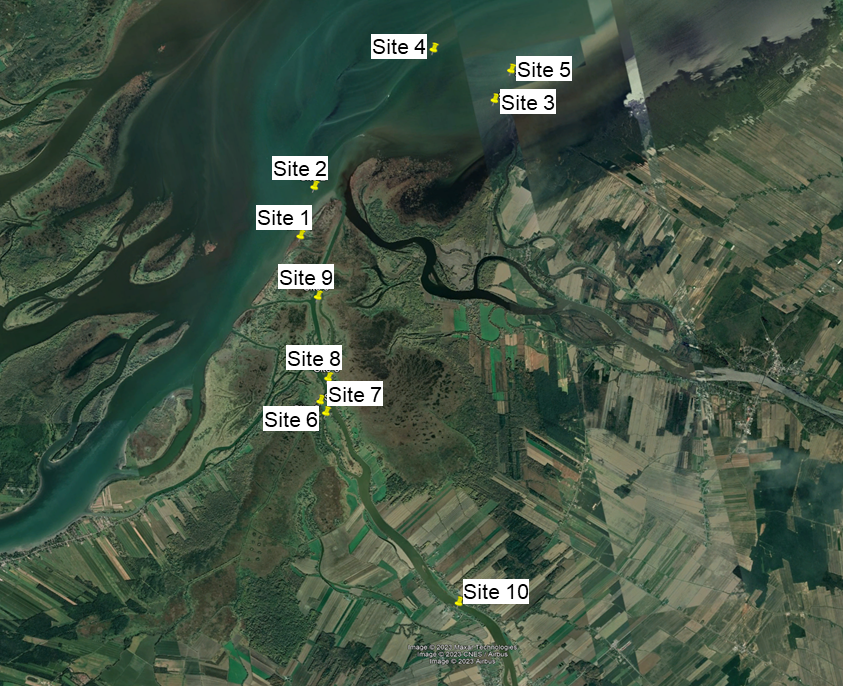
**

**Figure S1**. Sampling sites for Lac Saint-Pierre and Yamaska River

**Table S1.** Validation parameters of the quantitative method including method detection and quantification limits (MDL and MQL), extraction recovery and matrix effects.

| **Compounds** | ***R^2^*** | **MDL**  **(ng L^-1^)** | **MQL**  **(ng L^-1^)** | **Extraction**  **recoveries (%)** | **Matrix effects (%)** |
| --- | --- | --- | --- | --- | --- |
| AG-98A | 0.9995 | 7 | 22 | 76 ± 3 | 88 ± 2 |
| AG-98B | 0.9997 | 4 | 12 | 75 ± 4 | 82 ± 2 |
| AGU-98A | 0.9986 | 11 | 35 | 89 ± 3 | 93 ± 2 |
| AP-A | 0.9999 | 4 | 13 | 107 ± 2 | 90 ± 1 |
| AP-B | 0.9997 | 4 | 12 | 110 ± 7 | 87 ± 5 |
| AP-C | 0.9999 | 9 | 31 | 110 ± 4 | 90 ± 3 |
| AP-J | 0.9999 | 10 | 33 | 108 ± 5 | 89 ± 3 |
| AP-915 | 0.9996 | 8 | 25 | 105 ± 3 | 95 ± 7 |
| FA-A | 0.9999 | 8 | 25 | 92 ± 4 | 86 ± 2 |
| OC-Y | 0.9999 | 3 | 9 | 103 ± 3 | 98 ± 6 |
| CP-1041 | 0.9961 | 8 | 26 | 54 ± 5 | 86 ± 3 |
| [Asp^3^]MC-RR | 0.9999 | 3 | 9 | 81 ± 4 | 93 ± 3 |
| MC-RR | 0.9999 | 10 | 33 | 82 ± 6 | 89 ± 4 |
| MC-YR | 0.9998 | 8 | 26 | 104 ± 8 | 91 ± 5 |
| MC-HtyR | 0.9996 | 7 | 23 | 95 ± 7 | 100 ± 4 |
| [Asp^3^]MC-LR | 0.9999 | 5 | 16 | 110 ± 6 | 90 ± 3 |
| MC-LR | 0.9999 | 3 | 11 | 105 ± 3 | 90 ± 3 |
| MC-HilR | 0.9997 | 10 | 32 | 104 ± 9 | 89 ± 3 |
| MC-WR | 0.9998 | 10 | 32 | 110 ± 9 | 93 ± 3 |
| MC-LA | 0.9995 | 5 | 17 | 103 ± 8 | 89 ± 2 |
| MC-LY | 0.9998 | 8 | 28 | 99 ± 7 | 93 ± 5 |
| MC-LW | 0.9999 | 8 | 26 | 107 ± 9 | 103 ± 9 |
| MC-LF | 0.9999 | 9 | 28 | 89 ± 5 | 101 ± 2 |
| MG-527-ME | 0.9972 | 11 | 38 | 96 ± 3 | 92 ± 2 |
| MG-690-ME | 0.9978 | 7 | 23 | 58 ± 3 | 105 ± 3 |
| MG-FR1 | 0.9999 | 12 | 39 | 76 ± 3 | 91 ± 2 |
| MG-FR2 | 0.9999 | 9 | 30 | 96 ± 5 | 103 ± 5 |
| NOD-R | 0.9999 | 4 | 13 | 104 ± 7 | 88 ± 4 |

**Note:** The MDL and MQL were calculated by the standard deviation (SD) of five times the estimated MDL (0.5 ng L^-1^) : SD x 3.3 (MDL) and SD x 10 (MQL).

**
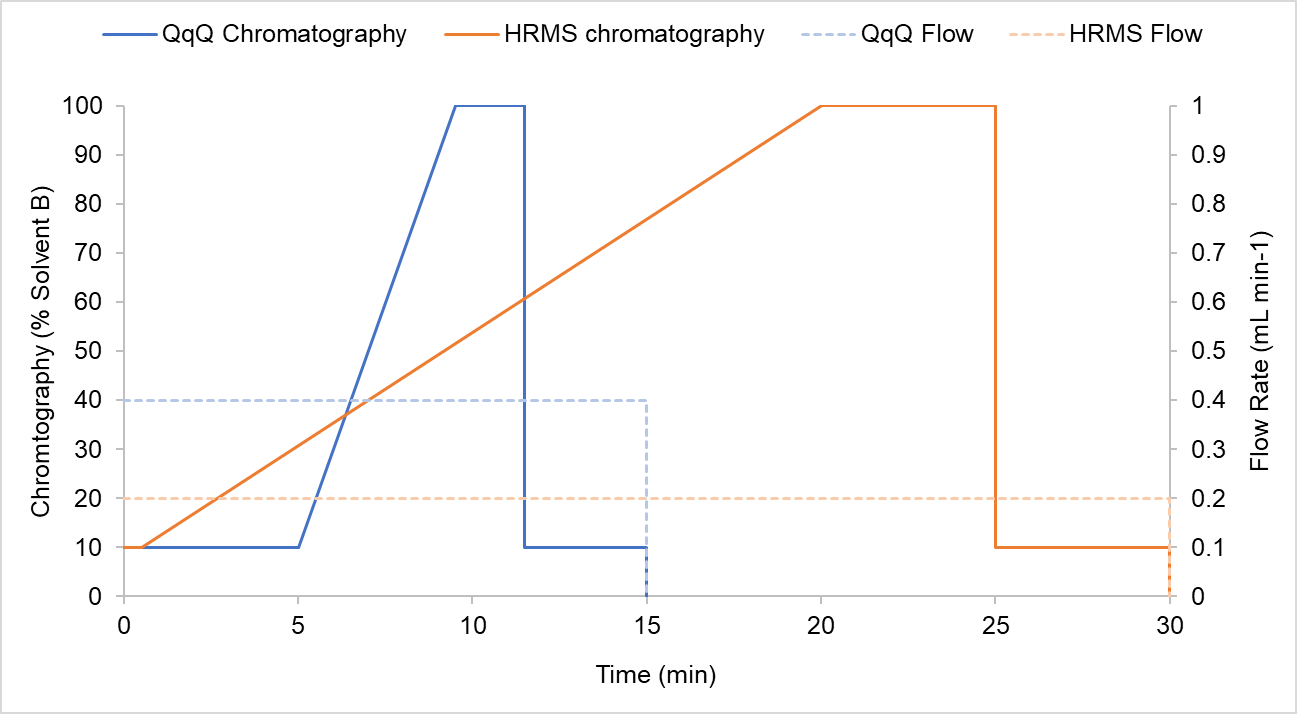
**

**Figure S2.** Chromatographic gradient conditions for the quantitative analysis workflow (QqQ) and the suspect screening and non-targeted analysis workflow (HRMS).

**Table S2.** Ionization and ion selection parameters for the quantitative analysis workflow (QqQ) and the suspect screening and non-targeted analysis workflow (HRMS).

| **QqQ** | | **HRMS** | |
| --- | --- | --- | --- |
| **Ionization parameters** | | | |
| Positive Ionization (V) | 3900 | 4000 | |
| Sheath Gas (Arbitrary) | 50 | 30 | |
| Auxiliary Gas (Arbitrary) | 10 | 10 | |
| Sweep Gas (Arbitrary) | 1 | 3 | |
| Ion Transfer Tube Temperature (°C) | 325 | 300 | |
| Vaporizer Temperature (°C) | 350 | 400 | |
| S-len RF level | NA | 55 | |
| **SRM^1^ parameters** | | **DDA^2^ parameters** | |
| Cycle Time (second) | 0.3 | FS Resolution (FWHM) | 70,000 |
| Points Per Peak | 20 | FS AGC Target | 1e6 |
| Q1 Resolution (FWHM) | 0.7 | FS Maximum Injection Time (ms) | 55 |
| Q3 Resolution (FWHM) | 1.2 | FS Scan Range (*m/z*) | 300 to 1400 |
| CID Gas (mTorr) | 1.5 | PRM Resolution (FWHM) | 17,500 |
|  |  | PRM AGC Target | 1e5 |
|  |  | PRM Maximum Injection Time (ms) | 55 |
|  |  | TopN | 5 |
|  |  | Isolation Window (m/z) | 3 |
|  |  | Intensity Threshold | 1.5e5 |
|  |  | Exclude Isotopes | On |
|  |  | Dynamic Exclusion (s) | 1 |

1. Selected reaction monitoring (SRM)

2. Data dependent acquisition (DDA)

**Table S3**. Selected reaction monitoring (SRM) parameters for targeted compounds quantitative analysis

| **Compounds** | **Retention Time (min)** | **Retention Time Window (min)** | **RF Lens (V)** | **Precursor ion (*m/z*)** | **Product ion (*m/z*)*** | **Collision Energy (V)** |
| --- | --- | --- | --- | --- | --- | --- |
| AG-98A | 3.52 | 0.25 | 87 | 655.3 | 281.1 | 40 |
|  |  |  |  |  | 575.2 | 18 |
| AG-98B | 4.07 | 0.35 | 89 | 689.2 | 281.2 | 41 |
|  |  |  |  |  | 609.2 | 18 |
| AP-B | 4.70 | 0.25 | 127 | 837.4 | 175.1 | 38 |
|  |  |  |  |  | 201.1 | 42 |
| MG-527-ME | 4.96 | 0.25 | 97 | 542.3 | 102 | 35 |
|  |  |  |  |  | 194 | 23 |
| [Asp^3^]MC-RR | 5.22 | 0.25 | 92 | 512.9 | 445.7 | 20 |
|  |  |  |  |  | 620.2 | 27 |
| MG-690-ME | 5.26 | 0.25 | 103 | 705.3 | 357.1 | 25 |
|  |  |  |  |  | 510.2 | 20 |
| AGU-98A | 5.24 | 0.25 | 94 | 982.4 | 804.4 | 27 |
|  |  |  |  |  | 902.4 | 10 |
| MC-RR | 5.28 | 0.25 | 99 | 519.9 | 135.1 | 31 |
|  |  |  |  |  | 213.1 | 36 |
| MC-RR-^15^N_13_ | 5.28 | 0.25 | 99 | 526.1 | 135.1 | 31 |
| AP-A | 5.44 | 0.25 | 121 | 844.4 | 637.2 | 26 |
|  |  |  |  |  | 663.2 | 25 |
| AP-915 | 5.52 | 0.25 | 131 | 916.4 | 709.4 | 29 |
|  |  |  |  |  | 735.3 | 28 |
| NOD-R | 5.60 | 0.25 | 128 | 825.5 | 135.1 | 59 |
|  |  |  |  |  | 389.2 | 42 |
| OC-Y | 5.64 | 0.25 | 145 | 858.4 | 651.3 | 23 |
|  |  |  |  |  | 1024.5 | 15 |
| CP-1041 | 5.66 | 0.25 | 108 | 1042.5 | 184.2 | 47 |
|  |  |  |  |  | 681.2 | 28 |
| MG-FR1 | 5.68 | 0.25 | 112 | 728.4 | 384.2 | 23 |
|  |  |  |  |  | 664.2 | 32 |
| MC-YR | 5.76 | 0.25 | 120 | 1045.5 | 135 | 71 |
|  |  |  |  |  | 1017.2 | 45 |
| MC-YR-^15^N_10_ | 5.76 | 0.25 | 120 | 1055.6 | 215.1 | 65 |
| MC-HtyR | 5.80 | 0.25 | 120 | 1059.5 | 107.1 | 85 |
|  |  |  |  |  | 135.1 | 70 |
| MG-FR2 | 5.82 | 0.25 | 123 | 742.4 | 182.1 | 43 |
|  |  |  |  |  | 398.2 | 25 |
| [Asp^3^]MC-LR | 5.87 | 0.25 | 126 | 981.5 | 135.1 | 66 |
|  |  |  |  |  | 539.2 | 48 |
| MC-LR | 5.87 | 0.25 | 122 | 995.5 | 135.1 | 70 |
|  |  |  |  |  | 213.1 | 64 |
| MC-LR-^15^N_10_ | 5.87 | 0.25 | 122 | 1005.1 | 215.1 | 65 |
| FA-A | 5.96 | 0.25 | 123 | 867.4 | 637.3 | 28 |
|  |  |  |  |  | 686.2 | 37 |
| MC-HilR | 6.00 | 0.25 | 101 | 1009.5 | 213.1 | 77 |
|  |  |  |  |  | 269.1 | 57 |
| AP-J | 6.04 | 0.25 | 107 | 794.4 | 637.2 | 24 |
|  |  |  |  |  | 663.3 | 24 |
| MC-WR | 6.05 | 0.25 | 116 | 1068.5 | 135.1 | 70 |
|  |  |  |  |  | 213.1 | 64 |
| AP-C | 6.14 | 0.25 | 117 | 808.4 | 419.2 | 37 |
|  |  |  |  |  | 651.3 | 25 |
| MC-LA | 6.86 | 0.25 | 113 | 910.4 | 375.2 | 32 |
|  |  |  |  |  | 776.3 | 19 |
| MC-LA-^15^N_7_ | 6.86 | 0.25 | 131 | 917.5 | 215.1 | 65 |
| MC-LY | 6.97 | 0.25 | 118 | 1002.5 | 494.2 | 26 |
|  |  |  |  |  | 868.4 | 20 |
| MC-LW | 7.46 | 0.25 | 140 | 1025.5 | 517.2 | 27 |
|  |  |  |  |  | 891.4 | 21 |
| MC-LF | 7.60 | 0.25 | 144 | 986.5 | 478.2 | 25 |
|  |  |  |  |  | 852.4 | 20 |

*The first product ion is for quantification, and the second product ion is for confirmation

**Table S4.** Validation parameters of the quantitative method including accuracy and precision.

| **Compounds** | **Accuracy (%)^1^** | |  | **Intra-day precision^2^ (RSD, %)** | |  | **Inter-day precision^3^ (RSD, %)** | |
| --- | --- | --- | --- | --- | --- | --- | --- | --- |
|  | Spike level (µg L^-1^) | |  | Spike level (µg L^-1^) | |  | Spike level (µg L^-1^) | |
|  | *2.5* | *50* |  | *2.5* | *50* |  | *2.5* | *50* |
| AG-98A | 94 ± 5 | 97 ± 3 |  | 2 | 2 |  | 10 | 13 |
| AG-98B | 107 ± 6 | 105 ± 4 |  | 3 | 1 |  | 9 | 12 |
| AGU-98A | 112 ± 10 | 90 ± 5 |  | 5 | 6 |  | 10 | 10 |
| AP-A | 94 ± 1 | 93 ± 3 |  | 1 | 2 |  | 2 | 3 |
| AP-B | 95 ± 2 | 107 ± 2 |  | 7 | 4 |  | 6 | 4 |
| AP-C | 103 ± 11 | 101 ± 3 |  | 5 | 1 |  | 9 | 9 |
| AP-J | 106 ± 3 | 95 ± 3 |  | 4 | 1 |  | 9 | 3 |
| AP-915 | 100 ± 2 | 97 ± 5 |  | 2 | 5 |  | 5 | 7 |
| FA-A | 104 ± 1 | 95 ± 2 |  | 2 | 2 |  | 11 | 4 |
| OC-Y | 110 ± 2 | 103 ± 2 |  | 3 | 1 |  | 8 | 4 |
| CP-1041 | 103 ± 2 | 93 ± 2 |  | 3 | 1 |  | 9 | 10 |
| [Asp^3^]MC-RR | 89 ± 3 | 95 ± 3 |  | 6 | 4 |  | 10 | 8 |
| MC-RR | 109 ± 2 | 94 ± 3 |  | 1 | 1 |  | 12 | 3 |
| MC-YR | 100 ± 1 | 96 ± 3 |  | 5 | 6 |  | 7 | 8 |
| MC-HtyR | 95 ± 4 | 96 ± 4 |  | 2 | 1 |  | 6 | 5 |
| [Asp^3^]MC-LR | 110 ± 5 | 105 ± 2 |  | 8 | 2 |  | 8 | 7 |
| MC-LR | 113 ± 6 | 102 ± 3 |  | 3 | 2 |  | 11 | 4 |
| MC-HilR | 110 ± 3 | 101 ± 1 |  | 4 | 1 |  | 4 | 7 |
| MC-WR | 102 ± 6 | 105 ± 3 |  | 3 | 1 |  | 8 | 5 |
| MC-LA | 104 ± 2 | 100 ± 1 |  | 3 | 1 |  | 7 | 5 |
| MC-LY | 108 ± 6 | 100 ± 1 |  | 8 | 4 |  | 10 | 11 |
| MC-LW | 97 ± 3 | 90 ± 6 |  | 2 | 2 |  | 2 | 3 |
| MC-LF | 101 ± 3 | 93 ± 5 |  | 4 | 1 |  | 3 | 7 |
| MG-527-ME | 103 ± 6 | 91 ± 2 |  | 2 | 1 |  | 3 | 4 |
| MG-690-ME | 107 ± 4 | 91 ± 4 |  | 2 | 1 |  | 14 | 3 |
| MG-FR1 | 96 ± 3 | 94 ± 2 |  | 8 | 8 |  | 4 | 5 |
| MG-FR2 | 105 ± 4 | 93 ± 7 |  | 3 | 5 |  | 5 | 7 |
| NOD-R | 88 ± 7 | 103 ± 1 |  | 2 | 3 |  | 5 | 4 |

1. Recovery value from spiked level.
2. Intra-day precision was determined with five consecutive injections during the same day.
3. Inter-day precision was determined with injections for five consecutive days.

| **Nodes Settings** | **FISh Scoring Experiment** | **Compound Class Experiment** |
| --- | --- | --- |
| **Select Spectra** | | |
| Lower RT Limit | 1 | |
| Uppet RT Limit | 25 | |
| Polarity Mode | Positive | |
| **Detect Compounds** | | |
| Mass Tolerance (ppm) | 5 | |
| Minimum Peak Intensity | 10000 | |
| Use Most Intense Isotope | True | |
| Chromatographic S/N Threshold | 1.5 | |
| Group Isotopes for | Br, Cl | |
| Ions | All options available | |
| **Group Compounds** | | |
| Mass Tolerance (ppm) | 5 | |
| RT Tolerance (min) | 0.1 | |
| Preferred Ions | [M + H]+1; [M+2H]+2; [M+NH4]+1; [M+Na]+1 | |
| **Search Mass Lists** | | |
| Mass Lists | CyanoMetDB 2023 | NA |
| Mass Tolerance (ppm) | 5 | NA |
| **Predict Composition** | | |
| Mass Tolerance | 5 | |
| Minimun Element Counts | C H | |
| Maximum Elements Counts | C90 H190 Br5 Cl4 F5 I N10 O18 P3 S5 Si15 | |
| Minimun RDBE | 0 | |
| Maximum RDBE | 40 | |
| Minimun H/C | 0.1 | |
| Maximum H/C | 3.5 | |
| Maximum # Candidates | 10 | |
| Intensity Tolerance [%] | 30 | |
| Intensity Threshold [%] | 0.1 | |
| S/N Threshold | 3 | |
| Use Dynamic Recalibration | True | |
| Use Fragments Matching | True | |
| **Apply Spectral Distance** | | |
| Mass Tolerance (ppm) | 5 | |
| Intensity Tolerance [%] | 30 | |
| Intensity Threshold [%] | 0.1 | |
| S/N Threshold | 3 | |
| Use Dynamic Recalibration | True | |
| **Compound Class Scoring** | | |
| Compound Classes | NA | One class per target compound |
| S/N Threshold | NA | 3 |
| High Acc. Mass Tolerance | NA | 5 ppm |
| Low Acc. Mass Tolerance | NA | 0.5 Da |
| Use Full MS Tree | NA | True |
| Allow DIA Scoring | NA | True |

**Table S5.** Compound Discoverer 3.3 workflows.

**Table S6.** Calculated Log P of targeted compounds.

| **Compounds** | **RT^*^**  **(min)** | **Log P** |
| --- | --- | --- |
| AG-98A | 7.57 | -0.62 |
| AG-98B | 6.59 | -1.48 |
| AGU-98A | 10.24 | -3.97 |
| AP-A | 10.57 | 0.58 |
| AP-B | 8.73 | -1.47 |
| AP-C | 12.01 | -0.89 |
| AP-J | 11.82 | 0.88 |
| AP-915 | 10.78 | 1.90 |
| FA-A | 11.69 | 1.21 |
| OC-Y | 10.98 | 1.08 |
| CP-1041 | 11.10 | 0.77 |
| [Asp^3^]MC-RR | 9.74 | -4.82 |
| MC-RR | 9.87 | -5.10 |
| MC-YR | 10.94 | -3.96 |
| MC-HtyR | 11.09 | -3.45 |
| [Asp^3^]MC-LR | 11.11 | -2.89 |
| MC-LR | 11.17 | -3.64 |
| MC-HilR | 11.41 | -3.39 |
| MC-WR | 11.54 | -3.33 |
| MC-LA | 13.75 | -2.57 |
| MC-LY | 14.02 | -1.58 |
| MC-LW | 15.08 | -0.95 |
| MC-LF | 15.38 | -1.11 |
| MG-527-ME | 9.21 | 0.88 |
| MG-690-ME | 9.82 | 0.76 |
| MG-FR1 | 10.69 | 1.04 |
| MG-FR2 | 11.10 | 1.95 |
| NOD-R | 10.60 | -3.57 |

^*^ Retention time using suspect screening method


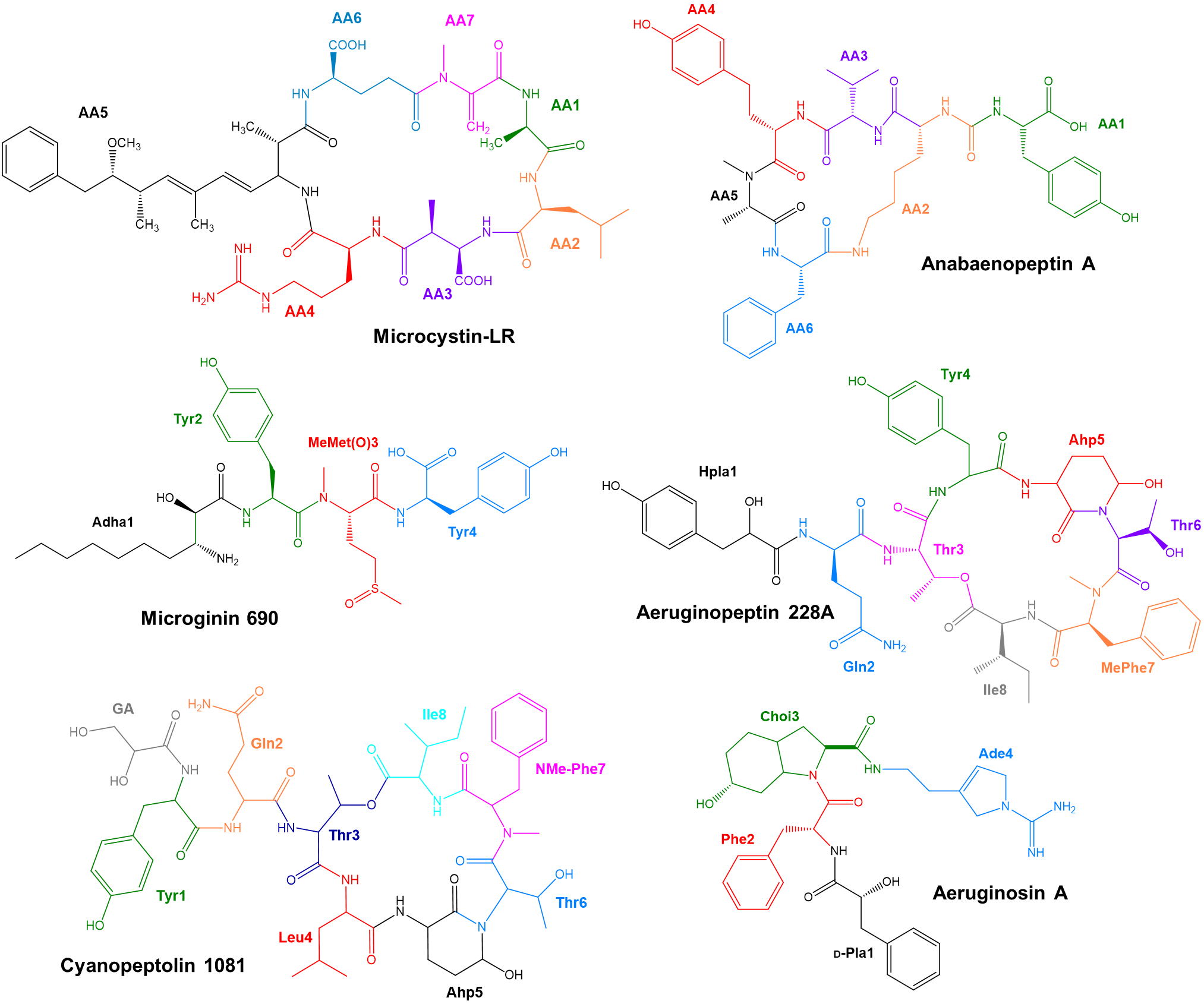


**Figure S3.** Chemical structures of microcystins (MC-LR), anabaenopeptins (AP-A) and other found cyanopeptides : Microginin 690, Aeruginopeptin 228A, Cyanopeptolin 1081 and Aeruginosin A.

**Table S7.** Microcystins (MCs) specific ions from MS^2^ fragmentation

| Fragmentation sequence | [Ser^7^]  MC-LR | [DMAdda^5^,GluOMe^6^]  MC-LHty | [seco-4/5]  [D-Asp^3^]  MC-HtyR | [DAsp^3^]  MC-MR | [DAsp^3^]  MC-M(O)R | [DMAdda^5^]MC-YR | [D-Ser^1^,D-Asp^3^]MC-HtyR | [D-Asp^3^,  DMAdda^5^]  MC-LR | [Mdha-GSH^7^]  MC-LR | [epoxyAdda^5^]MC-LR | [DMAdda^5^]MC-LR | [seco-1/2]  MC-LR |
| --- | --- | --- | --- | --- | --- | --- | --- | --- | --- | --- | --- | --- |
| M+H^+^ | 999.5503 | 1016.5310 | 1063.5370 | 999.4937 | 1015.4898 | 1031.5168 | 1061.5292 | 967.5260 | 1302.6398 | 1011.5501 | 981.5108 | 1013.5672 |
| M+2H^2+^ | 500.2790 |  | 532.2754 | 500.2506 | 508.2494 | 516.2636 | 531.2687 | 484.5656 | 651.8229 | 506.2815 |  |  |
| M+H^+^-H_2_O |  | *998.5264** |  |  |  |  |  |  |  |  |  |  |
| M+H^+^-CO | *971.5533** | *988.5381** |  |  |  | *1003.5235** | *1033.5291** | *939.5304** |  |  | *963.5499** | 985.5735 |
| M+H^+^-134 (Adda) |  | 882.4664 |  | 865.5233 |  |  |  |  | 1168.5671 | 877.4788 |  |  |
| M+H^+^-134 (Adda)-NH_3_ |  |  | *912.4440** |  |  |  |  |  |  |  |  |  |
| AA3+AA4+Adda+H^+^ |  |  |  |  |  | 585.3378 |  |  |  |  | 585.3401 |  |
| AA3+AA4+Adda-CO+H^+^ |  |  |  |  |  |  |  |  |  |  |  | *571.3608** |
| AA4+Adda+AA6+H^+^ | *599.3565** | *620.3315** |  | *599.3580** | 599.3544 | 585.3378 | *599.3585** | *585.3985** | *599.3546** | *615.3487** | 585.3401 | *599.3564** |
| AA4+Adda+AA6-CO+H^+^ |  | *592.3373** |  |  |  |  | *571.3624** | *557.3474** | *571.3599** | *587.3580** |  | *571.3608** |
| AA6+AA7+AA1+AA2+AA3-NH_3_+H^+^ |  | *523.2369** |  |  |  |  |  |  |  |  |  |  |
| AA1+AA2+AA3+AA4+H^+^ |  | *491.2527** | *520.2518** |  |  |  |  |  | 470.2716 |  |  |  |
| AA1+AA2+AA3+AA4-NH_3_+H^+^ |  |  |  |  |  |  | 519.2229*** | 439.2038*** |  |  |  |  |
| AA1+AA2+AA3+AA4-CO-NH_3_+H^+^ |  | *446.2280** | *475.2307** |  | *445.1869** |  |  |  |  |  |  |  |
| Adda-134+AA6+AA7+AA1-NH_3_+H^+^ | *450.2238** | *446.2280** |  |  | *446.2291** |  |  |  | *682.2752** |  |  | *446.2322** |
| AA2+AA3+AA4+H^+^ |  |  | *449.2149** |  |  |  |  |  |  |  |  | *399.2356** |
| Adda-134+AA6+AA7-NH_3_+H^+^ | *379.1879** | *375.1910** | *375.1906** | *375.1895** | *375.1923** | *361.1742** | *375.1928** | *361.1751** |  | *391.1856** | *361.1741** | *375.1945** |
| Adda-134+AA6+AA7-NH_3_-CO+H^+^ |  |  |  |  |  |  |  |  |  |  |  | *347.1971** |
| AA2+AA3+H^+^ |  |  |  |  | *263.0702** |  |  |  |  |  |  |  |
| AA6+AA7+H^+^ | 217.0822 | 227.1028 | 213.0872 | 213.0869 | 213.0885 | 213.0869 | 213.0864 | 213.0880 | *520.1696** | 213.0871 | 213.0873 | 213.0870 |
| [AA4+NH_2_+2H]^+^ | 174.1350 | *195.2800** | *174.1349** | *174.1352** | *174.1345** | *174.1347** | *174.1348** | *174.1348** | *174.1348** | *174.1349** | *174.1354** | *174.1354** |
| AA7+AA1+H^+^ | *159.0762** | 155.0817 | *155.0826** | *155.0815** | 155.0822 | 155.0818 | 171.0771 | *155.0801** | *462.1646** | 155.0813 | 155.0823 | 155.0821 |
| Adda frag (Ph-CH_2_-CH(O^+^Me) | 135.0806 | 135.0806 | 135.0803 | 135.0805 | 135.0805 | 135.0807 | 135.0805 | 135.0805 | 135.0804 | 135.0804 | 135.0806 | 135.0804 |
| AA7+AA1-CO+H^+^ | *131.0820** | 127.0868 | 127.0865 | *127.0867** | *127.0870** | 127.0869 | 143.0824 | *127.0871** | *434.1701** |  | 127.0872 | 127.0869 |
| AA7+H^+^ |  |  |  |  |  |  |  |  | *391.1294** |  |  |  |
| AA2 Immonium | 86.0971 | 86.0971 | 150.0910 | *104.0531** | *120.0459** | *136.0836** | *150.0912* | 86.0971 | *86.0969** | 86.0970 | 86.0969 | 86.0969 |

*Fragments in *italic* were not selected in the FISh scoring experiment.


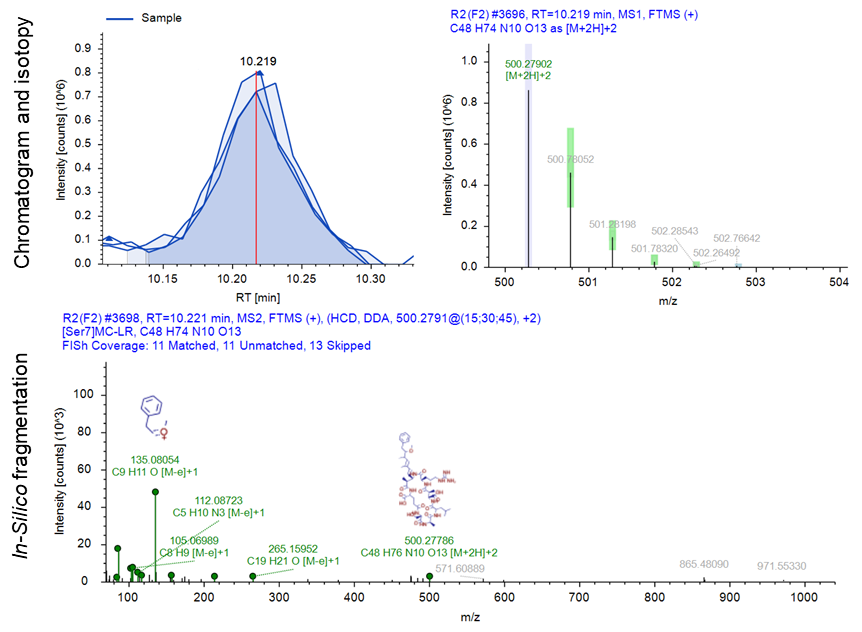


**C**

**B**

**A**

**Figure S4.** Structure characterization of [Ser^7^]microcystin-LR. A) Extracted ion chromatogram of ion *m/z* 999.5503 and B) isotopic pattern of most intense precursor ion. C) Fragmentation spectrum and *in-silico* matching with FISh coverage.

Amino acid Ser is confirmed at position 7 with ions: *m/z* 450.2238, 379.1879, 217.0822, 159.0762, and 131.0820. Additionally, the absence of a thiol derivative supported the absence of Mdha at position 7. The spectra also retained microcystin-LR characteristics, particularly with *m/z* 599.3565 confirming Arg at position 4 and *m/z* 86.0971 as the Leu immonium ion at position 2.

**SI-2.** Spectral interpretation of [DMAdda^5^, GluOMe^6^]microcystin-LHty

Spectra (Figure 3 in the main text) showed an ion at *m/z* 227.1028 indicating GluOMe at position 6 and Mdha at position 7, along with a thiol derivative at *m/z* 1094.5449. Also, the ion at *m/z* 195.2800 confirmed Hty at position 4 and *m/z* 86.0971 the Leu immonium ion at position 2. Furthermore, ions at *m/z* 620.3315, 592.3372, 446.2280, and 375.1910 confirmed DMAdda at position 5.


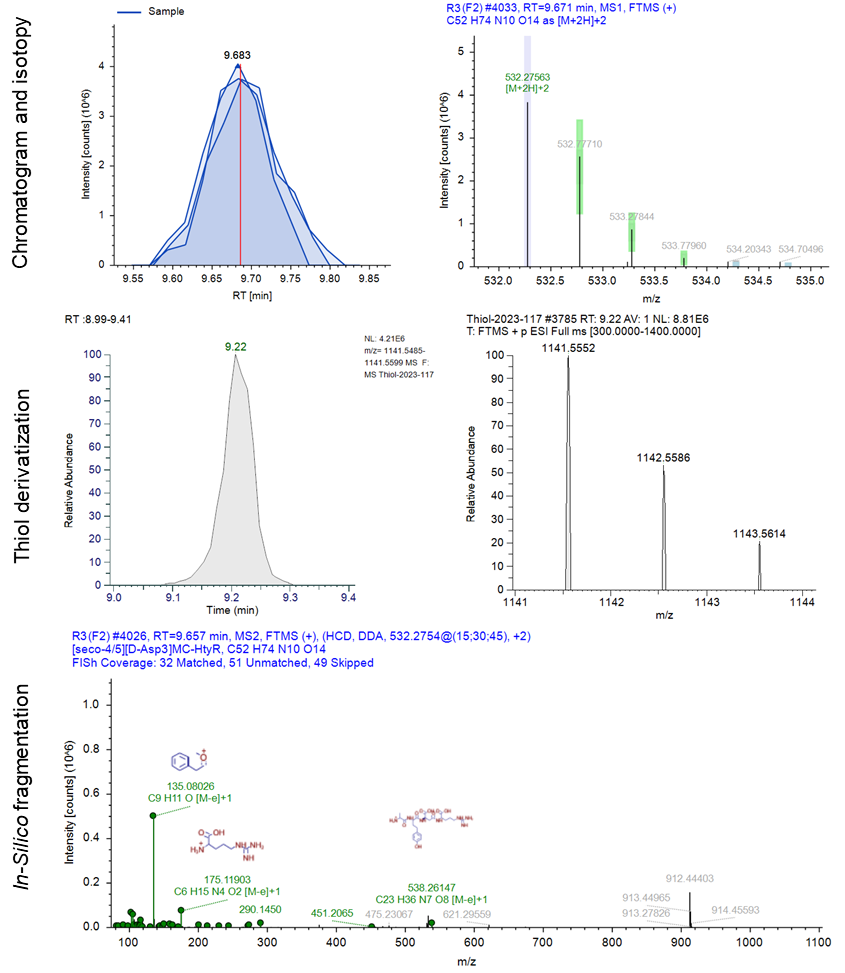


**E**

**C**

**D**

**A**

**B**

**Figure S5.** Structure characterization of [seco-4/5][D-Asp^3^]microcystin-HtyR. A) Extracted ion chromatogram of ion *m/z* 1063.5370 and B) isotopic pattern of most intense precursor ion. C) Extracted ion chromatogram of thiol derivative ion *m/z* 1141.5552 and D) isotopic pattern of thiol derivative. E) Fragmentation spectrum and *in-silico* matching with FISh coverage.

The presence of Arg at position 4 was confirmed by *m/z* 174.1349, Leu at position 2 by *m/z* 150.0910, and D-Asp at position 3 by *m/z* 449.2149. Positions 1, 6, and 7 were confirmed by ions *m/z* 375.1906, 213.0872, 155.0826, and 127.0865, along with a thiol derivative at *m/z* 1141.5552 confirming Mdha at position 7. Absence of ions *m/z* 599.3557 supports the cycle cleavage between positions 4 and 5, confirming the linear structure of this microcystin.


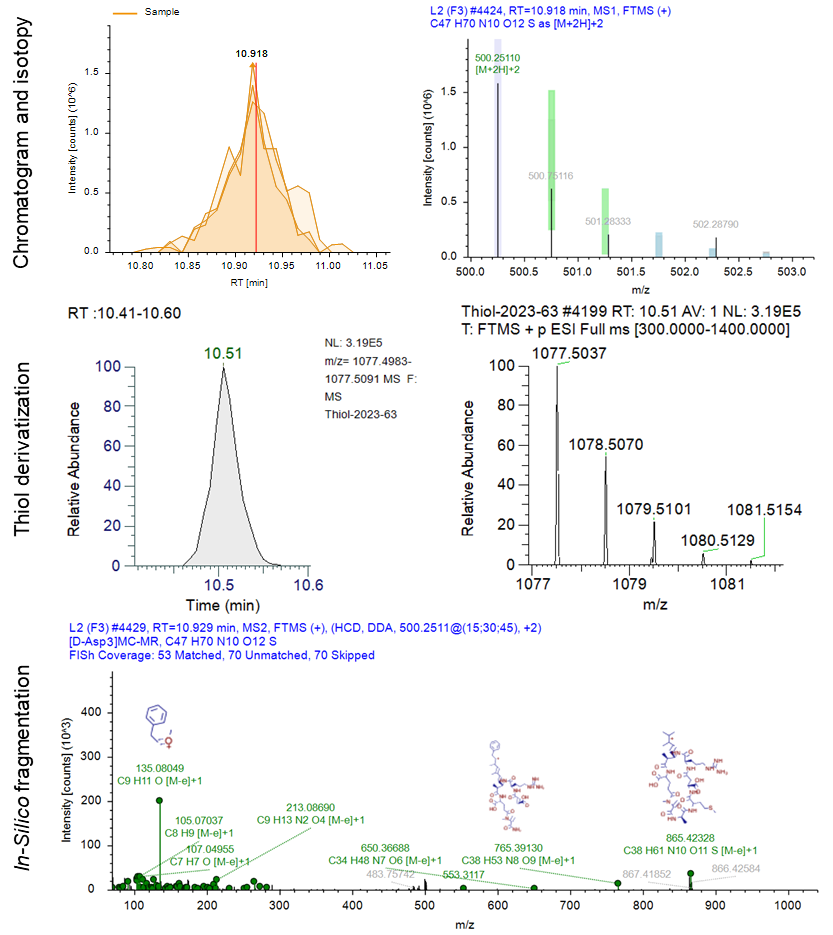


**E**

**D**

**B**

**A**

**C**

**Figure S6.** Structure characterization of [D-Asp^3^]microcystin-MR. A) Extracted ion chromatogram of ion *m/z* 999.4937 and B) isotopic pattern of most intense precursor ion. C) Extracted ion chromatogram of thiol derivative ion *m/z* 1077.5037 and D) isotopic pattern of thiol derivative. E) Fragmentation spectrum and *in-silico* matching with FISh coverage.

Ion *m/z* 599 confirmed the Arg^4^-Adda^5^-Glu^6^ sequence in the structure. Additionally, ions *m/z* 375, 213, 155, and 127 verified the presence of Ala at position 1 and Mdha at position 7. This was further validated by thiol derivatization, yielding ion *m/z* 1077.5037.


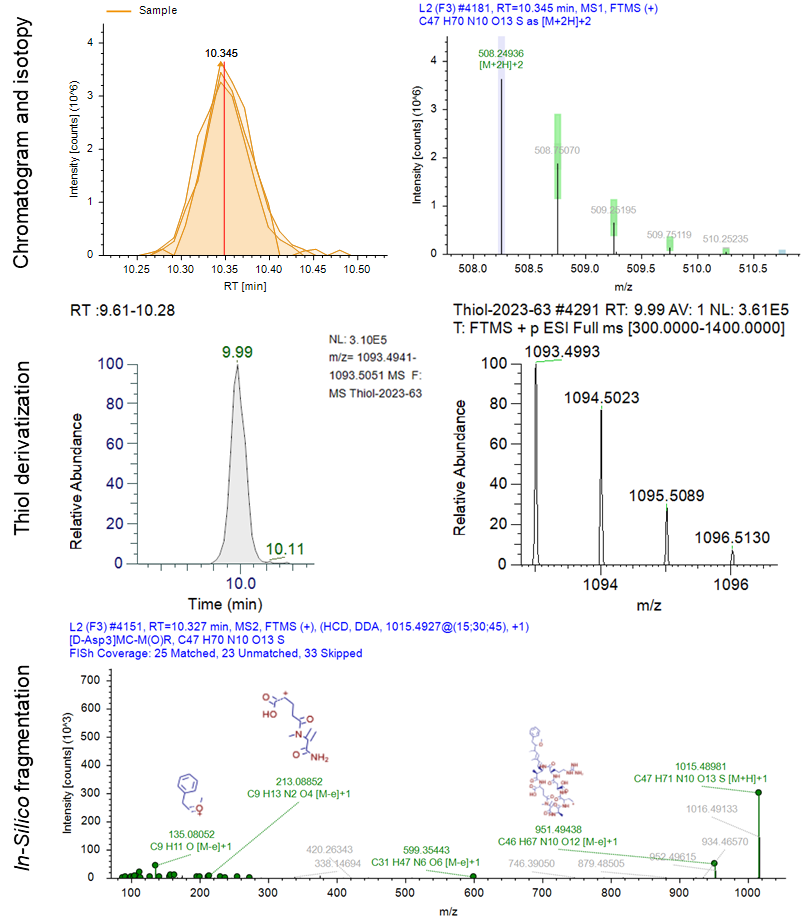


**E**

**D**

**C**

**B**

**A**

**Figure S7.** Structure characterization of [D-Asp^3^]microcystin-M(O)R. A) Extracted ion chromatogram of ion *m/z* 1015.4898 and B) isotopic pattern of most intense precursor ion. C) Extracted ion chromatogram of thiol derivative ion *m/z* 1093.4993 and D) isotopic pattern of thiol derivative. E) Fragmentation spectrum and *in-silico* matching with FISh coverage.

Ion *m/z* 599 confirmed the Arg^4^-Adda^5^-Glu^6^ sequence in the structure. Similar to [D-Asp^3^]microcystin-MR, ions *m/z* 375, 213, 155, and 127 confirmed Ala at position 1 and Mdha at position 7, as validated by thiol derivatization with ion *m/z* 1093.4993 for [D-Asp^3^]microcystin-M(O)R. Additional ions *m/z* 445.1869, 446.2291, 263.0702, and the immonium ion at *m/z* 120.0459 further confirmed the presence of D-Asp at position 3 and M(O) at position 2.


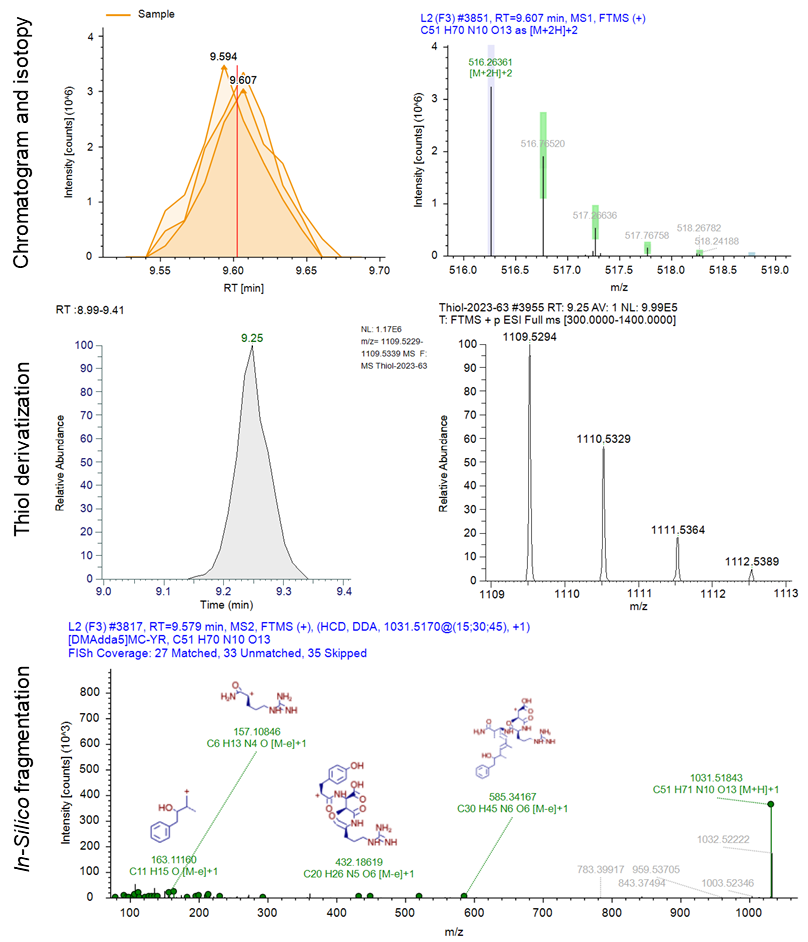


**E**

**D**

**C**

**B**

**A**

**Figure S8.** Structure characterization of [DMAdda^5^]microcystin-YR. A) Extracted ion chromatogram of ion *m/z* 1031.5168 and B) isotopic pattern of most intense precursor ion. C) Extracted ion chromatogram of thiol derivative ion *m/z* 1109.5294 and D) isotopic pattern of thiol derivative. E) Fragmentation spectrum and *in-silico* matching with FISh coverage.

The spectra of [DMAdda^5^]microcystin-YR are similar to microcystin-YR, except for ions *m/z* 585.3378 and 361.1742, demethylated forms of *m/z* 599 ions for Adda and Arg at position 4, and *m/z* 375 present in common microcystins (Adda5-Glu6-Mdha7). Finally, a thiol-derivatized ion confirmed Mdha at position 7.


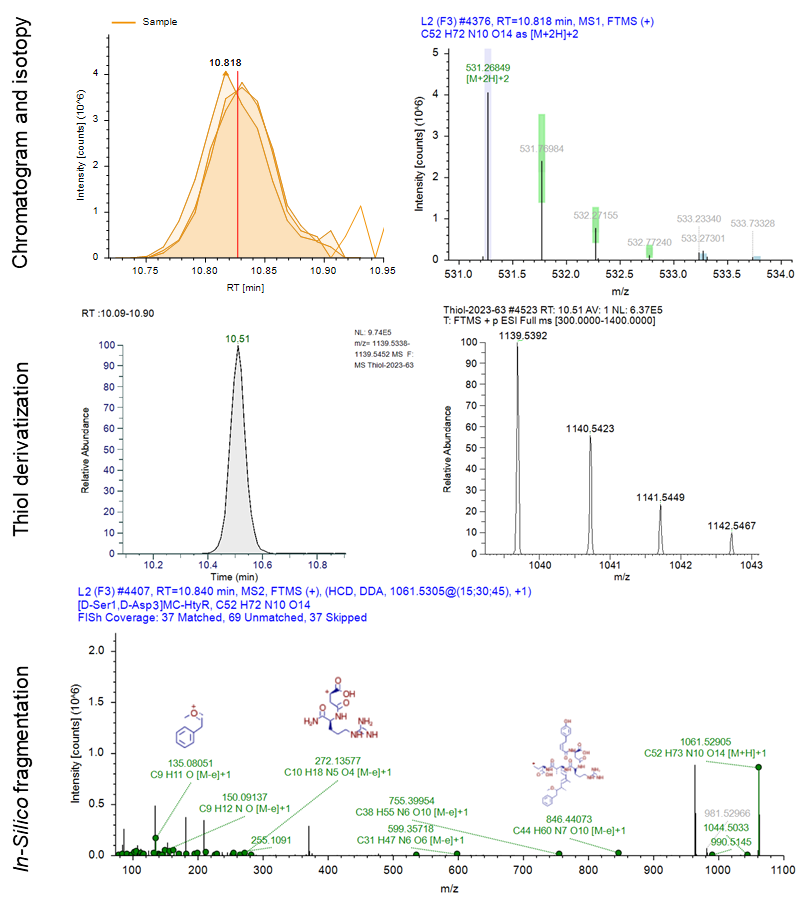


**E**

**C**

**D**

**B**

**A**

**Figure S9.** Structure characterization of [D-Ser^1^, D-Asp^3^]microcystin-HtyR. A) Extracted ion chromatogram of ion *m/z* 1061.5292 and B) isotopic pattern of most intense precursor ion. C) Extracted ion chromatogram of thiol derivative ion *m/z* 1139.5392 and D) isotopic pattern of thiol derivative. E) Fragmentation spectrum and *in-silico* matching with FISh coverage.

Ser is confirmed at position 1 and D-Asp at position 3 with ions: *m/z* 519.2229, 171.0771, and 143.0824. A thiol derivative was found at *m/z* 1139.5392 confirming Mdha at position 7. The spectra retained microcystin-HtyR characteristics, particularly with *m/z* 599.3565 confirming Arg at 4 position and *m/z* 150.0912 as the Hty immonium ion at position 2.


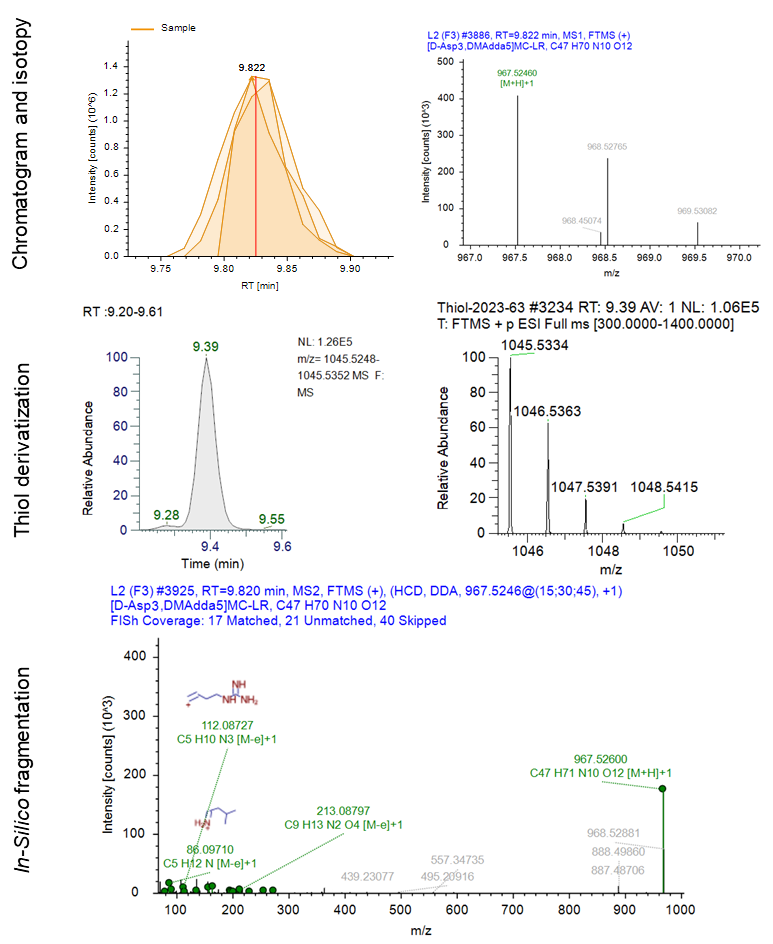


**E**

**D**

**C**

**A**

**B**

**Figure S10.** Structure characterization of [D-Asp^3^, DMAdda^5^]microcystin-LR. A) Extracted ion chromatogram of ion *m/z* 967.5246 and B) isotopic pattern of most intense precursor ion. C) Extracted ion chromatogram of thiol derivative ion *m/z* 1045.5334 and D) isotopic pattern of thiol derivative. E) Fragmentation spectrum and *in-silico* matching with FISh coverage.

D-Asp was confirmed at position 3, DMAdda at position 5, and Arg at position 4 with ions: *m/z* 585.3985, 439.2038, and 361.1751, and a thiol derivative at *m/z* 1045.5334 confirmed Mdha at position 7. Spectra matched microcystin-LR, particularly with *m/z* 213.0880, 174.1348, 155.0801, and 127.0871 confirming positions 1, 6, and 7, and *m/z* 86.0971 as the Leu immonium ion at position 2.


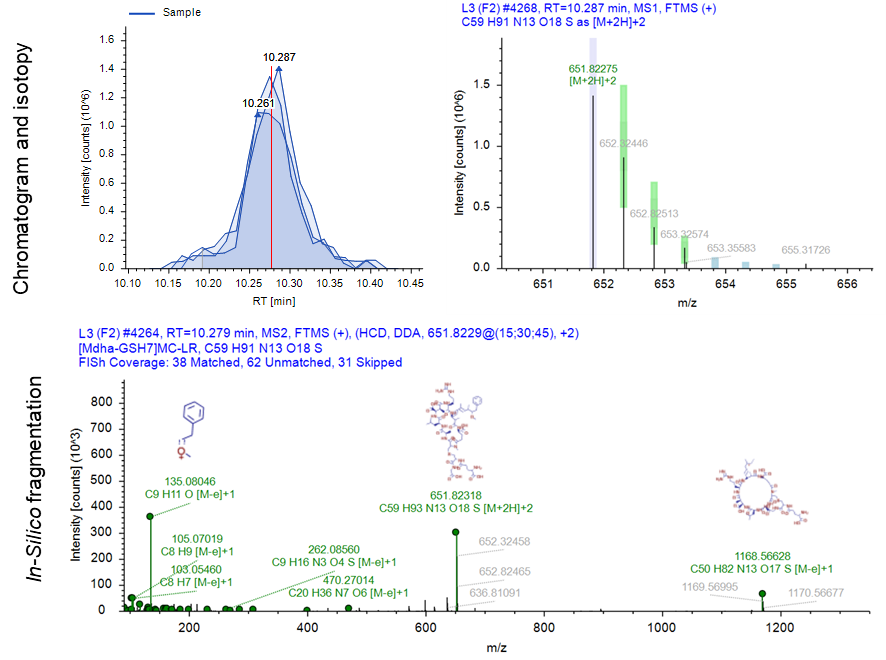


**C**

**B**

**A**

**Figure S11.** Structure characterization of [Mdha-GSH^7^]microcystin-LR. A) Extracted ion chromatogram of ion *m/z* 1302.6398 and B) isotopic pattern of most intense precursor ion. C) Fragmentation spectrum and *in-silico* matching with FISh coverage.

The addition of glutathione (GSH) is validated by observed fragments: *m/z* 682.2752, 520.1696, 462.1646, 434.1701, and 391.1294, also confirming positions 1, 5, and 6. Spectra characteristics also resemble microcystin-LR, particularly with *m/z* 599.3546 and 174.1348 confirming Arg at position 4 and *m/z* 86.0969 as the Leu immonium ion at position 2.


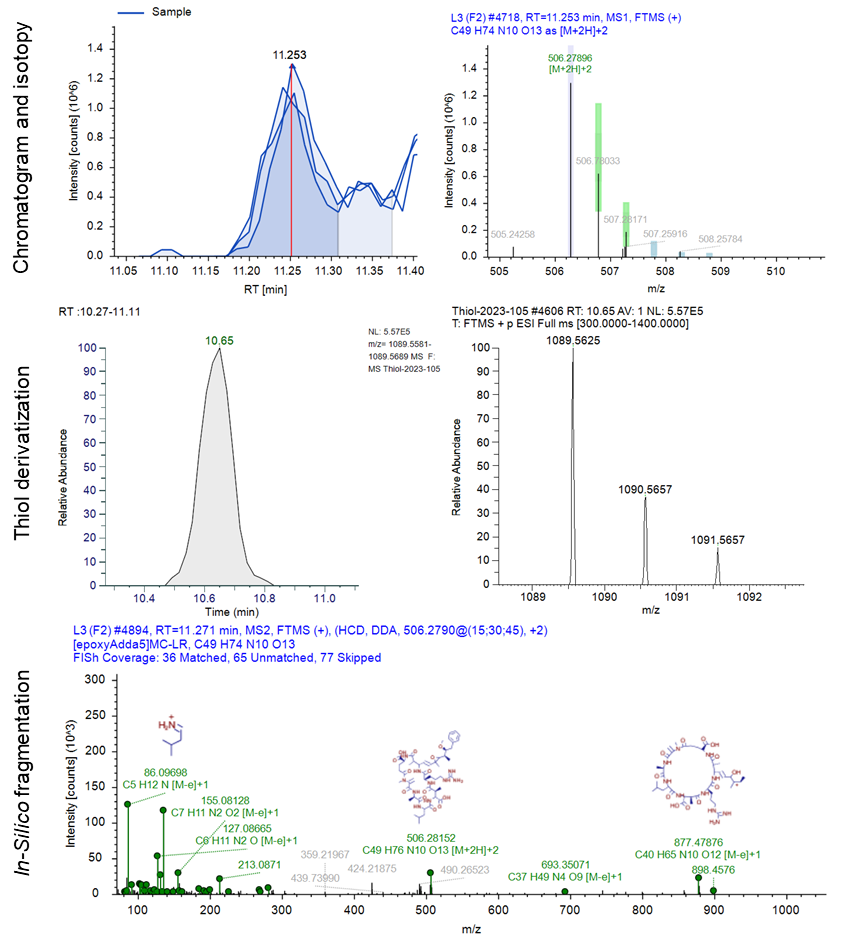


**E**

**D**

**C**

**B**

**A**

**Figure S12.** Structure characterization of [epoxyAdda^5^]microcystin-LR. A) Extracted ion chromatogram of ion *m/z* 1011.5501 and B) isotopic pattern of most intense precursor ion. C) Extracted ion chromatogram of thiol derivative ion *m/z* 1089.5625 and D) isotopic pattern of thiol derivative. E) Fragmentation spectrum and *in-silico* matching with FISh coverage.

Spectra showed *m/z* 615.3487, 391.1856, and 155.0813 confirming Ala at position 1, epoxyAdda at position 5, and Arg at position 4. Furthermore, Mdha at position 7 was validated by the thiol derivative observed at *m/z* 1089.5625. The spectra showed significant similarities to microcystin-LR including *m/z* 213.0871 and 174.1349 validating positions 4 and 6, and 86.0970 corresponding to the Leu immonium ion at position 2.


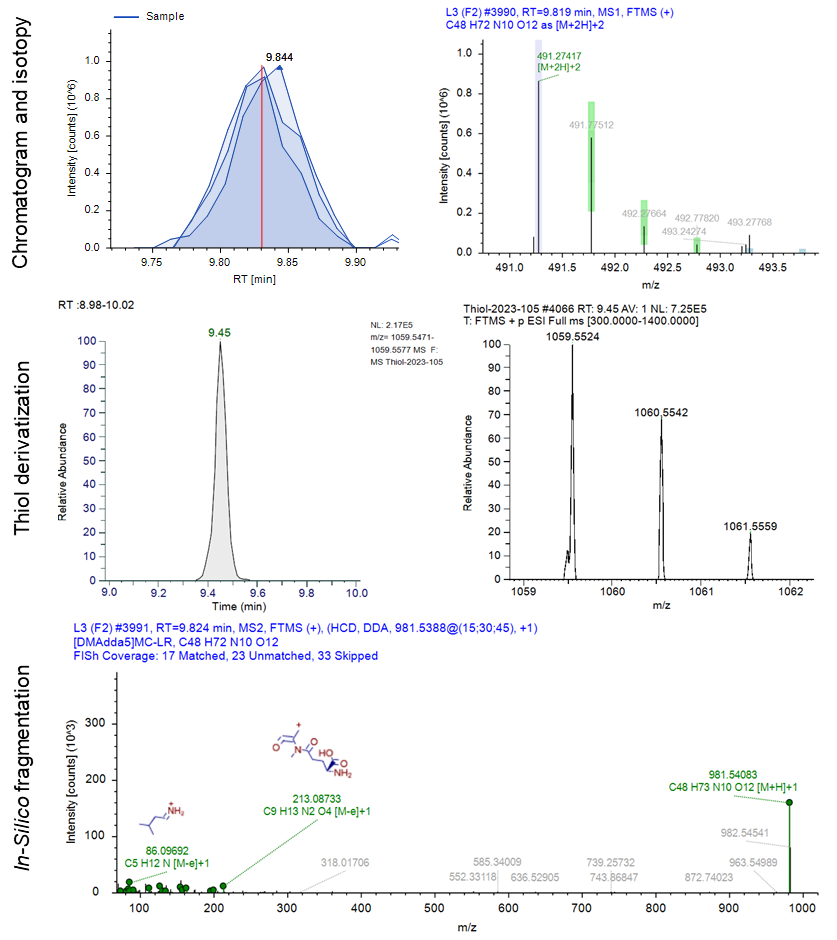


**E**

**D**

**C**

**B**

**A**

**Figure S13.** Structure characterization of [DMAdda^5^]microcystin-LR. A) Extracted ion chromatogram of ion *m/z* 981.5108 and B) isotopic pattern of most intense precursor ion. C) Extracted ion chromatogram of thiol derivative ion *m/z* 1059.5524 and D) isotopic pattern of thiol derivative. E) Fragmentation spectrum and *in-silico* matching with FISh coverage.

The presence of DMAdda at position 5 is showcased by *m/z* 585.3401 and 361.1741. A thiol derivative was detected at *m/z* 1059.5524, confirming Mdha at position 7. The candidate showed strong similarities to microcystin-LR, particularly with ions *m/z* 213.0873, 174.1354, 155.0823, and 127.0872, corroborating positions 1, 4, 6, and 7. Furthermore, Leu at position 2 was confirmed by the immonium ion *m/z* 86.0969.


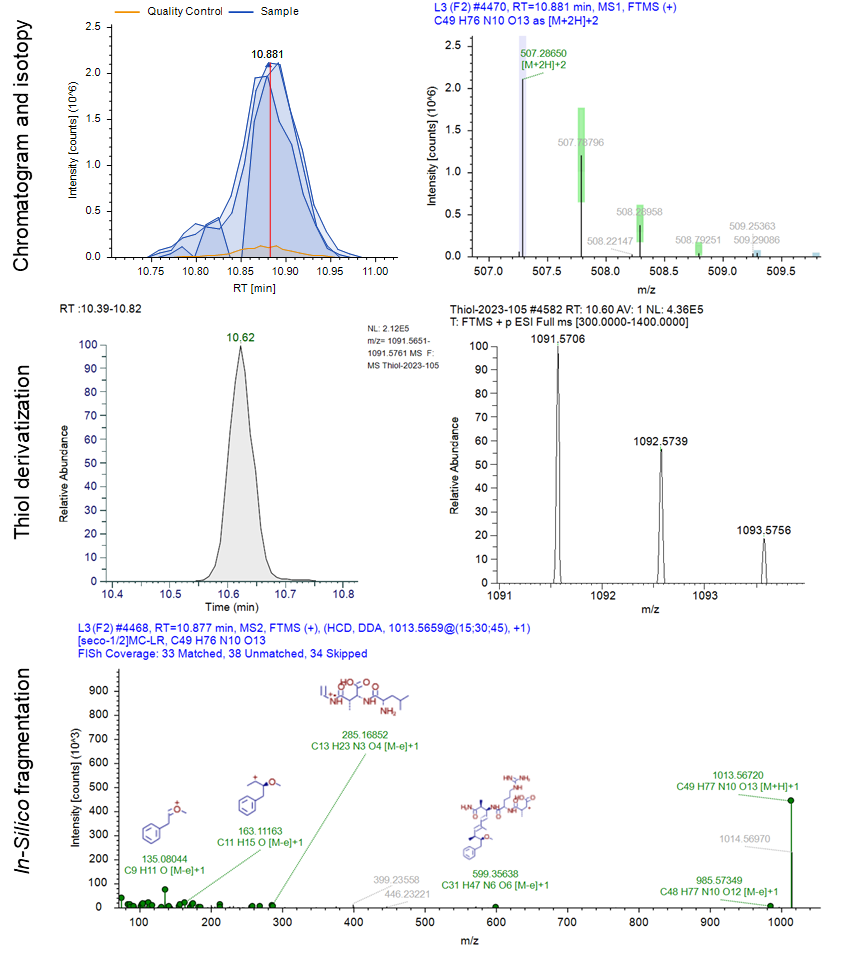


**E**

**C**

**D**

**C**

**B**

**A**

**Figure S14.** Structure characterization of [seco-1/2]microcystin-LR. A) Extracted ion chromatogram of ion *m/z* 1013.5672 and B) isotopic pattern of most intense precursor ion. C) Extracted ion chromatogram of thiol derivative ion *m/z* 1091.5706 and D) isotopic pattern of thiol derivative. E) Fragmentation spectrum and *in-silico* matching with FISh coverage.

Key fragments, such as *m/z* 599.3595 (Arg^4^-Adda^5^-Glu^6^), *m/z* 446.2322 (Adda^5^-Glu^6^-Mdha^7^-Ala^1^), *m/z* 399.2356 (Leu^2^-MeAsp^3^-Arg^4^), and *m/z* 347.19708 (Adda^5^-Glu^6^-Mdha^7^), validate the connectivity of the microcystin, while the parent mass *m/z* 1013.5672 supports its identification as a linear form of microcystin-LR. Additionally, a thiol derivative was detected at *m/z* 1091.5706, confirming Mdha at position 7.

**Table S8.** Anabaenopeptins (APs) specific ions from MS^2^ fragmentation (with associated sample).

| Fragmentation sequence | AP-F (7) | AP-E (7) | AP-H | AP-HU892 | AP-SA3 (10) | AP-679 (10) | AP-F (11) | AP-E (11) | AP-SA3 (11) | AP-679 (11) |
| --- | --- | --- | --- | --- | --- | --- | --- | --- | --- | --- |
| M+H^+^ | 851.4758 | 851.4763 | 923.5311 | 893.5226 | 823.4698 | 680.3766 | 851.4767 | 851.4768 | 823.4705 | 680.3759 |
| M+Na^+^ |  |  |  |  |  |  |  |  |  | 702.3579 |
| M+2H^2+^ | 426.2414 | 426.2419 | 462.2695 | 447.2647 | 412.2386 |  | 426.2421 | 426.2427 | 412.2389 | 340.6915 |
| M+H^+^-CO-AA1-O | *651.3861** | *651.3835** | *723.4321** | 693.4332 | *651.3855** | NF | 651.3856 | 651.3870 | *651.3856** | NF |
| M+H^+^-AA4-AA5 |  |  |  | *541.3425** |  |  |  |  |  |  |
| M+H^+^-AA3-AA4-CO |  |  | *605.3735** |  |  |  |  |  |  |  |
| AA6+Lys^2^+CO+AA3+H^+^ |  |  |  |  | *403.2317** | 403.2357 |  |  | 403.2338 | *403.2353** |
| AA3+AA4+AA5+H^+^ | 376.2222 | *376.2229** |  |  | *362.2087** | 362.2072 | *376.2230** | 376.2261 | *362.2063** | 362.2067 |
| AA4+AA5+H^+^ | 263.1393 | 277.1540 | 369.1792 | *353.1843** | 263.1386 | 263.1386 | 263.1391 | *277.1548** | 263.1391 | 263.1387 |
| Lys+AA3+AA6+CO-NH_3_+H^+^ |  |  | *366.2979** |  |  |  |  |  |  |  |
| AA5+AA6+H^+^ | *213.1292** | *233.1296** | *305.1848** | 305.1845 | 233.1280 | 233.1281 | *213.1290** | *233.1289** | 233.1278 | 233.1281 |
| AA3+AA4+H^+^ |  |  | *291.1689** | *261.1589** |  |  |  |  |  | 277.1558 |
| AA1 Ureido Bridge | 201.0980 | 201.0979 | 201.0977 | 201.0978 | *173.0373** | NF | 201.0981 | 201.0981 | *173.0924** | NF |
| AA1 Sidechain | 175.1188 | 175.1187 | 175.1187 | 175.1187 | *147.1131** | NF | 175.1189 | 175.1189 | 147.1128 | NF |
| AA1+H_2_O-NH_3_+H^+^ | 158.0922 | 158.0920 | 158.0923 | 158.0914 | 130.0861 | NF | 158.0925 | 158.0920 | 130.0868 | NF |
| Lys Residue Chain | 129.1020 | 129.1015 | 129.1029 | 129.1016 | 129.1012 | NF | 129.1021 | 129.1019 | 129.1019 | NF |
| Ph-CH_2_-OH | 107.0492 | NF | 107.0493 | 107.0491 | 107.0495 | 107.0493 | *107.0495** | NF | 107.0495 | 107.0497 |
| Lys Immonium | 84.0813 | *84.0813** | 84.0812 | 84.0811 | *84.0813** | 84.0812 | 84.0812 | *84.0813** | 84.0813 | 84.0814 |

*Fragments in *italic* were not selected in the FISh scoring experiment.
NF: Not found – Absence of a characteristic fragment confirming a congener structure compared to an analogue (AP-E and F; AP-SA3 and AP-679).


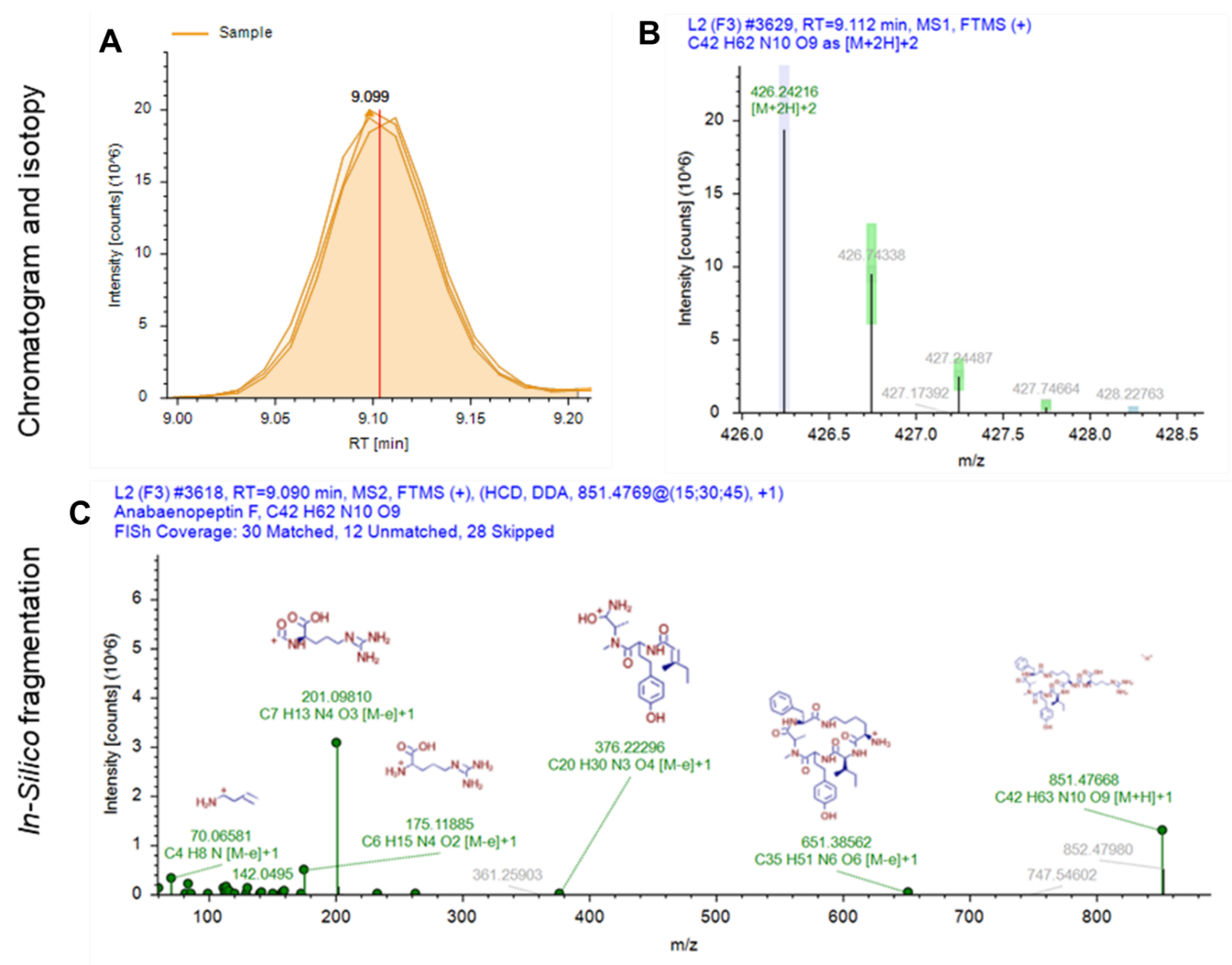


**Figure S15.** Structure characterization of anabaenopeptin F. A) Extracted ion chromatogram of ion *m/z* 851.4758 and B) isotopic pattern of most intense precursor ion. E) Fragmentation spectrum and *in-silico* matching with FISh coverage.

**SI-3.** Spectral interpretation of anabaenopeptin-F

The spectra for anabaenopeptin F (Figure 4) reveal ions at *m/z* 376.2230, 263.1391, and 213.1290, confirming the configuration Ile3-Hty4-MeAla5-Phe6, with the presence of *m/z* 201.0981 confirming the Arg-Uerido bridge at position 1.


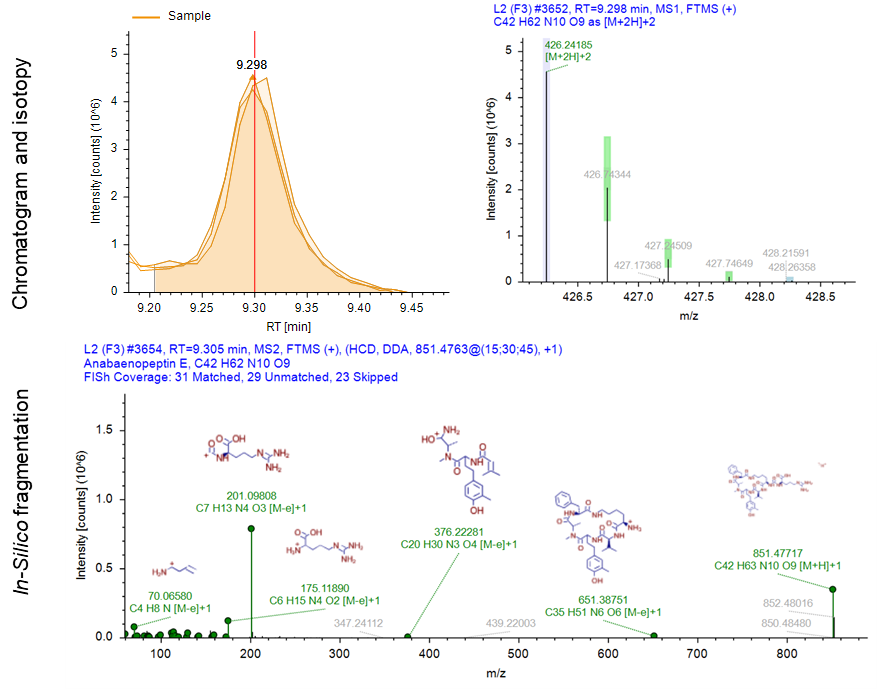


**C**

**B**

**A**

**Figure S16.** Structure characterization of anabaenopeptin E. A) Extracted ion chromatogram of ion *m/z* 851.4755 and B) isotopic pattern of most intense precursor ion. E) Fragmentation spectrum and *in-silico* matching with FISh coverage.

In contrast with anabaenopeptin F, the key fragments are *m/z* 277.1548 and 233.1289 indicate the configuration Val3-MeHty4-MeAla5.

**B**


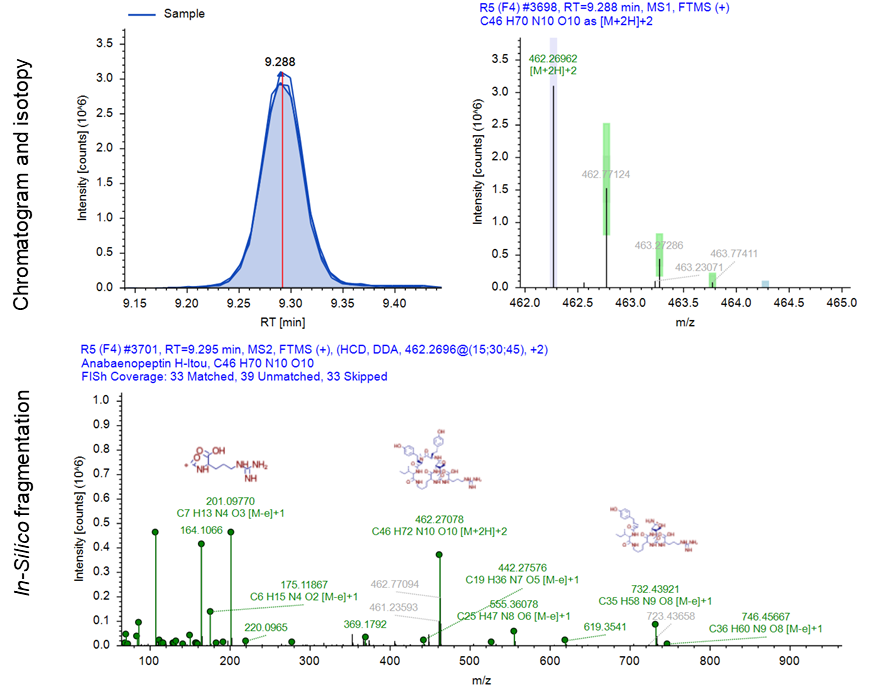


**A**

**C**

**Figure S17.** Structure characterization of anabaenopeptin H. A) Extracted ion chromatogram of ion *m/z* 923.5311 and B) isotopic pattern of most intense precursor ion. E) Fragmentation spectrum and *in-silico* matching with FISh coverage.

Ions at *m/z* 605.3735, 366.2979 and 291.1689 confirm of the presence of Ile at position 3. Additionally, *m/z* 369.1792 and 305.18484 confirmed Hty4-MeHty5-Ile6 in the structure and ion *m/z* 201.0977 validated the presence of Arg-uerido bridge at position 1.


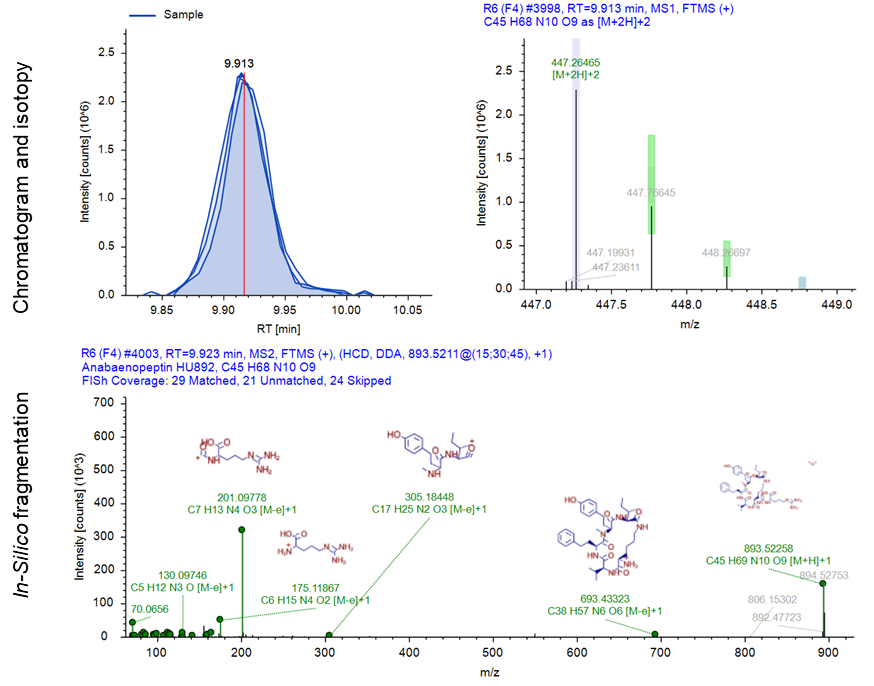


**C**

**B**

**A**

**Figure S18.** Structure characterization of anabaenopeptin HU892. A) Extracted ion chromatogram of ion *m/z* 893.5226 and B) isotopic pattern of most intense precursor ion. E) Fragmentation spectrum and *in-silico* matching with FISh coverage.

Ions at *m/z* 605.3735, 291.1689, 369.1792, and 305.1848 provided confirmation of the presence of positions 3 through 6 within the molecule. Furthermore, the ion at *m/z* 173.0373 indicated the presence of the Arg side chain at position 1.


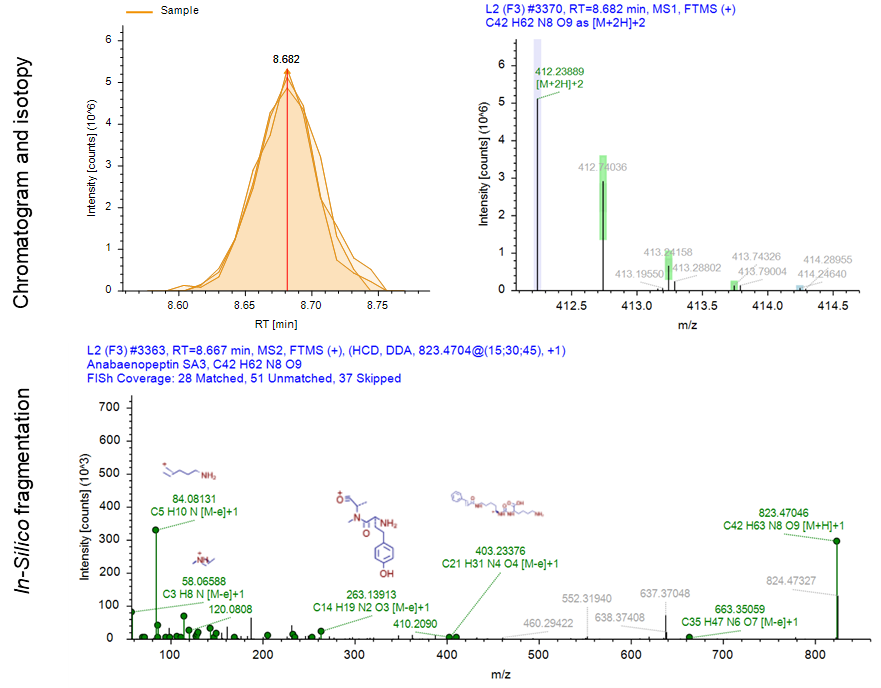


**C**

**B**

**A**

**Figure S19.** Structure characterization of anabaenopeptin SA3. A) Extracted ion chromatogram of ion *m/z* 823.4703 and B) isotopic pattern of most intense precursor ion. E) Fragmentation spectrum and *in-silico* matching with FISh coverage.

The potential structure is Lys1-CO-Lys2-Val3-Hty4-MeAla5-Phe6 with key ions at *m/z* 403.2338, 362.2063, 263.1391, and 233.1278 confirming positions 3 to 6 within the molecule. Additionally, and ion *m/z* 173.0373 is identified as the Arg-ureido bridge confirming position 1.


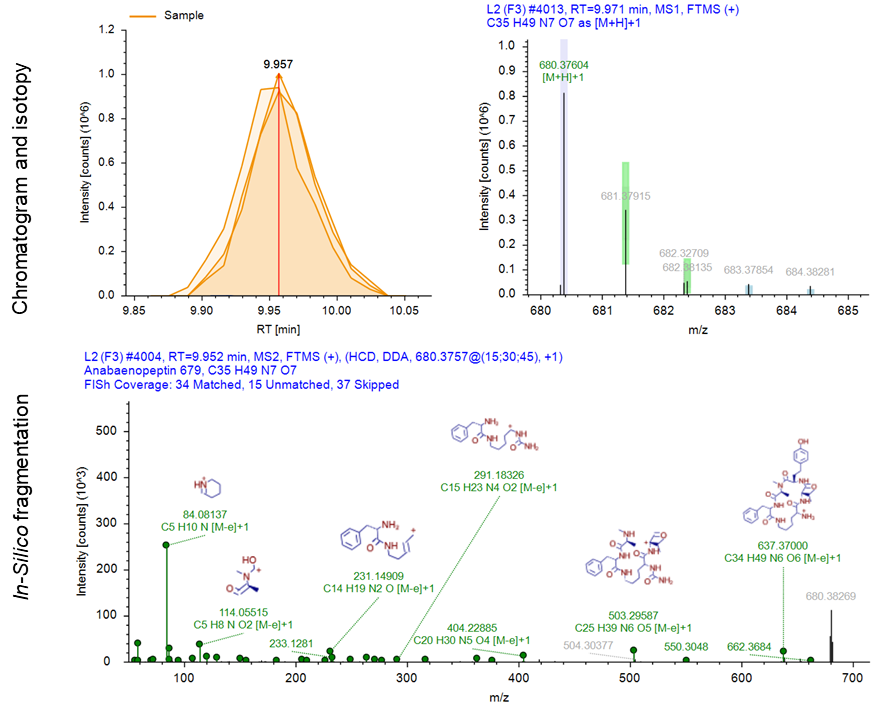


**C**

**B**

**A**

**Figure S20.** Structure characterization of anabaenopeptin 679. A) Extracted ion chromatogram of ion *m/z* 637.3700 and B) isotopic pattern of most intense precursor ion. E) Fragmentation spectrum and *in-silico* matching with FISh coverage.

Compared to anabaenopeptin SA3, the same ions were identified, including the additional *m/z* 277.1558 once again confirming positions 3 to 6. However, the absence of several ions such as 651, 173, 147, 130, and 129, corresponding to fragments containing the Lys-CO side chain at position 1 suggest its absence in the structure. Nonetheless, the consistent presence of the Lys immonium ion at *m/z* 84 in the spectra serves as an indicator of its permanent presence at position 2 in the structure.

**Table S9.** Other cyanopeptides specific ions from MS^2^ fragmentation

| Fragmentation sequence | Microginin 690 |
| --- | --- |
| M+Na^+^ | 713.3183 |
| M+H^+^ | 691.3350 |
| M+2H^+^ | 346.1713 |
| M+H^+^-Tyr4-H_2_O | 510.2621 |
| Adha^1^+Tyr^2^+H_2_O+C_2_O | 349.2127 |
| Tyr^2^+MeMet(O)^3^+H_2_O+H^+^ and MeMet(O)^3^+Tyr^4^+H_2_O+H^+^ | 343.1313 |
| Tyr^2^+MeMet(O)^3^+H^+^ and MeMet(O)^3^-Tyr^4^+H^+^ | 325.1210 |
| Tyr^2^-MeMet(O)^3^-CO+H+ and MeMet(O)^3^-Tyr^4^-CO+H^+^ | 297.1268 |
| Tyr Immonium | 136.0762 |
| Adha Residue | 128.1435 |
| Fragmentation sequence | Aeruginopeptin 228A |
| M+H^+^ | 1045.4854 |
| M+H^+^-H_2_O | 1027.4719 |
| M+H^+^-AA8-H_2_O | 914.3898 |
| M+H^+^-AA8-2H_2_O | 896.3803 |
| Hpla1+Gln2+Thr3+Ahp4-O+H^+^ | 511.2190 |
| Thr^3^+Tyr^4^+H^+^ | 265.1175 |
| Thr^3^+Tyr^4^-H_2_O+H^+^ | 247.1077 |
| Thr^3^+Tyr^4^-H_2_O-CO+H^+^ | *219.1124** |
| Tyr^4^ Immonium | 136.0753 |
| MePhe^8^ Immonium | 134.0961 |
| Gln^2^ Immonium | 101.0713 |
| Ile^9^ Immonium | 86.0967 |
| Thr^3^ Immonium | *74.0606** |
| Thr^6^ Immonium | *74.0606** |
| Fragmentation sequence | Cyanopeptolin 1081 |
| M+Na^+^ | 1104.5212 |
| M+H^+^ | 1082.5393 |
| M+H^+^-H_2_O | 1064.5288 |
| Thr^3^+Leu^4^+Ahp^5^+Thr^6^+NMePhe^7^+Ile^8^-H_2_O+H^+^ | 685.3914 |
| Leu^4^+Ahp^5^+ Thr^6^+NMePhe^7^+Ile^8^+H^+^ | 602.3544 |
| Tyr^1^+GA+Gln^2^+Thr^3^+Leu^4^-H_2_O+H^+^ | 576.2661 |
| Ahp^5^+ Thr^6^+NMePhe^7^+Ile^8^-H_2_O+H^+^ | 471.2606 |
| Tyr^1^+GA+Gln^2^+Thr^3^-H_2_O+H^+^ | 463.1820 |
| Tyr^1^+GA+Gln^2^+H^+^ | 380.1450 |
| Tyr^1^ Immonium | 136.0757 |
| Gln^2^ Immonium | 101.0714 |
| Leu^4^ Immonium | 86.0970 |
| Thr^3-6^ Immonium | *74.0605** |
| Fragmentation sequence | Aeruginosin A |
| M+H^+^ | 617.3458 |
| M+H^+^-CH_2_N_2_ | 575.3239 |
| [M-d-Pla^1^-CO+2H]^+^ | 469.2930 |
| [Choi+Ade^3^-CO+2H]^+^ | 322.2237 |
| [Choi+Ade^3^-CO-NH_3_+2H]^+^ | 305.1972 |
| [Choi+Ade^3^-CO+H-NH_2_-NH-C+2H]^+^ | 280.2018 |
| d-Pla^1^+Phe^2^+H^+^ | 268.1342 |
| Choi Residue | 140.1071 |
| Phe^2^ Residue | 120.0810 |
| Dehydrated Choi Immonium | 122.0965 |

*Fragments in *italic* were not selected in the FISh scoring experiment.


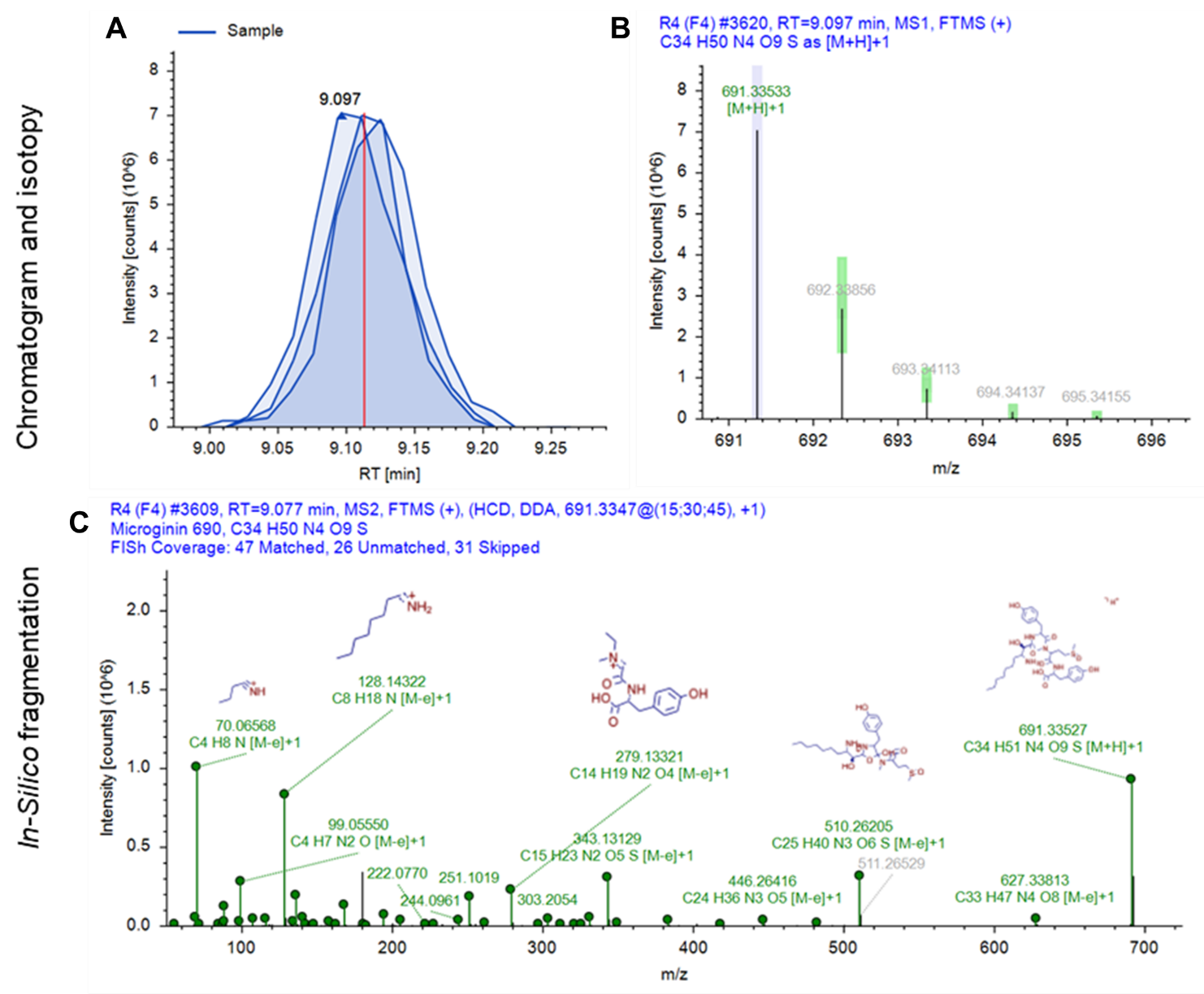


**Figure S21.** Structure characterization of Microginin 690. A) Extracted ion chromatogram of ion *m/z* 691.3350 and B) isotopic pattern of most intense precursor ion. E) Fragmentation spectrum and *in-silico* matching with FISh coverage.

The Adha moiety at position 1 is confirmed by ions *m/z* 349.2127 and the residue *m/z* 128.1436. Tyr at position 4 is confirmed by the ion at *m/z* 510.2621, and the sequence Tyr2-MeMet(O)3-Tyr4 is supported by the following ions: *m/z* 349.2127, 343.1313, 325.1210, and 297.1268. The presence of Tyr in the structure is further confirmed by its immonium ion at *m/z* 136.0755.


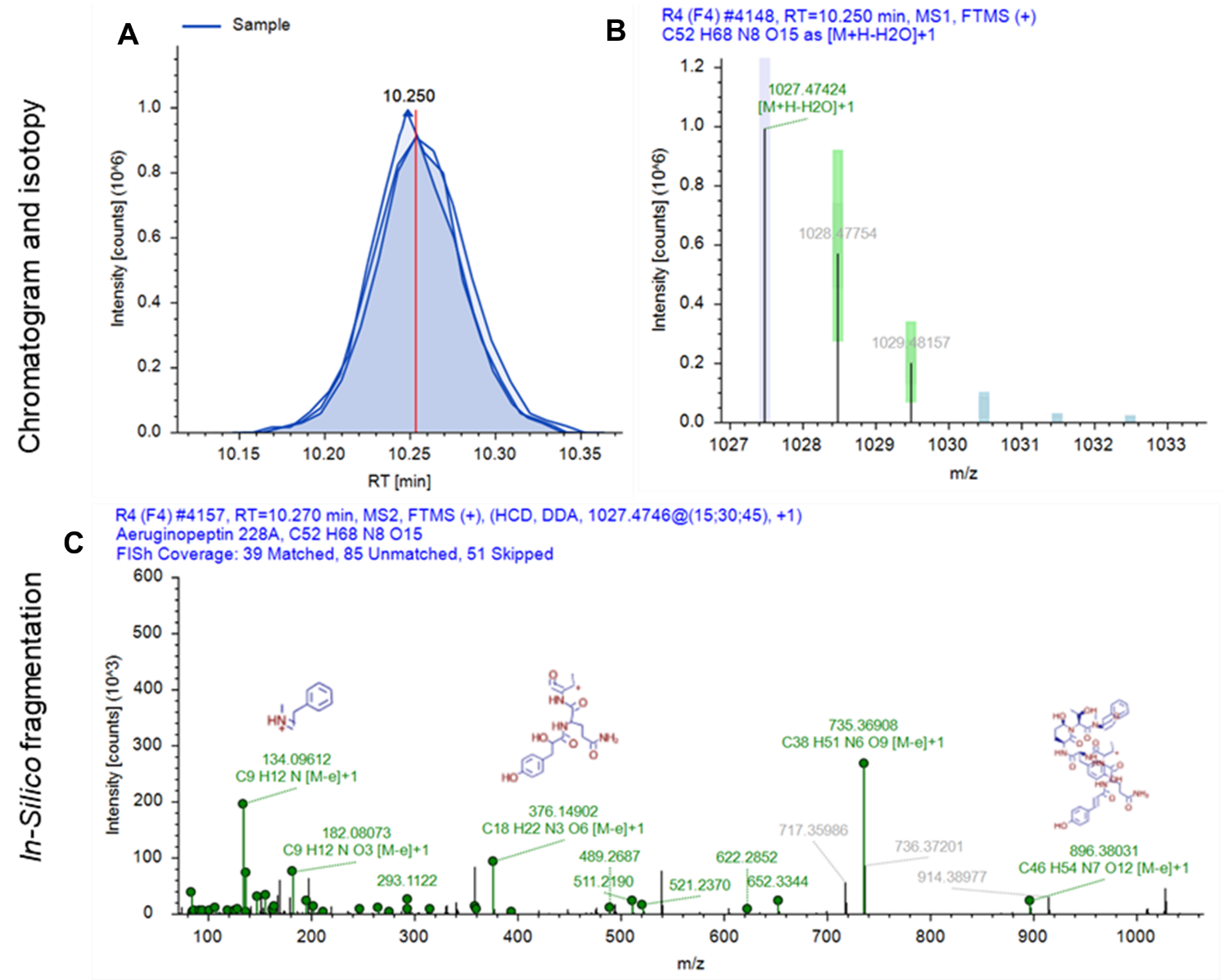


**Figure S22.** Structure characterization of Aeruginopeptin 228A. A) Extracted ion chromatogram of ion *m/z* 1045.4854 and B) isotopic pattern of most intense precursor ion. E) Fragmentation spectrum and *in-silico* matching with FISh coverage.

The ion at *m/z* 914.3898 confirms Ile at position 8, while ions *m/z* 511.2190, 265.1175, 247.1077, and 219.1125 confirm the structure Hpla1-Gln2-Thr3-Tyr4 (Table 6). Immonium ions for Gln, Thr, Tyr, Ahp, MePhe, and Ile have also been identified by the following ions: *m/z* 101.0713, 74.0606, 136.0753, 134.0961, and 86.0967. In the *in-silico* fragmentation depicted in E), we observe the generation of Ahp5-Thr6-MePhe7 fragments, including the prominent ion *m/z* 489.2689, which also include Ile at position 8.


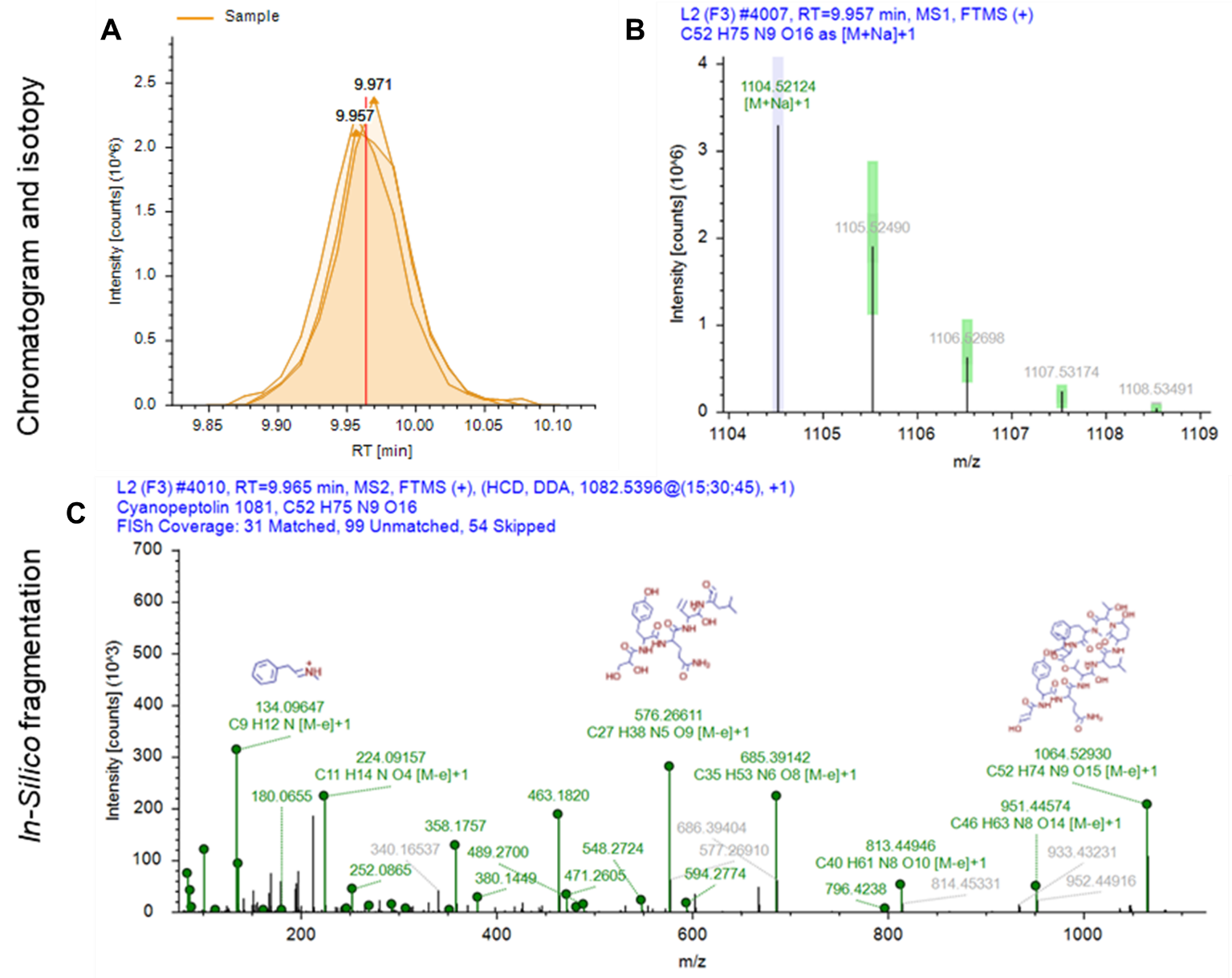


**Figure S23.** Structure characterization of Cyanopeptolin 1081. A) Extracted ion chromatogram of ion *m/z* 1082.5393 and B) isotopic pattern of most intense precursor ion. E) Fragmentation spectrum and *in-silico* matching with FISh coverage.

The spectra show the ion at *m/z* 685.3914 corresponds to positions 3 to 8 and ion at *m/z* 602.3544 corresponds to positions 4 to 8. Additionally, the ion at *m/z* 576.2661 corresponds to positions 1 to 4, *m/z* 471.2606 to positions 5 to 8, *m/z* 463.1820 to positions 1 to 3, and *m/z* 380.1450 to positions 1 to 2. Furthermore, immonium ions of Tyr, Gln, Leu, and Thr are present at *m/z* 136.0757, 101.0714, 86.0970, and 74.0605, respectively.


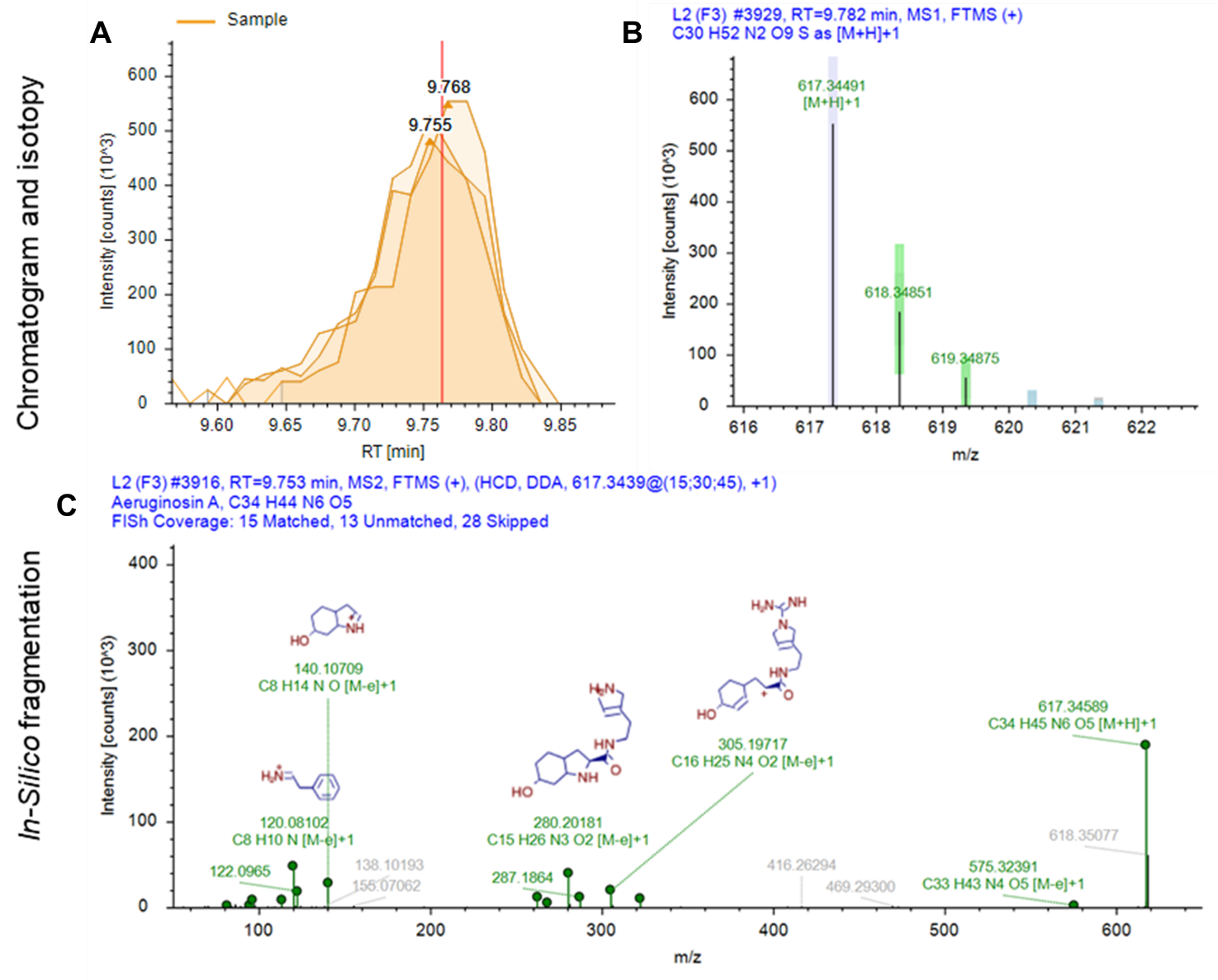


**Figure S24.** Structure characterization of Aeruginosin A. A) Extracted ion chromatogram of ion *m/z* 617.3458 and B) isotopic pattern of most intense precursor ion. E) Fragmentation spectrum and *in-silico* matching with FISh coverage.

The Choi moiety was characterized by *m/z* 140.1071, representing its residue, and *m/z* 122.0965 being its dehydrated immonium ion. These ions serve as indicators for the characterization of AGs. The proposed congener structure is d-Pla1-Phe2-Choi-Ade3, where ion *m/z* 575.3240 was identified and results from the loss of 42 u from the protonated molecule attributed to the removal of a carbodiimide (CH_2_N_2_) following the rearrangement and fragmentation of Ade (1). This difference in mass was also observed for fragments *m/z* 322.2237 and 280.2018, confirming the presence of Choi-Ade3, and the ion at 268.1342 confirms the presence of d-Pla at position 1 (1). Finally, ion *m/z* 120.0810 indicates the presence of Phe immonium ion.


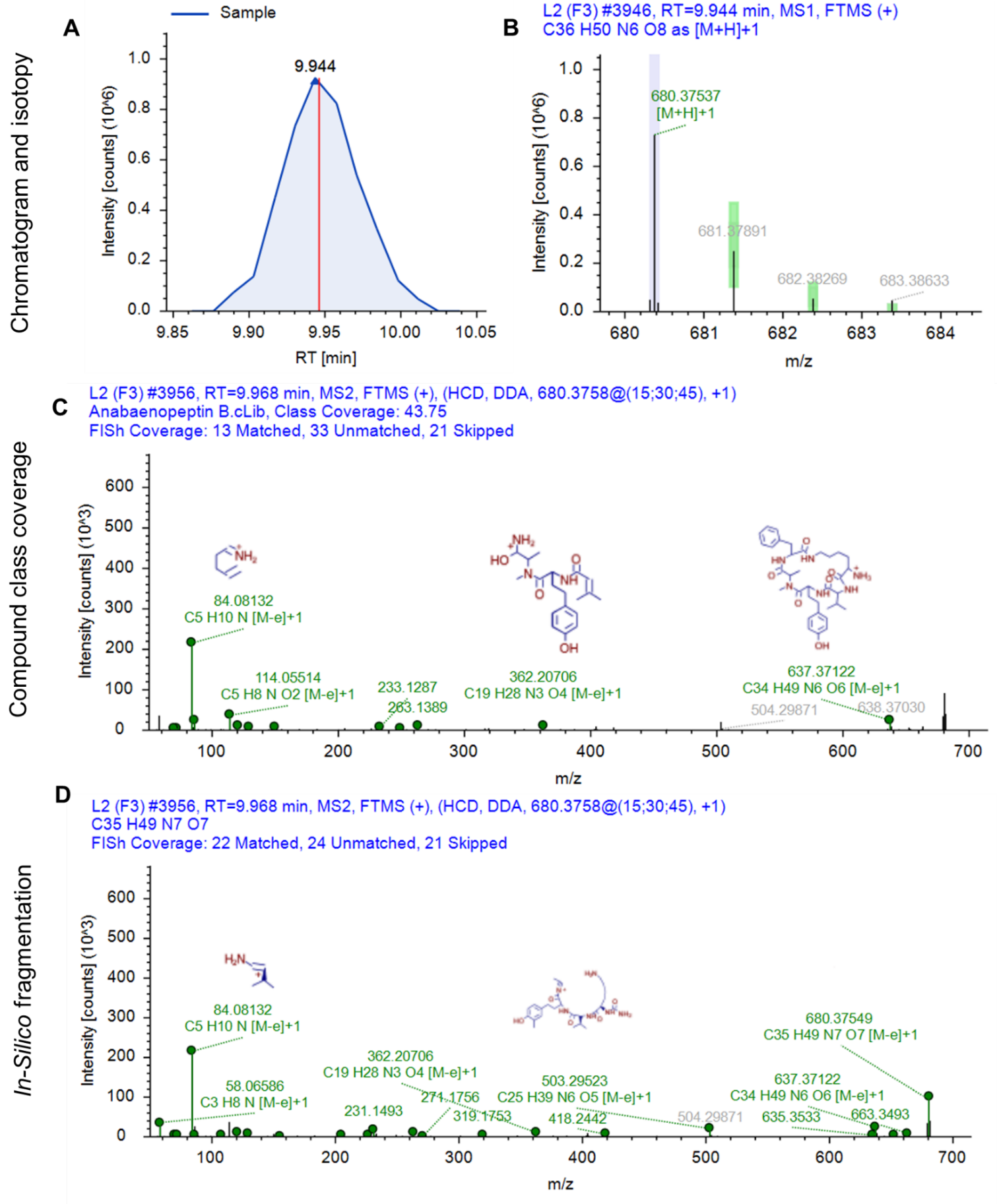


**Figure S25.** Structure characterization of transformation product 2 (TP2). A) Extracted ion chromatogram of ion *m/z* 680.3755, B) isotopic pattern of most intense precursor ion, C) Fragmentation spectrum and compound class coverage compared to Anabaenopeptin B and D) Fragmentation spectrum and *in-silico* matching with FISh coverage.


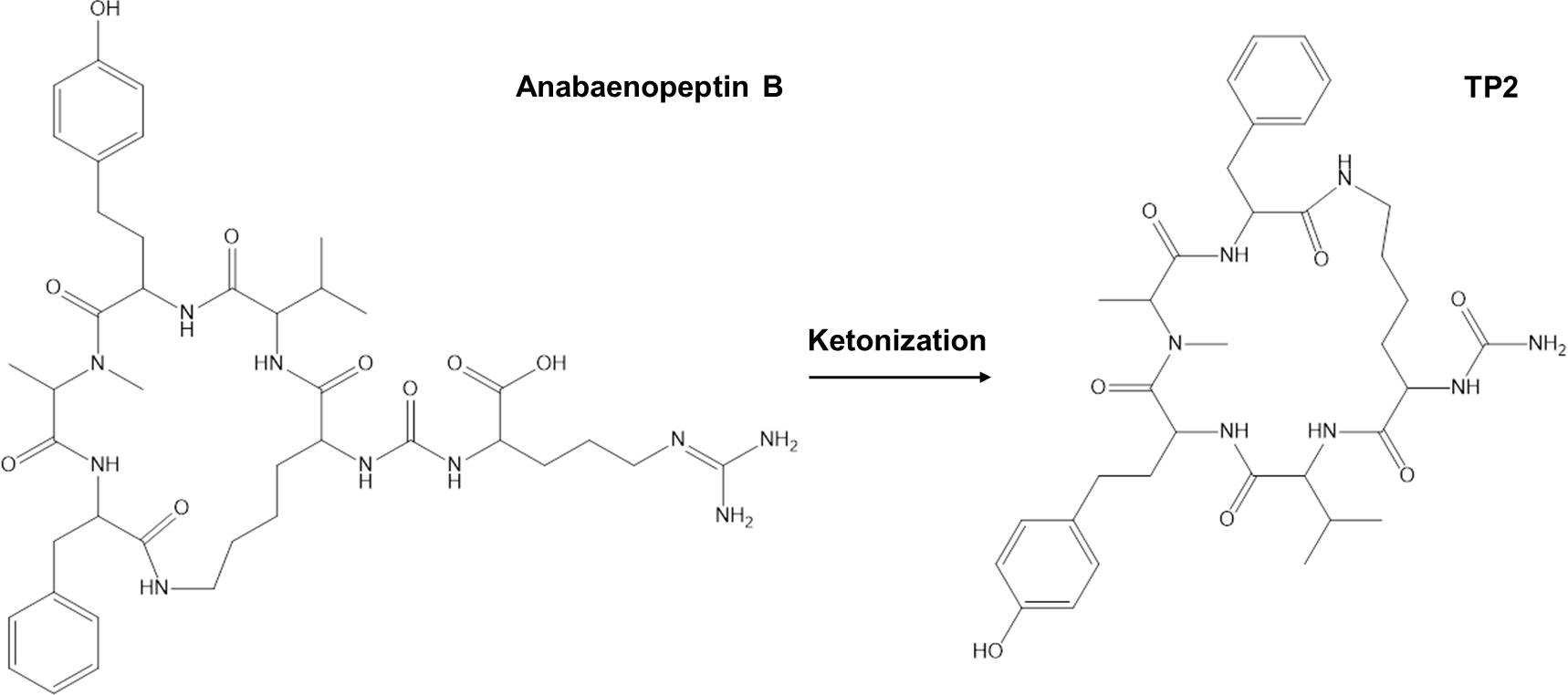


**Figure S26.** Proposed transformation product 2 (TP2) formation by ketonization of Anabaenopeptin B.


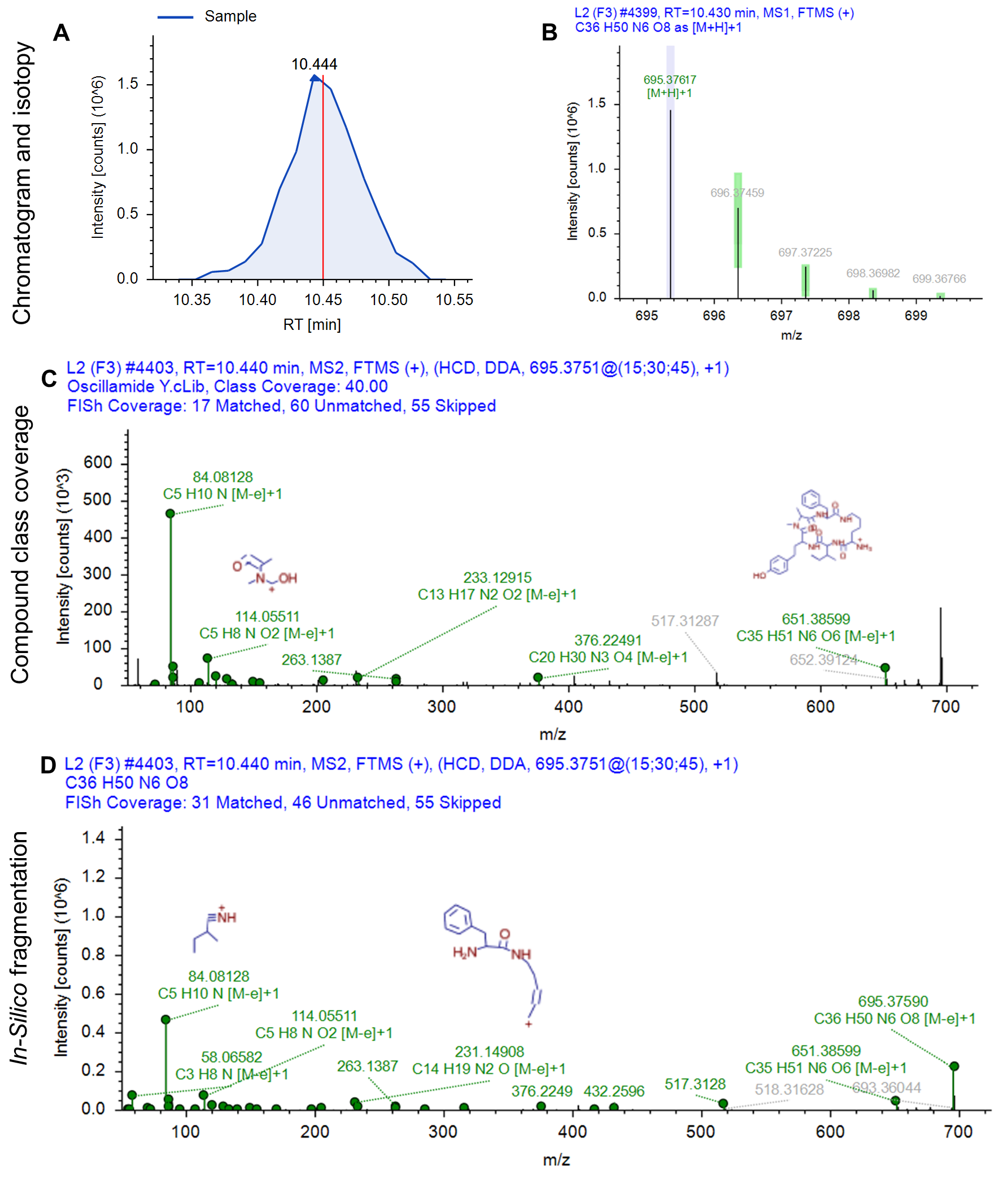


**Figure S27.** Structure characterization of transformation product 3 (TP3). A) Extracted ion chromatogram of ion *m/z* 695.3759, B) isotopic pattern of most intense precursor ion, C) Fragmentation spectrum and compound class coverage compared to Oscillamide Y and D) Fragmentation spectrum and *in-silico* matching with FISh coverage.


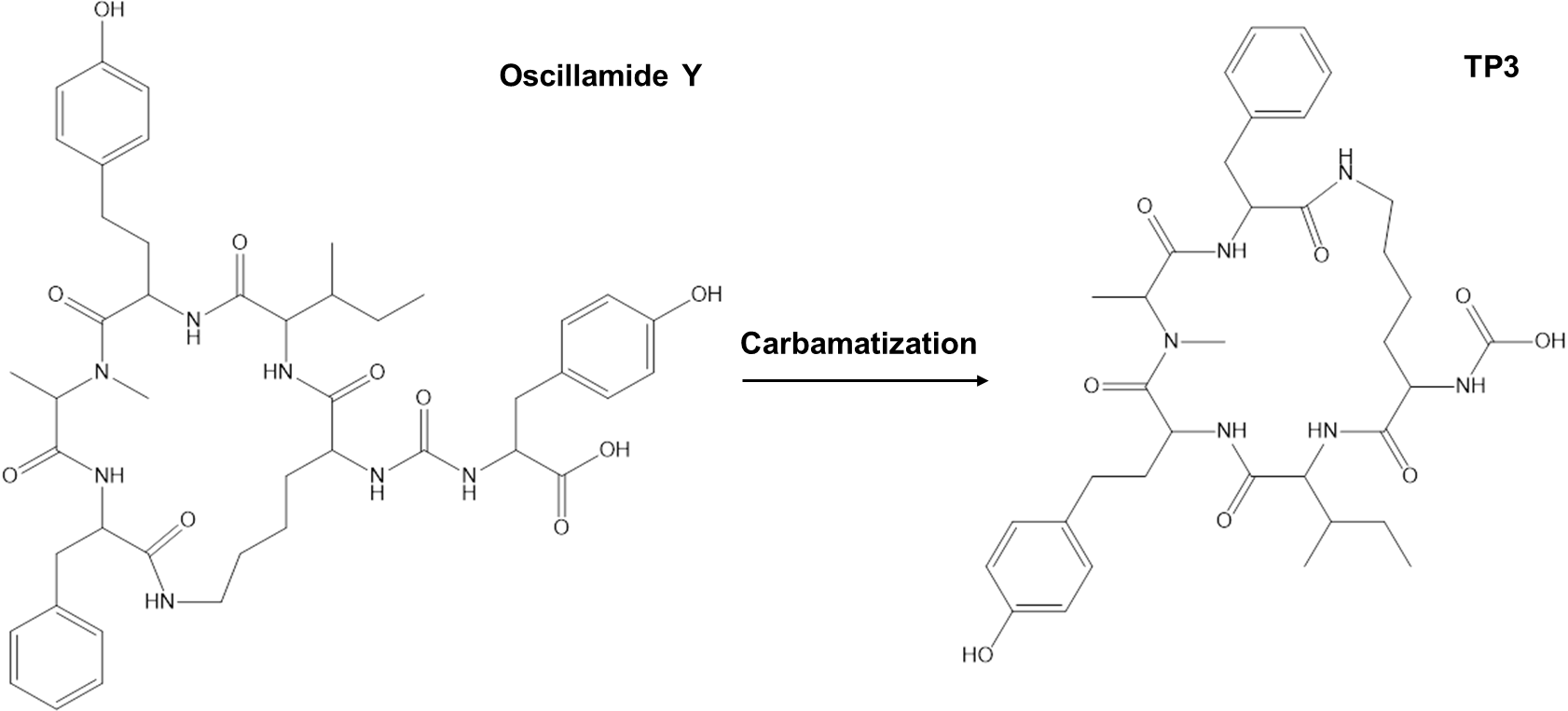


**Figure S28.** Proposed transformation product 3 (TP3) formation by carbamatization of Oscillamide Y.


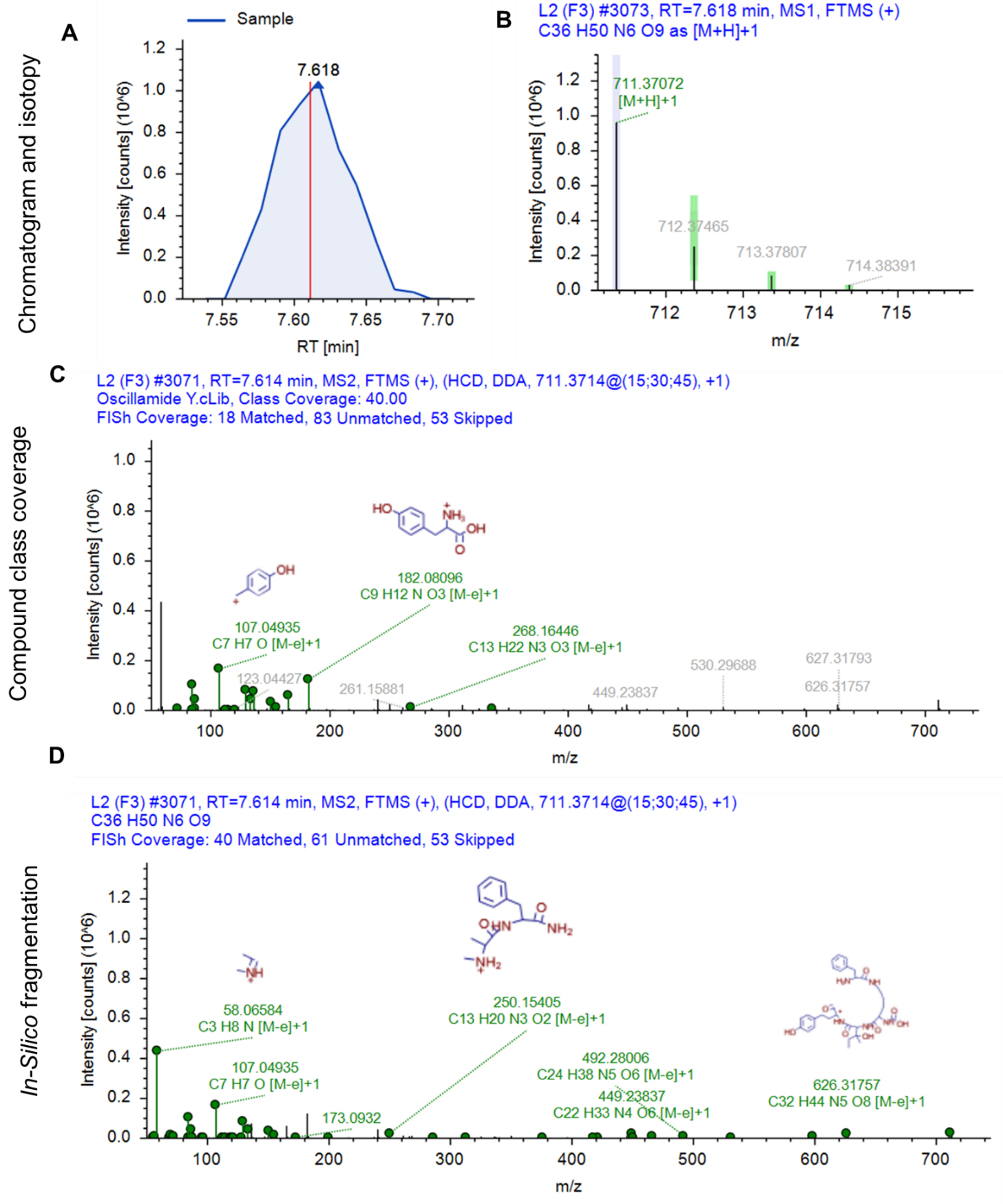


**Figure S29.** Structure characterization of transformation product 4 (TP4). A) Extracted ion chromatogram of ion *m/z* 711.3710, B) isotopic pattern of most intense precursor ion, C) Fragmentation spectrum and compound class coverage compared to Oscillamide Y and D) Fragmentation spectrum and *in-silico* matching with FISh coverage.

Two significant ions observed in the TP3 spectra (Figure S26) at *m/z* 376 (AA3+AA4+AA5+H^+^) and 417 (AA6+AA2+CO+AA3+H^+^) are absent in the TP4 spectra but are replaced by ions at m/z 392 and 433, indicating the presence of hydroxyisoleucine at position AA3.


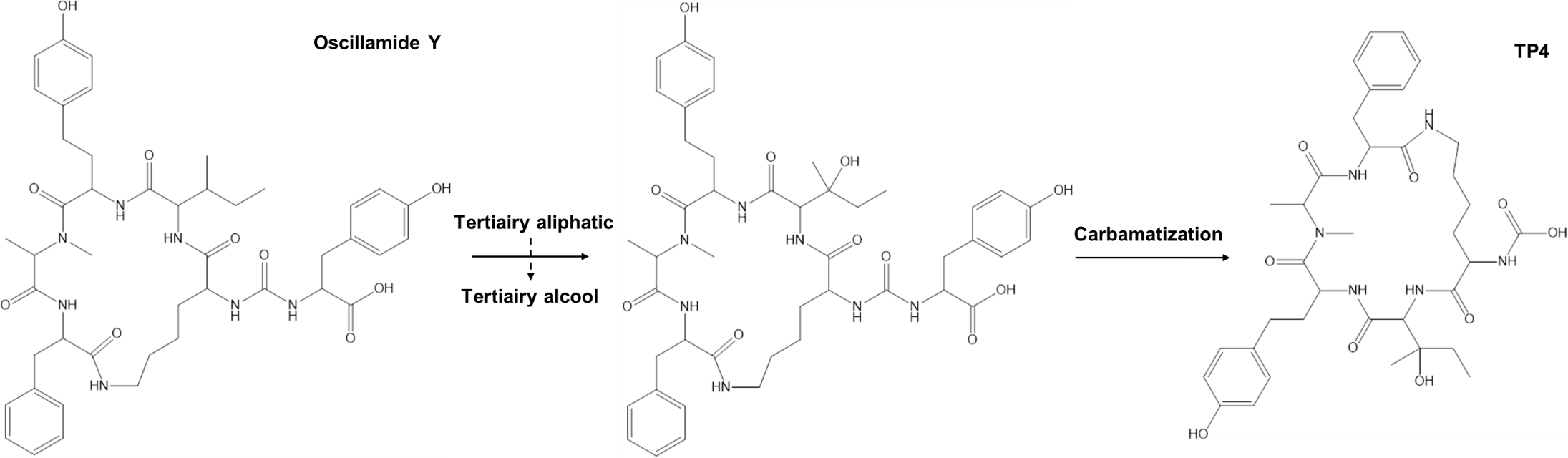


**Figure S30.** Proposed transformation product 4 (TP4) formation in two steps of Oscillamide Y.


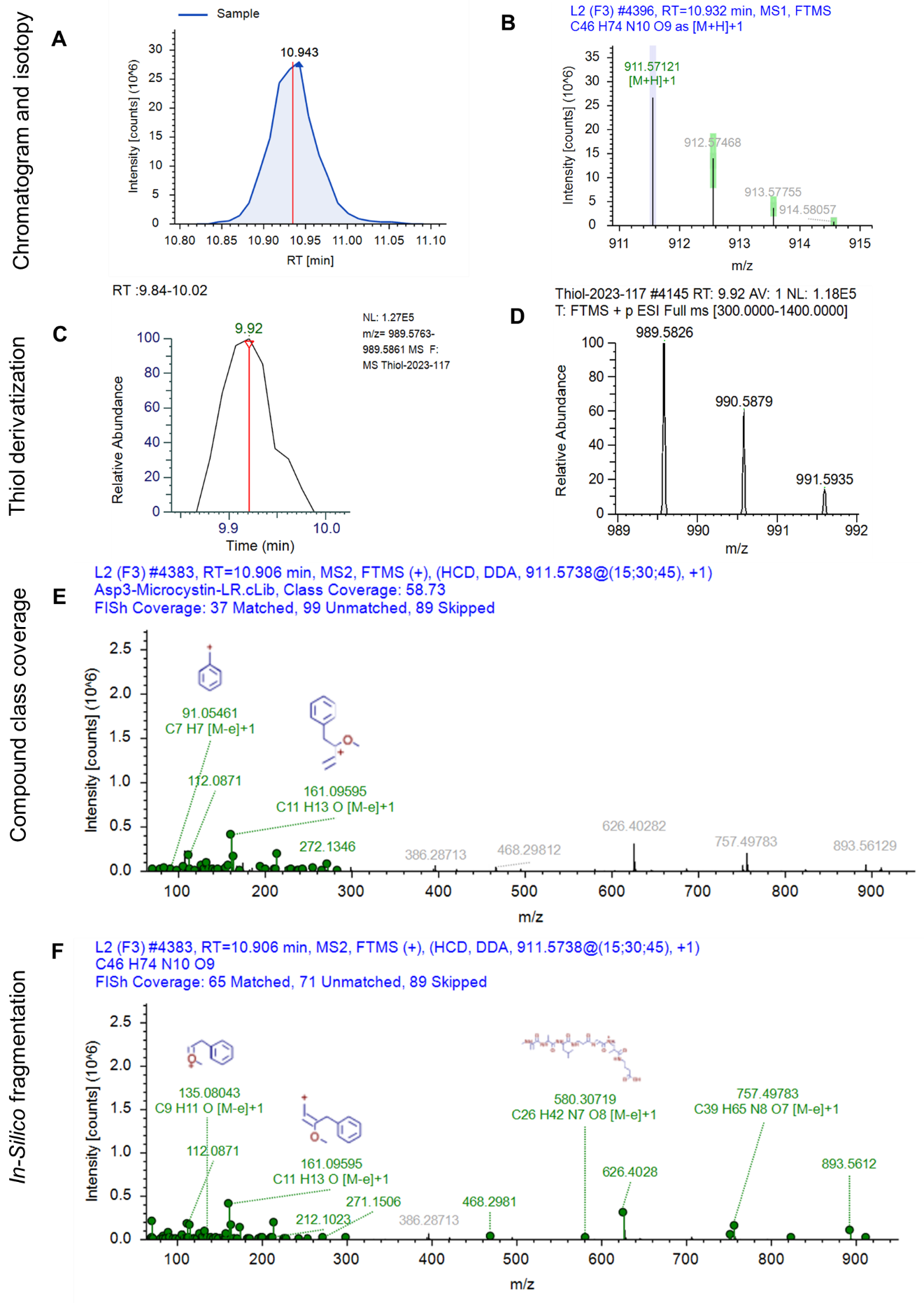


**Figure S31.** Structure characterization of transformation product 5 (TP5). A) Extracted ion chromatogram of ion *m/z* 911.5712, B) isotopic pattern of most intense precursor ion, C) Extracted ion chromatogram of thiol derivative ion *m/z* 989.5826 and D) isotopic pattern of thiol derivative. E) Fragmentation spectrum and compound class coverage compared to [Asp^3^]microcystin-LR and F) Fragmentation spectrum and *in-silico* matching with FISh coverage.

The presence of fragment *m/z* 626.4028, which includes AA3 to AA6, indicates a loss of 88, corresponding to two carboxyl groups, compared to the *m/z* 714 peak in the [Asp^3^]microcystin-LR spectra. Moreover, a thiol derivative was found at *m/z* 989.5826, confirming the presence of the alkene function in the Mdha group at AA7.


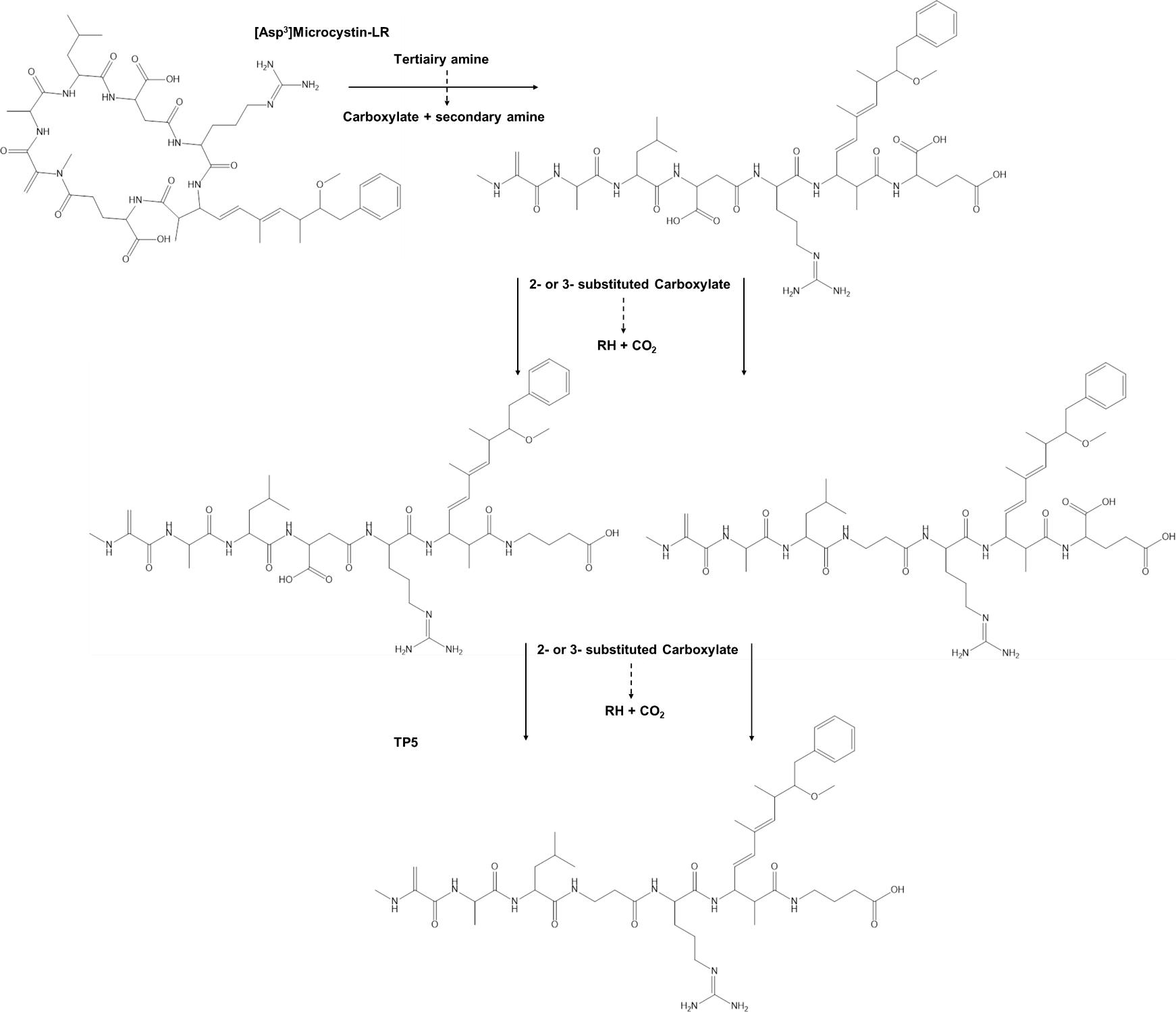


**Figure S32.** Proposed transformation product 5 (TP5) formation in two steps of [Asp^3^]microcystin-LR.


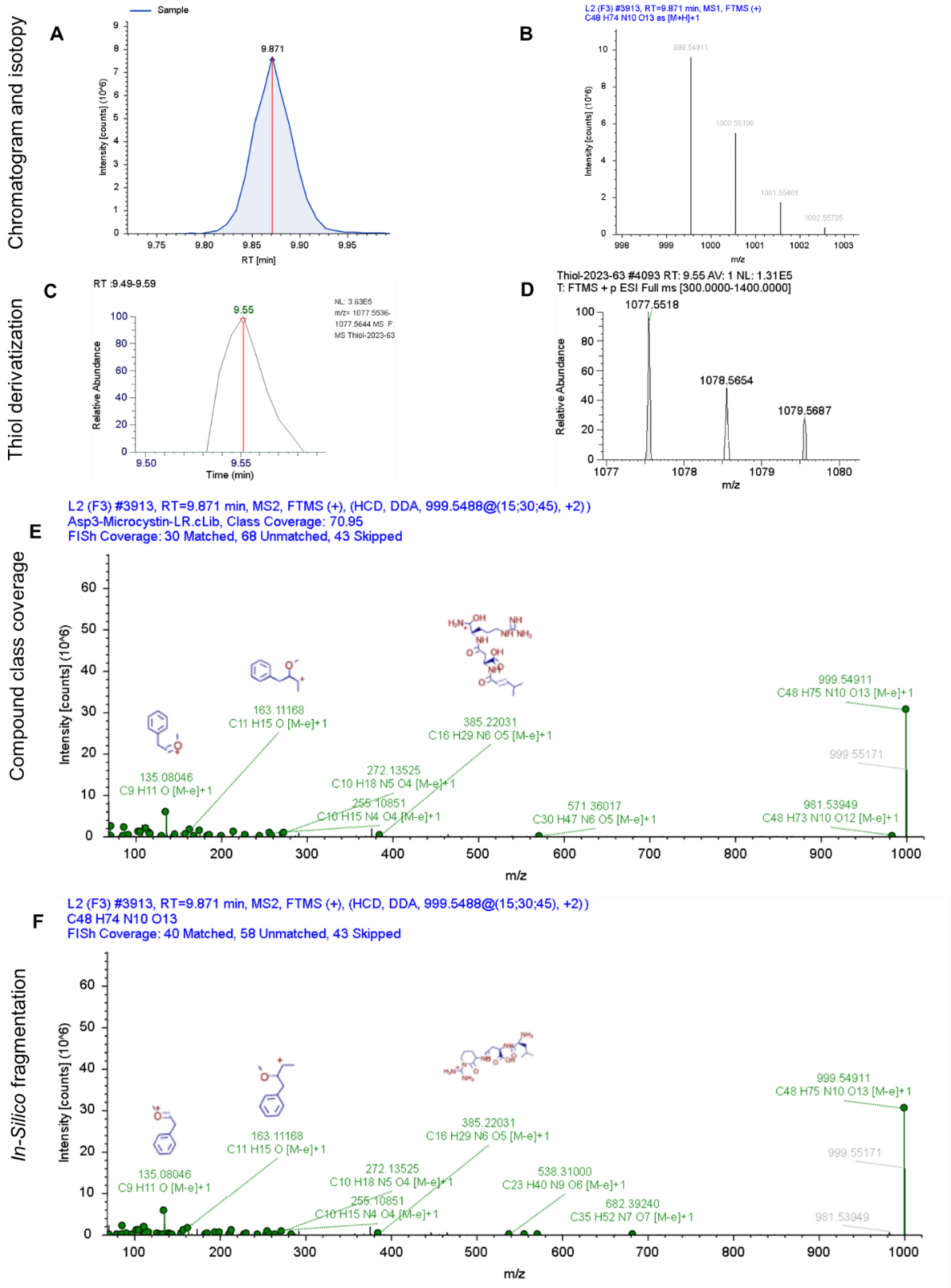


**Figure S33.** Structure characterization of transformation product 6 (TP6). A) Extracted ion chromatogram of ion *m/z* 999.5491, B) isotopic pattern of most intense precursor ion, C) Extracted ion chromatogram of thiol derivative ion *m/z* 1077.5518 and D) isotopic pattern of thiol derivative. E) Fragmentation spectrum and compound class coverage compared to [Asp^3^]microcystin-LR and F) Fragmentation spectrum and *in-silico* matching with FISh coverage.

The seco-1/7 form can be verified by the presence of characteristic peaks at *m/z* 714, 599, 571, 385, 368, and 174, among others, which are associated with the chain Tyr2-Asp3-Arg4-Adda5-Glu6-Mdha7. Besides, the characteristic peaks at 127 and 155, associated with the AA1-AA7 linkage, are absent from the spectra, indicating the ring opening at these positions. Furthermore, a thiol derivative was found at *m/z* 1077.5518, confirming the presence of Mdha at AA7 position.


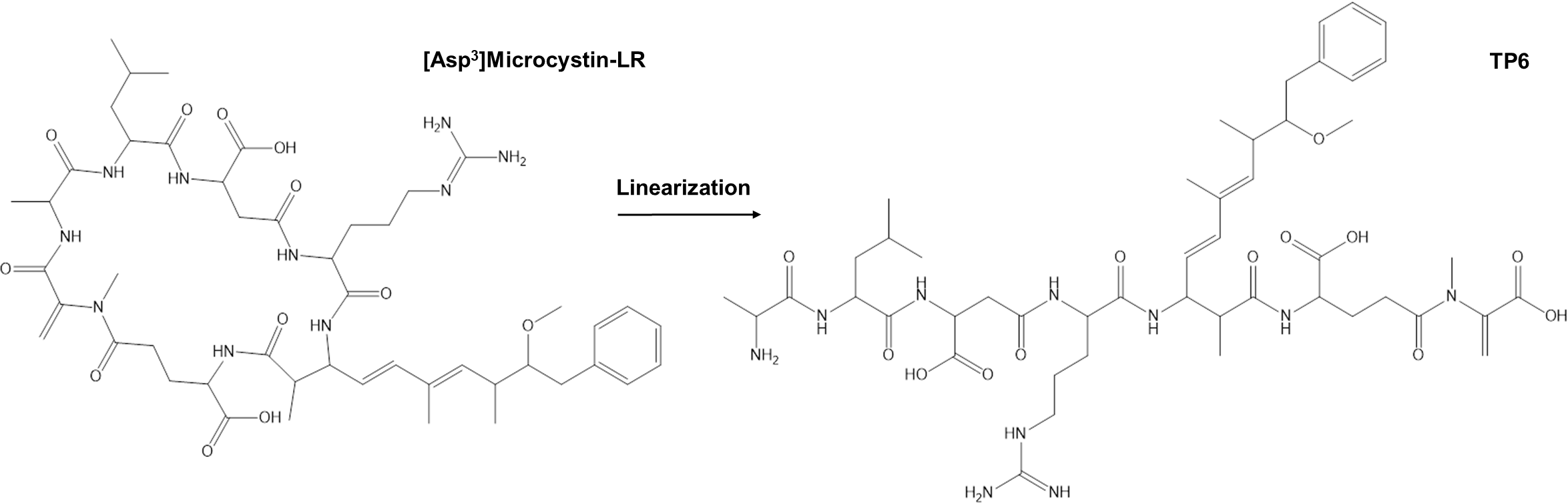


**Figure S34.** Proposed transformation product 6 (TP6) formation by linearization of [Asp^3^]microcystin-LR.


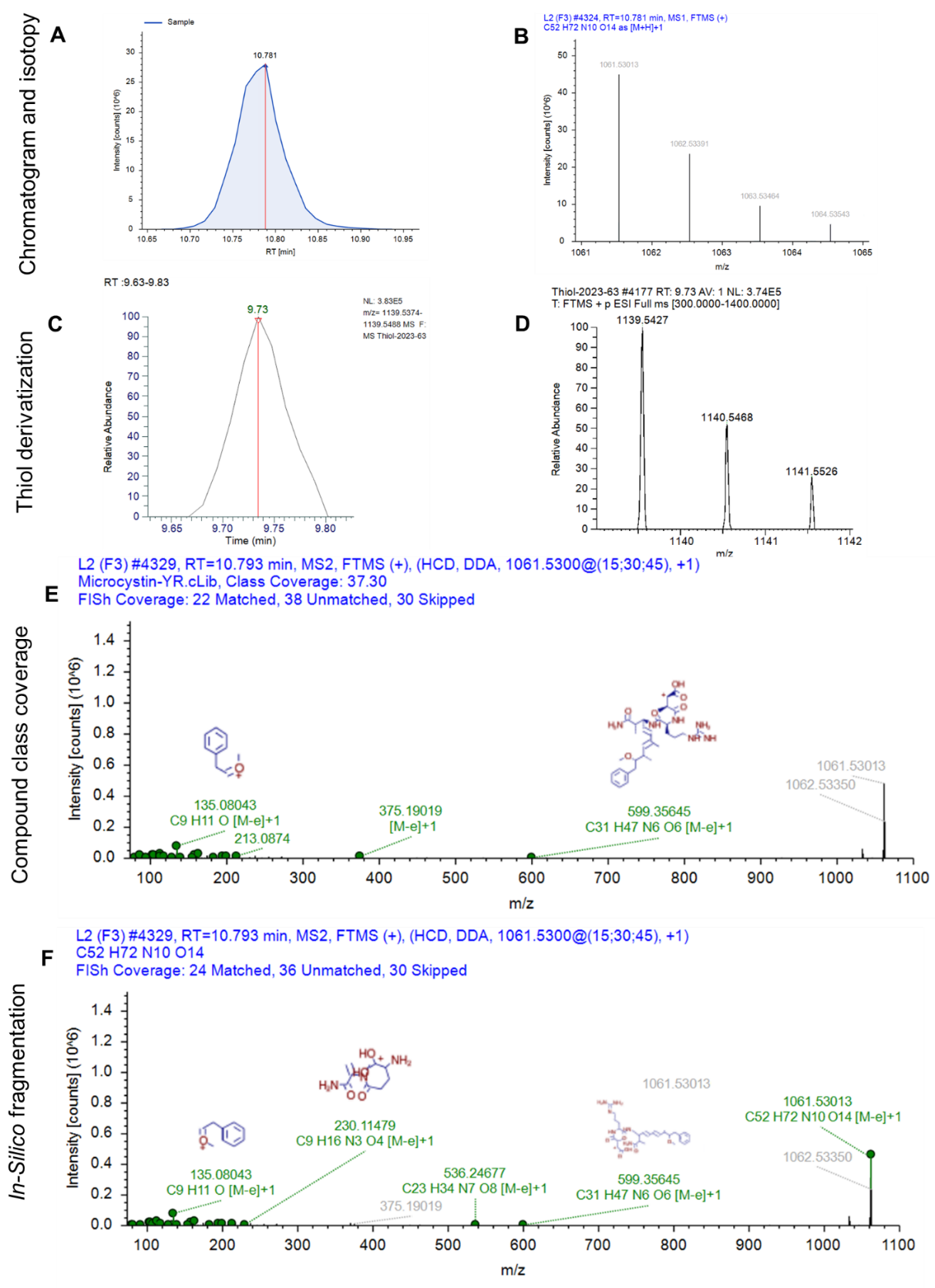


**Figure S35.** Structure characterization of transformation product 7 (TP7). A) Extracted ion chromatogram of ion *m/z* 1061.5301, B) isotopic pattern of most intense precursor ion, C) Extracted ion chromatogram of thiol derivative ion *m/z* 1139.5427 and D) isotopic pattern of thiol derivative. E) Fragmentation spectrum and compound class coverage compared to microcystin-YR and F) Fragmentation spectrum and *in-silico* matching with FISh coverage.

The main peaks associated with microcystin-YR are present in the spectra, including *m/z* 599, 375, 213, 155, 135, and 127. These peaks correspond to amino acids AA1, 3, 4, 6, and 7. Additionally, a peak at 536 indicates the hydroxylation of Tyr (AA2) to dopamine. A thiol derivative was found at *m/z* 1139.5427, confirming the presence of Mdha at AA7 position.


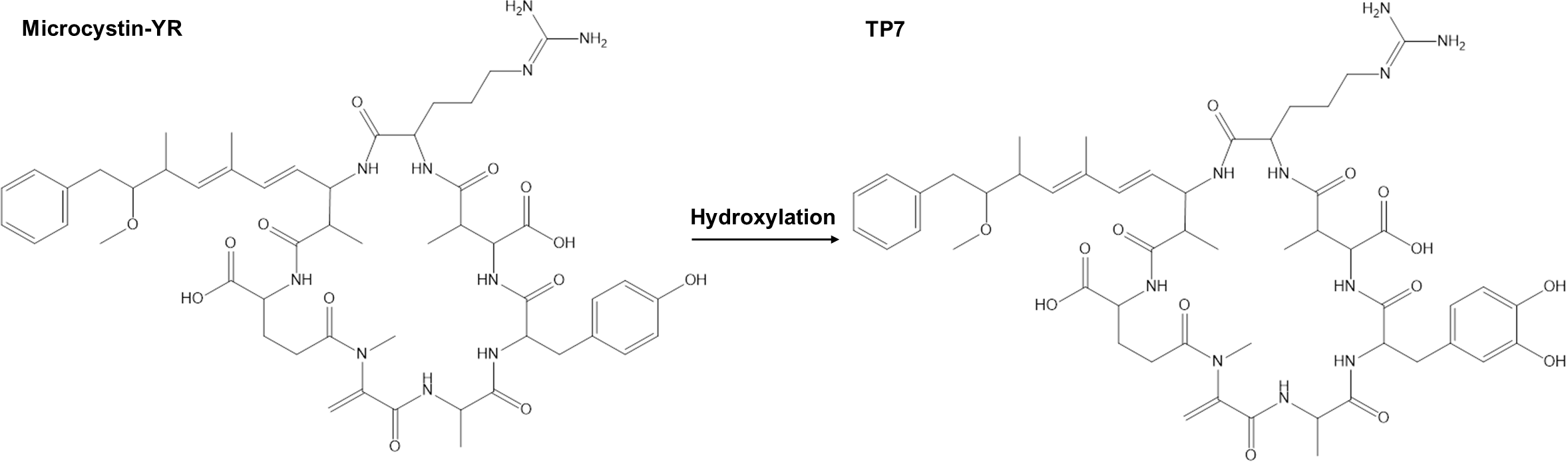


**Figure S36.** Proposed transformation product 7 (TP7) formation by linearization of microcystin-YR.

**References**

1. Otto JFM, Kiel C, Nejstgaard JC, Pohnert G, Berger SA, Ueberschaar N. Tracking a broad inventory of cyanotoxins and related secondary metabolites using UHPLC-HRMS. J Hazard Mater Adv. 2023;12:100370.
