# Supplementary material for: Characterizing cyanopeptides and transformation products in freshwater: integrating targeted, suspect, and non-targeted analysis with in silico modeling
Source: Anal Bioanal Chem. 2025 Jul 12;417(21):4829–46. doi: 10.1007/s00216-025-05999-6 (PMC12367966; doi:10.1007/s00216-025-05999-6)
Supplement: Supplementary file 2 — Supplementary file2 (DOCX 95 KB) [file 216_2025_5999_MOESM2_ESM.docx]

**SUPPLEMENTARY INFORMATION 2**

| **Compound Classes** | | |
| --- | --- | --- |
| ***m/z*** | **Molecular Formula** | **Charge State** |
| **Aeruginoguanidine 98A** | | |
| 70.06513 | C4H8N | 1 |
| 84.08078 | C5H10N | 1 |
| 112.08692 | C5H10N3 | 1 |
| 129.10224 | C6H13N2O | 1 |
| 265.16590 | C13H21N4O2 | 1 |
| 982.40424 | C39H68N9O14S3 | 1 |
| **Aeruginosin 98A** | | |
| 60.05562 | CH6N3 | 1 |
| 72.08078 | C4H10N | 1 |
| 86.09643 | C5H12N | 1 |
| 79.05423 | C6H7 | 1 |
| 97.07602 | C5H9N2 | 1 |
| 99.09167 | C5H11N2 | 1 |
| 114.10257 | C5H12N3 | 1 |
| 131.12912 | C5H15N4 | 1 |
| 141.11347 | C6H13N4 | 1 |
| 263.18664 | C14H23N4O | 1 |
| 280.21319 | C14H26N5O | 1 |
| 284.10480 | C14H19ClNO3 | 1 |
| 378.18057 | C14H28N5O5S | 1 |
| 591.30562 | C29H44ClN6O5 | 1 |
| 221.16484 | C13H21N2O | 1 |
| 479.19186 | C17H31N6O8S | 1 |
| 689.27300 | C29H46ClN6O9S | 1 |
| **Aeruginosin 98B** | | |
| 60.05562 | CH6N3 | 1 |
| 72.08078 | C4H10N | 1 |
| 86.09643 | C5H12N | 1 |
| 99.09167 | C5H11N2 | 1 |
| 114.10257 | C5H12N3 | 1 |
| 131.12912 | C5H15N4 | 1 |
| 141.11347 | C6H13N4 | 1 |
| 220.06381 | C8H14NO4S | 1 |
| 221.16484 | C13H21N2O | 1 |
| 250.14377 | C14H20NO3 | 1 |
| 263.18664 | C14H23N4O | 1 |
| 280.21319 | C14H26N5O | 1 |
| 378.18057 | C14H28N5O5S | 1 |
| 557.34459 | C29H45N6O5 | 1 |
| 655.31197 | C29H47N6O9S | 1 |

**Table 1.** FISh-generated fragments using Compound Discoverer 3.3 from standards for compound classes.

| **Anabaenopeptin 915** | | |
| --- | --- | --- |
| 72.08078 | C4H10N | 1 |
| 84.08078 | C5H10N | 1 |
| 86.06004 | C4H8NO | 1 |
| 107.04914 | C7H7O | 1 |
| 114.05496 | C5H8NO2 | 1 |
| 120.08078 | C8H10N | 1 |
| 129.10224 | C6H13N2O | 1 |
| 133.06479 | C9H9O | 1 |
| 136.07569 | C8H10NO | 1 |
| 150.09134 | C9H12NO | 1 |
| 155.08150 | C7H11N2O2 | 1 |
| 164.10699 | C10H14NO | 1 |
| 165.05462 | C9H9O3 | 1 |
| 182.08117 | C9H12NO3 | 1 |
| 192.10191 | C11H14NO2 | 1 |
| 209.12845 | C11H17N2O2 | 1 |
| 220.09682 | C12H14NO3 | 1 |
| 235.14410 | C13H19N2O2 | 1 |
| 249.15975 | C14H21N2O2 | 1 |
| 254.14992 | C12H20N3O3 | 1 |
| 275.17540 | C16H23N2O2 | 1 |
| 277.15467 | C15H21N2O3 | 1 |
| 277.19105 | C16H25N2O2 | 1 |
| 305.18597 | C17H25N2O3 | 1 |
| 308.16048 | C15H22N3O4 | 1 |
| 336.15540 | C16H22N3O5 | 1 |
| 341.18597 | C20H25N2O3 | 1 |
| 369.18088 | C21H25N2O4 | 1 |
| 403.23398 | C21H31N4O4 | 1 |
| 419.22890 | C21H31N4O5 | 1 |
| 421.24455 | C21H33N4O5 | 1 |
| 431.22890 | C22H31N4O5 | 1 |
| 435.22381 | C21H31N4O6 | 1 |
| 445.24455 | C23H33N4O5 | 1 |
| 449.23946 | C22H33N4O6 | 1 |
| 468.24930 | C26H34N3O5 | 1 |
| 581.33336 | C32H45N4O6 | 1 |
| 612.33918 | C32H46N5O7 | 1 |
| 622.32353 | C33H44N5O7 | 1 |
| 709.42832 | C38H57N6O7 | 1 |
| 721.39194 | C38H53N6O8 | 1 |
| 735.40759 | C39H55N6O8 | 1 |
| 739.40250 | C38H55N6O9 | 1 |
| 858.43962 | C45H60N7O10 | 1 |
| 888.48657 | C47H66N7O10 | 1 |
| 898.47092 | C48H64N7O10 | 1 |
| 916.48148 | C48H66N7O11 | 1 |
| **Anabaenopeptin A** | | |
| 72.08078 | C4H10N | 1 |
| 84.08078 | C5H10N | 1 |
| 86.06004 | C4H8NO | 1 |
| 107.04914 | C7H7O | 1 |
| 114.05496 | C5H8NO2 | 1 |
| 120.08078 | C8H10N | 1 |
| 129.10224 | C6H13N2O | 1 |
| 136.07569 | C8H10NO | 1 |
| 150.09134 | C9H12NO | 1 |
| 155.08150 | C7H11N2O2 | 1 |
| 165.05462 | C9H9O3 | 1 |
| 182.08117 | C9H12NO3 | 1 |
| 205.13354 | C12H17N2O | 1 |
| 209.12845 | C11H17N2O2 | 1 |
| 233.12845 | C13H17N2O2 | 1 |
| 235.14410 | C13H19N2O2 | 1 |
| 249.15975 | C14H21N2O2 | 1 |
| 254.14992 | C12H20N3O3 | 1 |
| 263.13902 | C14H19N2O3 | 1 |
| 277.15467 | C15H21N2O3 | 1 |
| 320.16048 | C16H22N3O4 | 1 |
| 362.20743 | C19H28N3O4 | 1 |
| 336.15540 | C16H22N3O5 | 1 |
| 367.20162 | C22H27N2O3 | 1 |
| 379.23398 | C19H31N4O4 | 1 |
| 401.21833 | C21H29N4O4 | 1 |
| 412.22308 | C23H30N3O4 | 1 |
| 419.2289 | C21H31N4O5 | 1 |
| 435.22381 | C21H31N4O6 | 1 |
| 437.21833 | C24H29N4O4 | 1 |
| 483.22381 | C25H31N4O6 | 1 |
| 455.22890 | C24H31N4O5 | 1 |
| 509.27585 | C28H37N4O5 | 1 |
| 536.28675 | C29H38N5O5 | 1 |
| 540.28166 | C28H38N5O6 | 1 |
| 568.27657 | C29H38N5O7 | 1 |
| 635.35516 | C34H47N6O6 | 1 |
| 637.37081 | C34H49N6O6 | 1 |
| 639.35007 | C33H47N6O7 | 1 |
| 649.33442 | C34H45N6O7 | 1 |
| 663.35007 | C35H47N6O7 | 1 |
| 667.34499 | C34H47N6O8 | 1 |
| 681.36064 | C35H49N6O8 | 1 |
| 816.42905 | C43H58N7O9 | 1 |
| 826.41340 | C44H56N7O9 | 1 |
| 827.42123 | C44H57N7O9 | 1 |
| 844.42397 | C44H58N7O10 | 1 |
| **Anabaenopeptin B** | | |
| 60.05562 | CH6N3 | 1 |
| 70.06513 | C4H8N | 1 |
| 71.04780 | C2H5N3 | 1 |
| 72.08078 | C4H10N | 1 |
| 84.08078 | C5H10N | 1 |
| 86.03489 | C2H4N3O | 1 |
| 86.06004 | C4H8NO | 1 |
| 112.08692 | C5H10N3 | 1 |
| 113.07094 | C5H9N2O | 1 |
| 114.05496 | C5H8NO2 | 1 |
| 115.08659 | C5H11N2O | 1 |
| 116.07061 | C5H10NO2 | 1 |
| 120.08078 | C8H10N | 1 |
| 129.10224 | C6H13N2O | 1 |
| 130.09749 | C5H12N3O | 1 |
| 141.06585 | C6H9N2O2 | 1 |
| 142.04987 | C6H8NO3 | 1 |
| 150.09134 | C9H12NO | 1 |
| 157.10839 | C6H13N4O | 1 |
| 158.09240 | C6H12N3O2 | 1 |
| 159.07642 | C6H11N2O3 | 1 |
| 173.10330 | C6H13N4O2 | 1 |
| 175.11895 | C6H15N4O2 | 1 |
| 183.08765 | C7H11N4O2 | 1 |
| 201.09822 | C7H13N4O3 | 1 |
| 233.12845 | C13H17N2O2 | 1 |
| 249.15975 | C14H21N2O2 | 1 |
| 263.13902 | C14H19N2O3 | 1 |
| 277.15467 | C15H21N2O3 | 1 |
| 362.20743 | C19H28N3O4 | 1 |
| 637.37081 | C34H49N6O6 | 1 |
| 837.46175 | C41H61N10O9 | 1 |
| **Anabaenopeptin E** | | |
| 60.05562 | CH6N3 | 1 |
| 70.06513 | C4H8N | 1 |
| 81.03349 | C5H5O | 1 |
| 84.08078 | C5H10N | 1 |
| 86.09643 | C5H12N | 1 |
| 86.06004 | C4H8NO | 1 |
| 99.04406 | C5H7O2 | 1 |
| 107.04914 | C7H7O | 1 |
| 112.08692 | C5H10N3 | 1 |
| 113.07094 | C5H9N2O | 1 |
| 114.05496 | C5H8NO2 | 1 |
| 115.08659 | C5H11N2O | 1 |
| 116.07061 | C5H10NO2 | 1 |
| 120.08078 | C8H10N | 1 |
| 129.10224 | C6H13N2O | 1 |
| 130.09749 | C5H12N3O | 1 |
| 141.06585 | C6H9N2O2 | 1 |
| 142.04987 | C6H8NO3 | 1 |
| 157.10839 | C6H13N4O | 1 |
| 158.09240 | C6H12N3O2 | 1 |
| 159.07642 | C6H11N2O3 | 1 |
| 173.10330 | C6H13N4O2 | 1 |
| 175.11895 | C6H15N4O2 | 1 |
| 201.09822 | C7H13N4O3 | 1 |
| 205.10973 | C12H15NO2 | 1 |
| 233.12845 | C13H17N2O2 | 1 |
| 263.17540 | C15H23N2O2 | 1 |
| 376.22308 | C20H30N3O4 | 1 |
| 651.38646 | C35H51N6O6 | 1 |
| 851.47740 | C42H63N10O9 | 1 |
| **Anabaenopeptin J** | | |
| 72.08078 | C4H10N | 1 |
| 84.08078 | C5H10N | 1 |
| 86.09643 | C5H12N | 1 |
| 91.05423 | C7H7 | 1 |
| 107.04914 | C7H7O | 1 |
| 129.10224 | C6H13N2O | 1 |
| 150.09134 | C9H12NO | 1 |
| 155.08150 | C7H11N2O2 | 1 |
| 173.09207 | C7H13N2O3 | 1 |
| 205.13354 | C12H17N2O | 1 |
| 233.12845 | C13H17N2O2 | 1 |
| 254.14992 | C12H20N3O3 | 1 |
| 249.15975 | C14H21N2O2 | 1 |
| 277.15467 | C15H21N2O3 | 1 |
| 419.26528 | C22H35N4O4 | 1 |
| 490.30240 | C25H40N5O5 | 1 |
| 509.27585 | C28H37N4O5 | 1 |
| 637.37081 | C34H49N6O6 | 1 |
| 635.35516 | C34H47N6O6 | 1 |
| 663.35007 | C35H47N6O7 | 1 |
| 766.44979 | C40H60N7O8 | 1 |
| 776.43414 | C41H58N7O8 | 1 |
| 794.44470 | C41H60N7O9 | 1 |
| **[Asp^3^]Microcystin-LR** | | |
| 70.02874 | C3H4NO | 1 |
| 77.03858 | C6H5 | 1 |
| 79.05423 | C6H7 | 1 |
| 84.04439 | C4H6NO | 1 |
| 86.09643 | C5H12N | 1 |
| 91.05423 | C7H7 | 1 |
| 98.06004 | C5H8NO | 1 |
| 103.05423 | C8H7 | 1 |
| 105.06988 | C8H9 | 1 |
| 112.08692 | C5H10N3 | 1 |
| 113.07094 | C5H9N2O | 1 |
| 115.05423 | C9H7 | 1 |
| 117.06988 | C9H9 | 1 |
| 119.08553 | C9H11 | 1 |
| 127.08659 | C6H11N2O | 1 |
| 130.04987 | C5H8NO3 | 1 |
| 131.08553 | C10H11 | 1 |
| 133.06077 | C4H9N2O3 | 1 |
| 133.10118 | C10H13 | 1 |
| 135.08044 | C9H11O | 1 |
| 140.08184 | C6H10N3O | 1 |
| 141.06585 | C6H9N2O2 | 1 |
| 143.08553 | C11H11 | 1 |
| 145.10118 | C11H13 | 1 |
| 155.08150 | C7H11N2O2 | 1 |
| 157.10839 | C6H13N4O | 1 |
| 161.09609 | C11H13O | 1 |
| 163.11174 | C11H15O | 1 |
| 171.11683 | C13H15 | 1 |
| 183.08765 | C7H11N4O2 | 1 |
| 185.12845 | C9H17N2O2 | 1 |
| 195.07642 | C9H11N2O3 | 1 |
| 200.11420 | C7H14N5O2 | 1 |
| 209.13248 | C16H17 | 1 |
| 211.11895 | C9H15N4O2 | 1 |
| 212.10297 | C9H14N3O3 | 1 |
| 213.08698 | C9H13N2O4 | 1 |
| 227.11387 | C9H15N4O3 | 1 |
| 230.11353 | C9H16N3O4 | 1 |
| 237.09822 | C10H13N4O3 | 1 |
| 239.10263 | C11H15N2O4 | 1 |
| 244.14042 | C9H18N5O3 | 1 |
| 254.12477 | C10H16N5O3 | 1 |
| 255.10878 | C10H15N4O4 | 1 |
| 258.18524 | C17H24NO | 1 |
| 265.15869 | C19H21O | 1 |
| 266.11353 | C12H16N3O4 | 1 |
| 268.16557 | C13H22N3O3 | 1 |
| 272.13533 | C10H18N5O4 | 1 |
| 282.18524 | C19H24NO | 1 |
| 310.10336 | C13H16N3O6 | 1 |
| 383.19251 | C17H27N4O6 | 1 |
| 284.12410 | C12H18N3O5 | 1 |
| 385.21939 | C16H29N6O5 | 1 |
| 368.19285 | C16H26N5O5 | 1 |
| 439.22996 | C19H31N6O6 | 1 |
| 539.29362 | C23H39N8O7 | 1 |
| 571.36025 | C30H47N6O5 | 1 |
| 599.35516 | C31H47N6O6 | 1 |
| 711.34203 | C29H47N10O11 | 1 |
| 847.46723 | C39H63N10O11 | 1 |
| 981.54039 | C48H73N10O12 | 1 |
| **[Asp^3^]Microcystin-RR** | | |
| 77.03858 | C6H5 | 1 |
| 79.05423 | C6H7 | 1 |
| 84.04439 | C4H6NO | 1 |
| 86.09643 | C5H12N | 1 |
| 91.05423 | C7H7 | 1 |
| 98.06004 | C5H8NO | 1 |
| 102.05496 | C4H8NO2 | 1 |
| 103.05423 | C8H7 | 1 |
| 105.06988 | C8H9 | 1 |
| 112.08692 | C5H10N3 | 1 |
| 113.07094 | C5H9N2O | 1 |
| 115.05423 | C9H7 | 1 |
| 117.06988 | C9H9 | 1 |
| 127.08659 | C6H11N2O | 1 |
| 131.08553 | C10H11 | 1 |
| 133.10118 | C10H13 | 1 |
| 135.08044 | C9H11O | 1 |
| 140.08184 | C6H10N3O | 1 |
| 143.08553 | C11H11 | 1 |
| 152.07061 | C8H10NO2 | 1 |
| 155.08150 | C7H11N2O2 | 1 |
| 157.10839 | C6H13N4O | 1 |
| 158.09240 | C6H12N3O2 | 1 |
| 161.09609 | C11H13O | 1 |
| 163.11174 | C11H15O | 1 |
| 167.08150 | C8H11N2O2 | 1 |
| 169.09715 | C8H13N2O2 | 1 |
| 171.11683 | C13H15 | 1 |
| 183.08765 | C7H11N4O2 | 1 |
| 184.10805 | C8H14N3O2 | 1 |
| 195.07642 | C9H11N2O3 | 1 |
| 209.13248 | C16H17 | 1 |
| 200.11420 | C7H14N5O2 | 1 |
| 211.11895 | C9H15N4O2 | 1 |
| 212.10297 | C9H14N3O3 | 1 |
| 213.08698 | C9H13N2O4 | 1 |
| 228.14550 | C9H18N5O2 | 1 |
| 230.11353 | C9H16N3O4 | 1 |
| 237.09822 | C10H13N4O3 | 1 |
| 241.12952 | C10H17N4O3 | 1 |
| 255.10878 | C10H15N4O4 | 1 |
| 258.18524 | C17H24NO | 1 |
| 265.15869 | C19H21O | 1 |
| 272.13533 | C10H18N5O4 | 1 |
| 282.18524 | C19H24NO | 1 |
| 289.16188 | C10H21N6O4 | 1 |
| 311.18262 | C13H23N6O3 | 1 |
| 351.17753 | C15H23N6O4 | 1 |
| 426.20956 | C17H28N7O6 | 1 |
| 512.78236 | C48H75N13O12 | 2 |
| 582.31067 | C23H40N11O7 | 1 |
| 682.39227 | C35H52N7O7 | 1 |
| 753.42939 | C38H57N8O8 | 1 |
| 770.45594 | C38H60N9O8 | 1 |
| 909.53050 | C44H69N12O9 | 1 |
| 946.51049 | C42H68N13O12 | 1 |
| 1006.54688 | C48H72N13O11 | 1 |
| 1024.55744 | C48H74N13O12 | 1 |
| **Cyanopeptolin 1041** | | |
| 72.08078 | C4H10N | 1 |
| 74.06004 | C3H8NO | 1 |
| 88.03930 | C3H6NO2 | 1 |
| 184.05237 | C9H11ClNO | 1 |
| 197.10464 | C10H15NO3 | 1 |
| 212.04728 | C10H11ClNO2 | 1 |
| 215.11521 | C10H17NO4 | 1 |
| 223.11895 | C10H15N4O2 | 1 |
| 240.14550 | C10H18N5O2 | 1 |
| 342.08915 | C19H17ClNO3 | 1 |
| 453.24561 | C20H33N6O6 | 1 |
| 828.38058 | C39H55ClN9O9 | 1 |
| 1041.48069 | C49H70ClN10O13 | 1 |
| **Ferintoic Acid A** | | |
| 72.08078 | C4H10N | 1 |
| 84.08078 | C5H10N | 1 |
| 86.06004 | C4H8NO | 1 |
| 107.04914 | C7H7O | 1 |
| 114.05496 | C5H8NO2 | 1 |
| 120.08078 | C8H10N | 1 |
| 129.10224 | C6H13N2O | 1 |
| 130.06513 | C9H8N | 1 |
| 132.08078 | C9H10N | 1 |
| 150.09134 | C9H12NO | 1 |
| 159.09167 | C10H11N2 | 1 |
| 185.07094 | C11H9N2O | 1 |
| 188.07061 | C11H10NO2 | 1 |
| 205.09715 | C11H13N2O2 | 1 |
| 205.13354 | C12H17N2O | 1 |
| 233.12845 | C13H17N2O2 | 1 |
| 249.15975 | C14H21N2O2 | 1 |
| 254.14992 | C12H20N3O3 | 1 |
| 261.13594 | C15H19NO3 | 1 |
| 263.13902 | C14H19N2O3 | 1 |
| 277.15467 | C15H21N2O3 | 1 |
| 341.16082 | C18H21N4O3 | 1 |
| 362.20743 | C19H28N3O4 | 1 |
| 379.23398 | C19H31N4O4 | 1 |
| 412.23432 | C22H30N5O3 | 1 |
| 478.24488 | C26H32N5O4 | 1 |
| 563.29764 | C30H39N6O5 | 1 |
| 591.29256 | C31H39N6O6 | 1 |
| 635.35516 | C34H47N6O6 | 1 |
| 636.36298 | C34H48N6O6 | 1 |
| 637.37081 | C34H49N6O6 | 1 |
| 663.35007 | C35H47N6O7 | 1 |
| 690.36097 | C36H48N7O7 | 1 |
| 691.36880 | C36H49N7O7 | 1 |
| 849.42939 | C46H57N8O8 | 1 |
| 867.43995 | C46H59N8O9 | 1 |
| **Microcystin-HilR** | | |
| 65.03858 | C5H5 | 1 |
| 77.03858 | C6H5 | 1 |
| 79.05423 | C6H7 | 1 |
| 84.04439 | C4H6NO | 1 |
| 91.05423 | C7H7 | 1 |
| 98.06004 | C5H8NO | 1 |
| 100.11208 | C6H14N | 1 |
| 103.05423 | C8H7 | 1 |
| 105.06988 | C8H9 | 1 |
| 112.08692 | C5H10N3 | 1 |
| 113.07094 | C5H9N2O | 1 |
| 115.05423 | C9H7 | 1 |
| 117.06988 | C9H9 | 1 |
| 127.08659 | C6H11N2O | 1 |
| 130.04987 | C5H8NO3 | 1 |
| 131.08553 | C10H11 | 1 |
| 133.10118 | C10H13 | 1 |
| 135.08044 | C9H11O | 1 |
| 140.08184 | C6H10N3O | 1 |
| 141.06585 | C6H9N2O2 | 1 |
| 145.10118 | C11H13 | 1 |
| 155.08150 | C7H11N2O2 | 1 |
| 157.10839 | C6H13N4O | 1 |
| 161.09609 | C11H13O | 1 |
| 163.11174 | C11H15O | 1 |
| 171.11683 | C13H15 | 1 |
| 183.08765 | C7H11N4O2 | 1 |
| 195.07642 | C9H11N2O3 | 1 |
| 200.11420 | C7H14N5O2 | 1 |
| 209.13248 | C16H17 | 1 |
| 213.08698 | C9H13N2O4 | 1 |
| 226.11862 | C10H16N3O3 | 1 |
| 241.12952 | C10H17N4O3 | 1 |
| 244.12918 | C10H18N3O4 | 1 |
| 258.15607 | C10H20N5O3 | 1 |
| 258.18524 | C17H24NO | 1 |
| 265.15869 | C19H21O | 1 |
| 268.14042 | C11H18N5O3 | 1 |
| 269.12443 | C11H17N4O4 | 1 |
| 286.15098 | C11H20N5O4 | 1 |
| 375.19200 | C20H27N2O5 | 1 |
| 411.22381 | C19H31N4O6 | 1 |
| 539.33001 | C24H43N8O6 | 1 |
| 550.30240 | C30H40N5O5 | 1 |
| 567.32492 | C25H43N8O7 | 1 |
| 599.35516 | C31H47N6O6 | 1 |
| 852.53419 | C44H70N9O8 | 1 |
| 875.49853 | C41H67N10O11 | 1 |
| 925.47779 | C44H65N10O12 | 1 |
| 937.58695 | C48H77N10O9 | 1 |
| 964.55023 | C49H74N9O11 | 1 |
| 981.57678 | C49H77N10O11 | 1 |
| 992.54515 | C50H74N9O12 | 1 |
| 1009.57169 | C50H77N10O12 | 1 |
| **Microcystin-HtyR** | | |
| 77.03858 | C6H5 | 1 |
| 79.05423 | C6H7 | 1 |
| 84.04439 | C4H6NO | 1 |
| 91.05423 | C7H7 | 1 |
| 98.06004 | C5H8NO | 1 |
| 102.05496 | C4H8NO2 | 1 |
| 103.05423 | C8H7 | 1 |
| 105.06988 | C8H9 | 1 |
| 107.04914 | C7H7O | 1 |
| 112.08692 | C5H10N3 | 1 |
| 113.07094 | C5H9N2O | 1 |
| 115.05423 | C9H7 | 1 |
| 117.06988 | C9H9 | 1 |
| 119.04914 | C8H7O | 1 |
| 127.08659 | C6H11N2O | 1 |
| 129.11347 | C5H13N4 | 1 |
| 130.04987 | C5H8NO3 | 1 |
| 131.08553 | C10H11 | 1 |
| 133.06479 | C9H9O | 1 |
| 135.08044 | C9H11O | 1 |
| 140.08184 | C6H10N3O | 1 |
| 141.06585 | C6H9N2O2 | 1 |
| 143.08553 | C11H11 | 1 |
| 145.10118 | C11H13 | 1 |
| 150.09134 | C9H12NO | 1 |
| 155.08150 | C7H11N2O2 | 1 |
| 157.10839 | C6H13N4O | 1 |
| 161.09609 | C11H13O | 1 |
| 163.11174 | C11H15O | 1 |
| 171.11683 | C13H15 | 1 |
| 183.08765 | C7H11N4O2 | 1 |
| 195.07642 | C9H11N2O3 | 1 |
| 200.11420 | C7H14N5O2 | 1 |
| 209.13248 | C16H17 | 1 |
| 211.07133 | C9H11N2O4 | 1 |
| 213.08698 | C9H13N2O4 | 1 |
| 226.11862 | C10H16N3O3 | 1 |
| 241.12952 | C10H17N4O3 | 1 |
| 249.13460 | C12H17N4O2 | 1 |
| 258.15607 | C10H20N5O3 | 1 |
| 258.18524 | C17H24NO | 1 |
| 265.15869 | C19H21O | 1 |
| 268.14042 | C11H18N5O3 | 1 |
| 269.12443 | C11H17N4O4 | 1 |
| 286.15098 | C11H20N5O4 | 1 |
| 307.12885 | C15H19N2O5 | 1 |
| 375.19200 | C20H27N2O5 | 1 |
| 446.20341 | C21H28N5O6 | 1 |
| 461.20308 | C22H29N4O7 | 1 |
| 463.22996 | C21H31N6O6 | 1 |
| 571.36025 | C30H47N6O5 | 1 |
| 589.30927 | C27H41N8O7 | 1 |
| 925.47779 | C44H65N10O12 | 1 |
| 1041.54039 | C53H73N10O12 | 1 |
| 1059.55096 | C53H75N10O13 | 1 |
| **Microcystin-LA** | | |
| 69.03349 | C4H5O | 1 |
| 77.03858 | C6H5 | 1 |
| 79.05423 | C6H7 | 1 |
| 84.04439 | C4H6NO | 1 |
| 86.09643 | C5H12N | 1 |
| 91.05423 | C7H7 | 1 |
| 102.05496 | C4H8NO2 | 1 |
| 103.05423 | C8H7 | 1 |
| 105.06988 | C8H9 | 1 |
| 115.05423 | C9H7 | 1 |
| 117.06988 | C9H9 | 1 |
| 119.08553 | C9H11 | 1 |
| 127.08659 | C6H11N2O | 1 |
| 129.06988 | C10H9 | 1 |
| 130.04987 | C5H8NO3 | 1 |
| 131.08553 | C10H11 | 1 |
| 133.10118 | C10H13 | 1 |
| 135.08044 | C9H11O | 1 |
| 141.06585 | C6H9N2O2 | 1 |
| 143.08553 | C11H11 | 1 |
| 145.10118 | C11H13 | 1 |
| 155.08150 | C7H11N2O2 | 1 |
| 156.06552 | C7H10NO3 | 1 |
| 159.11683 | C12H15 | 1 |
| 161.09609 | C11H13O | 1 |
| 163.11174 | C11H15O | 1 |
| 168.06552 | C8H10NO3 | 1 |
| 169.10118 | C13H13 | 1 |
| 171.11683 | C13H15 | 1 |
| 172.10805 | C7H14N3O2 | 1 |
| 173.09207 | C7H13N2O3 | 1 |
| 183.07642 | C8H11N2O3 | 1 |
| 185.12845 | C9H17N2O2 | 1 |
| 187.11174 | C13H15O | 1 |
| 195.07642 | C9H11N2O3 | 1 |
| 196.06043 | C9H10NO4 | 1 |
| 200.10297 | C8H14N3O3 | 1 |
| 201.08698 | C8H13N2O4 | 1 |
| 209.13248 | C16H17 | 1 |
| 213.08698 | C9H13N2O4 | 1 |
| 226.11862 | C10H16N3O3 | 1 |
| 243.13393 | C11H19N2O4 | 1 |
| 258.18524 | C17H24NO | 1 |
| 265.15869 | C19H21O | 1 |
| 266.11353 | C12H16N3O4 | 1 |
| 268.16557 | C13H22N3O3 | 1 |
| 269.11320 | C12H17N2O5 | 1 |
| 282.18524 | C19H24NO | 1 |
| 297.18491 | C20H25O2 | 1 |
| 314.17105 | C14H24N3O5 | 1 |
| 375.19200 | C20H27N2O5 | 1 |
| 397.20816 | C18H29N4O6 | 1 |
| 440.25036 | C20H34N5O6 | 1 |
| 468.24527 | C21H34N5O7 | 1 |
| 508.24019 | C23H34N5O8 | 1 |
| 509.26461 | C29H37N2O6 | 1 |
| 548.27551 | C31H38N3O6 | 1 |
| 559.31263 | C29H43N4O7 | 1 |
| 580.30173 | C32H42N3O7 | 1 |
| 597.28787 | C26H41N6O10 | 1 |
| 597.28787 | C26H41N6O10 | 1 |
| 661.35958 | C37H49N4O7 | 1 |
| 680.32498 | C30H46N7O11 | 1 |
| 693.38579 | C38H53N4O8 | 1 |
| 725.42324 | C38H57N6O8 | 1 |
| 758.40832 | C37H56N7O10 | 1 |
| 758.40832 | C37H56N7O10 | 1 |
| 776.41888 | C37H58N7O11 | 1 |
| 892.48148 | C46H66N7O11 | 1 |
| 910.49205 | C46H68N7O12 | 1 |
| **Microcystin-LF** | | |
| 69.03349 | C4H5O | 1 |
| 77.03858 | C6H5 | 1 |
| 79.05423 | C6H7 | 1 |
| 84.04439 | C4H6NO | 1 |
| 86.09643 | C5H12N | 1 |
| 91.05423 | C7H7 | 1 |
| 102.05496 | C4H8NO2 | 1 |
| 103.05423 | C8H7 | 1 |
| 105.06988 | C8H9 | 1 |
| 115.05423 | C9H7 | 1 |
| 117.06988 | C9H9 | 1 |
| 119.08553 | C9H11 | 1 |
| 120.08078 | C8H10N | 1 |
| 121.06479 | C8H9O | 1 |
| 123.08044 | C8H11O | 1 |
| 127.08659 | C6H11N2O | 1 |
| 129.06988 | C10H9 | 1 |
| 130.04987 | C5H8NO3 | 1 |
| 131.08553 | C10H11 | 1 |
| 133.10118 | C10H13 | 1 |
| 135.08044 | C9H11O | 1 |
| 143.08553 | C11H11 | 1 |
| 145.10118 | C11H13 | 1 |
| 155.08150 | C7H11N2O2 | 1 |
| 159.11683 | C12H15 | 1 |
| 161.09609 | C11H13O | 1 |
| 163.11174 | C11H15O | 1 |
| 165.10224 | C9H13N2O | 1 |
| 169.10118 | C13H13 | 1 |
| 171.11683 | C13H15 | 1 |
| 172.10805 | C7H14N3O2 | 1 |
| 173.09207 | C7H13N2O3 | 1 |
| 175.11174 | C12H15O | 1 |
| 183.07642 | C8H11N2O3 | 1 |
| 185.12845 | C9H17N2O2 | 1 |
| 187.11174 | C13H15O | 1 |
| 195.07642 | C9H11N2O3 | 1 |
| 196.06043 | C9H10NO4 | 1 |
| 200.10297 | C8H14N3O3 | 1 |
| 201.08698 | C8H13N2O4 | 1 |
| 209.13248 | C16H17 | 1 |
| 213.08698 | C9H13N2O4 | 1 |
| 232.09682 | C13H14NO3 | 1 |
| 243.13393 | C11H19N2O4 | 1 |
| 249.12337 | C13H17N2O3 | 1 |
| 251.10263 | C12H15N2O4 | 1 |
| 258.18524 | C17H24NO | 1 |
| 259.10772 | C14H15N2O3 | 1 |
| 265.15869 | C19H21O | 1 |
| 266.11353 | C12H16N3O4 | 1 |
| 268.16557 | C13H22N3O3 | 1 |
| 269.11320 | C12H17N2O5 | 1 |
| 277.11828 | C14H17N2O4 | 1 |
| 282.18524 | C19H24NO | 1 |
| 294.14483 | C14H20N3O4 | 1 |
| 297.18491 | C20H25O2 | 1 |
| 314.21146 | C20H28NO2 | 1 |
| 357.18088 | C20H25N2O4 | 1 |
| 362.20743 | C19H28N3O4 | 1 |
| 375.19200 | C20H27N2O5 | 1 |
| 390.20235 | C20H28N3O5 | 1 |
| 397.20816 | C18H29N4O6 | 1 |
| 426.22750 | C25H32NO5 | 1 |
| 461.23946 | C23H33N4O6 | 1 |
| 509.26461 | C29H37N2O6 | 1 |
| 516.28166 | C26H38N5O6 | 1 |
| 544.27657 | C27H38N5O7 | 1 |
| 548.27551 | C31H38N3O6 | 1 |
| 559.31263 | C29H43N4O7 | 1 |
| 580.30173 | C32H42N3O7 | 1 |
| 655.30860 | C32H43N6O9 | 1 |
| 661.35958 | C37H49N4O7 | 1 |
| 673.31917 | C32H45N6O10 | 1 |
| 693.38579 | C38H53N4O8 | 1 |
| 801.45454 | C44H61N6O8 | 1 |
| 807.42872 | C42H59N6O10 | 1 |
| 822.42838 | C43H60N5O11 | 1 |
| 834.43962 | C43H60N7O10 | 1 |
| 852.45018 | C43H62N7O11 | 1 |
| 936.48657 | C51H66N7O10 | 1 |
| 940.51787 | C51H70N7O10 | 1 |
| 955.50496 | C51H69N7O11 | 1 |
| 968.51278 | C52H70N7O11 | 1 |
| 986.52335 | C52H72N7O12 | 1 |
| **Microcystin-LR** | | |
| 77.03858 | C6H5 | 1 |
| 79.05423 | C6H7 | 1 |
| 84.04439 | C4H6NO | 1 |
| 86.09643 | C5H12N | 1 |
| 87.07910 | C3H9N3 | 1 |
| 91.05423 | C7H7 | 1 |
| 98.06004 | C5H8NO | 1 |
| 102.05496 | C4H8NO2 | 1 |
| 103.05423 | C8H7 | 1 |
| 105.06988 | C8H9 | 1 |
| 112.08692 | C5H10N3 | 1 |
| 113.07094 | C5H9N2O | 1 |
| 115.05423 | C9H7 | 1 |
| 117.06988 | C9H9 | 1 |
| 127.08659 | C6H11N2O | 1 |
| 130.04987 | C5H8NO3 | 1 |
| 131.08553 | C10H11 | 1 |
| 133.10118 | C10H13 | 1 |
| 135.08044 | C9H11O | 1 |
| 140.08184 | C6H10N3O | 1 |
| 141.06585 | C6H9N2O2 | 1 |
| 147.07642 | C5H11N2O3 | 1 |
| 155.08150 | C7H11N2O2 | 1 |
| 157.10839 | C6H13N4O | 1 |
| 161.09609 | C11H13O | 1 |
| 163.11174 | C11H15O | 1 |
| 183.08765 | C7H11N4O2 | 1 |
| 195.07642 | C9H11N2O3 | 1 |
| 200.11420 | C7H14N5O2 | 1 |
| 213.08698 | C9H13N2O4 | 1 |
| 226.11862 | C10H16N3O3 | 1 |
| 241.12952 | C10H17N4O3 | 1 |
| 258.18524 | C17H24NO | 1 |
| 268.14042 | C11H18N5O3 | 1 |
| 268.16557 | C13H22N3O3 | 1 |
| 269.12443 | C11H17N4O4 | 1 |
| 282.18524 | C19H24NO | 1 |
| 286.15098 | C11H20N5O4 | 1 |
| 375.19200 | C20H27N2O5 | 1 |
| 382.20850 | C17H28N5O5 | 1 |
| 397.20816 | C18H29N4O6 | 1 |
| 399.23504 | C17H31N6O5 | 1 |
| 525.31436 | C23H41N8O6 | 1 |
| 553.30927 | C24H41N8O7 | 1 |
| 571.36025 | C30H47N6O5 | 1 |
| 950.53458 | C48H72N9O11 | 1 |
| 967.56113 | C48H75N10O11 | 1 |
| 995.55604 | C49H75N10O12 | 1 |
| **Microcystin-LW** | | |
| 77.03858 | C6H5 | 1 |
| 79.05423 | C6H7 | 1 |
| 84.04439 | C4H6NO | 1 |
| 86.09643 | C5H12N | 1 |
| 91.05423 | C7H7 | 1 |
| 102.05496 | C4H8NO2 | 1 |
| 103.05423 | C8H7 | 1 |
| 105.06988 | C8H9 | 1 |
| 115.05423 | C9H7 | 1 |
| 117.06988 | C9H9 | 1 |
| 119.08553 | C9H11 | 1 |
| 129.06988 | C10H9 | 1 |
| 130.04987 | C5H8NO3 | 1 |
| 130.06513 | C9H8N | 1 |
| 131.08553 | C10H11 | 1 |
| 133.10118 | C10H13 | 1 |
| 135.08044 | C9H11O | 1 |
| 143.08553 | C11H11 | 1 |
| 145.10118 | C11H13 | 1 |
| 147.07642 | C5H11N2O3 | 1 |
| 155.08150 | C7H11N2O2 | 1 |
| 159.11683 | C12H15 | 1 |
| 161.09609 | C11H13O | 1 |
| 163.11174 | C11H15O | 1 |
| 170.08117 | C8H12NO3 | 1 |
| 171.11683 | C13H15 | 1 |
| 175.11174 | C12H15O | 1 |
| 185.12845 | C9H17N2O2 | 1 |
| 187.08659 | C11H11N2O | 1 |
| 187.11174 | C13H15O | 1 |
| 195.07642 | C9H11N2O3 | 1 |
| 196.06043 | C9H10NO4 | 1 |
| 209.13248 | C16H17 | 1 |
| 213.08698 | C9H13N2O4 | 1 |
| 215.08150 | C12H11N2O2 | 1 |
| 241.12952 | C10H17N4O3 | 1 |
| 243.1128 | C14H15N2O2 | 1 |
| 243.13393 | C11H19N2O4 | 1 |
| 251.10263 | C12H15N2O4 | 1 |
| 258.18524 | C17H24NO | 1 |
| 269.11320 | C12H17N2O5 | 1 |
| 282.18524 | C19H24NO | 1 |
| 288.13427 | C15H18N3O3 | 1 |
| 297.18491 | C20H25O2 | 1 |
| 298.11862 | C16H16N3O3 | 1 |
| 299.10263 | C16H15N2O4 | 1 |
| 316.12918 | C16H18N3O4 | 1 |
| 375.19200 | C20H27N2O5 | 1 |
| 397.20816 | C18H29N4O6 | 1 |
| 411.20268 | C22H27N4O4 | 1 |
| 426.22750 | C25H32NO5 | 1 |
| 429.21325 | C22H29N4O5 | 1 |
| 500.25036 | C25H34N5O6 | 1 |
| 508.24019 | C23H34N5O8 | 1 |
| 580.30173 | C32H42N3O7 | 1 |
| 582.30535 | C31H42N4O7 | 0 |
| 795.36718 | C38H51N8O11 | 1 |
| 840.46544 | C46H62N7O8 | 1 |
| 847.43487 | C43H59N8O10 | 1 |
| 873.45052 | C45H61N8O10 | 1 |
| 891.46108 | C45H63N8O11 | 1 |
| 980.51278 | C53H70N7O11 | 1 |
| 1007.56007 | C55H75N8O10 | 1 |
| 1025.53425 | C54H73N8O12 | 1 |
| **Microcystin-LY** | | |
| 77.03858 | C6H5 | 1 |
| 79.05423 | C6H7 | 1 |
| 84.04439 | C4H6NO | 1 |
| 86.09643 | C5H12N | 1 |
| 91.05423 | C7H7 | 1 |
| 102.05496 | C4H8NO2 | 1 |
| 103.05423 | C8H7 | 1 |
| 105.06988 | C8H9 | 1 |
| 115.05423 | C9H7 | 1 |
| 117.06988 | C9H9 | 1 |
| 119.04914 | C8H7O | 1 |
| 119.08553 | C9H11 | 1 |
| 121.06479 | C8H9O | 1 |
| 127.08659 | C6H11N2O | 1 |
| 129.06988 | C10H9 | 1 |
| 130.04987 | C5H8NO3 | 1 |
| 131.08553 | C10H11 | 1 |
| 133.10118 | C10H13 | 1 |
| 135.08044 | C9H11O | 1 |
| 136.07569 | C8H10NO | 1 |
| 143.08553 | C11H11 | 1 |
| 145.10118 | C11H13 | 1 |
| 155.08150 | C7H11N2O2 | 1 |
| 159.11683 | C12H15 | 1 |
| 161.09609 | C11H13O | 1 |
| 163.11174 | C11H15O | 1 |
| 168.06552 | C8H10NO3 | 1 |
| 169.10118 | C13H13 | 1 |
| 171.11683 | C13H15 | 1 |
| 173.09207 | C7H13N2O3 | 1 |
| 183.07642 | C8H11N2O3 | 1 |
| 185.12845 | C9H17N2O2 | 1 |
| 187.11174 | C13H15O | 1 |
| 195.07642 | C9H11N2O3 | 1 |
| 196.06043 | C9H10NO4 | 1 |
| 209.13248 | C16H17 | 1 |
| 213.08698 | C9H13N2O4 | 1 |
| 243.13393 | C11H19N2O4 | 1 |
| 248.09173 | C13H14NO4 | 1 |
| 258.18524 | C17H24NO | 1 |
| 265.11828 | C13H17N2O4 | 1 |
| 265.15869 | C19H21O | 1 |
| 266.11353 | C12H16N3O4 | 1 |
| 268.16557 | C13H22N3O3 | 1 |
| 269.11320 | C12H17N2O5 | 1 |
| 282.18524 | C19H24NO | 1 |
| 293.11320 | C14H17N2O5 | 1 |
| 297.18491 | C20H25O2 | 1 |
| 310.10336 | C13H16N3O6 | 1 |
| 310.13975 | C14H20N3O5 | 1 |
| 314.21146 | C20H28NO2 | 1 |
| 375.19200 | C20H27N2O5 | 1 |
| 378.20235 | C19H28N3O5 | 1 |
| 397.20816 | C18H29N4O6 | 1 |
| 406.19726 | C20H28N3O6 | 1 |
| 477.23438 | C23H33N4O7 | 1 |
| 494.26092 | C23H36N5O7 | 1 |
| 509.26461 | C29H37N2O6 | 1 |
| 548.27551 | C31H38N3O6 | 1 |
| 559.31263 | C29H43N4O7 | 1 |
| 580.30173 | C32H42N3O7 | 1 |
| 661.35958 | C37H49N4O7 | 1 |
| 689.31408 | C32H45N6O11 | 1 |
| 693.38579 | C38H53N4O8 | 1 |
| 817.44945 | C44H61N6O9 | 1 |
| 823.42363 | C42H59N6O11 | 1 |
| 850.43453 | C43H60N7O11 | 1 |
| 868.44510 | C43H62N7O12 | 1 |
| 984.50770 | C52H70N7O12 | 1 |
| 986.48696 | C51H68N7O13 | 1 |
| 1002.51826 | C52H72N7O13 | 1 |
| **Microcystin-RR** | | |
| 77.03858 | C6H5 | 1 |
| 79.05423 | C6H7 | 1 |
| 84.04439 | C4H6NO | 1 |
| 86.09643 | C5H12N | 1 |
| 91.05423 | C7H7 | 1 |
| 102.05496 | C4H8NO2 | 1 |
| 103.05423 | C8H7 | 1 |
| 105.06988 | C8H9 | 1 |
| 112.08692 | C5H10N3 | 1 |
| 113.07094 | C5H9N2O | 1 |
| 115.05423 | C9H7 | 1 |
| 117.06988 | C9H9 | 1 |
| 119.08553 | C9H11 | 1 |
| 127.08659 | C6H11N2O | 1 |
| 130.04987 | C5H8NO3 | 1 |
| 131.08553 | C10H11 | 1 |
| 133.10118 | C10H13 | 1 |
| 135.08044 | C9H11O | 1 |
| 140.08184 | C6H10N3O | 1 |
| 141.06585 | C6H9N2O2 | 1 |
| 143.08553 | C11H11 | 1 |
| 155.08150 | C7H11N2O2 | 1 |
| 157.10839 | C6H13N4O | 1 |
| 161.09609 | C11H13O | 1 |
| 163.11174 | C11H15O | 1 |
| 169.09715 | C8H13N2O2 | 1 |
| 183.08765 | C7H11N4O2 | 1 |
| 195.07642 | C9H11N2O3 | 1 |
| 200.11420 | C7H14N5O2 | 1 |
| 209.09207 | C10H13N2O3 | 1 |
| 211.11895 | C9H15N4O2 | 1 |
| 213.08698 | C9H13N2O4 | 1 |
| 226.11862 | C10H16N3O3 | 1 |
| 228.14550 | C9H18N5O2 | 1 |
| 241.12952 | C10H17N4O3 | 1 |
| 258.18524 | C17H24NO | 1 |
| 265.15869 | C19H21O | 1 |
| 268.14042 | C11H18N5O3 | 1 |
| 269.12443 | C11H17N4O4 | 1 |
| 282.18524 | C19H24NO | 1 |
| 286.15098 | C11H20N5O4 | 1 |
| 303.17753 | C11H23N6O4 | 1 |
| 311.18262 | C13H23N6O3 | 1 |
| 357.18809 | C14H25N6O5 | 1 |
| 440.22521 | C18H30N7O6 | 1 |
| 519.79018 | C49H77N13O12 | 2 |
| 571.36025 | C30H47N6O5 | 1 |
| 596.32632 | C24H42N11O7 | 1 |
| 886.48936 | C40H64N13O10 | 1 |
| 888.46863 | C39H62N13O11 | 1 |
| 904.49993 | C40H66N13O11 | 1 |
| 978.54073 | C48H72N11O11 | 1 |
| 993.55163 | C48H73N12O11 | 1 |
| 996.55129 | C48H74N11O12 | 1 |
| 1020.56253 | C49H74N13O11 | 1 |
| 1038.57309 | C49H76N13O12 | 1 |
| **Microcystin-WR** | | |
| 77.03858 | C6H5 | 1 |
| 79.05423 | C6H7 | 1 |
| 84.04439 | C4H6NO | 1 |
| 91.05423 | C7H7 | 1 |
| 98.06004 | C5H8NO | 1 |
| 102.05496 | C4H8NO2 | 1 |
| 103.05423 | C8H7 | 1 |
| 105.06988 | C8H9 | 1 |
| 112.03930 | C5H6NO2 | 1 |
| 112.08692 | C5H10N3 | 1 |
| 113.07094 | C5H9N2O | 1 |
| 117.05785 | C8H7N | 0 |
| 117.06988 | C9H9 | 1 |
| 119.08553 | C9H11 | 1 |
| 127.08659 | C6H11N2O | 1 |
| 129.11347 | C5H13N4 | 1 |
| 130.04987 | C5H8NO3 | 1 |
| 130.06513 | C9H8N | 1 |
| 131.08553 | C10H11 | 1 |
| 135.08044 | C9H11O | 1 |
| 140.08184 | C6H10N3O | 1 |
| 141.06585 | C6H9N2O2 | 1 |
| 143.08553 | C11H11 | 1 |
| 145.10118 | C11H13 | 1 |
| 155.08150 | C7H11N2O2 | 1 |
| 157.10839 | C6H13N4O | 1 |
| 159.09167 | C10H11N2 | 1 |
| 161.09609 | C11H13O | 1 |
| 163.11174 | C11H15O | 1 |
| 170.06004 | C11H8NO | 1 |
| 171.11683 | C13H15 | 1 |
| 183.08765 | C7H11N4O2 | 1 |
| 187.08659 | C11H11N2O | 1 |
| 195.07642 | C9H11N2O3 | 1 |
| 196.06043 | C9H10NO4 | 1 |
| 200.11420 | C7H14N5O2 | 1 |
| 209.13248 | C16H17 | 1 |
| 213.08698 | C9H13N2O4 | 1 |
| 226.11862 | C10H16N3O3 | 1 |
| 241.12952 | C10H17N4O3 | 1 |
| 251.11387 | C11H15N4O3 | 1 |
| 255.10878 | C10H15N4O4 | 1 |
| 258.15607 | C10H20N5O3 | 1 |
| 258.18524 | C17H24NO | 1 |
| 265.15869 | C19H21O | 1 |
| 268.14042 | C11H18N5O3 | 1 |
| 269.12443 | C11H17N4O4 | 1 |
| 282.18524 | C19H24NO | 1 |
| 286.15098 | C11H20N5O4 | 1 |
| 298.11862 | C16H16N3O3 | 1 |
| 299.10263 | C16H15N2O4 | 1 |
| 341.16082 | C18H21N4O3 | 1 |
| 395.17138 | C21H23N4O4 | 1 |
| 454.21973 | C22H28N7O4 | 1 |
| 455.20374 | C22H27N6O5 | 1 |
| 470.20341 | C23H28N5O6 | 1 |
| 472.23029 | C22H30N7O5 | 1 |
| 543.26741 | C25H35N8O6 | 1 |
| 571.36025 | C30H47N6O5 | 1 |
| 598.30961 | C28H40N9O6 | 1 |
| 599.35516 | C31H47N6O6 | 1 |
| 625.32319 | C33H45N4O8 | 1 |
| 626.30452 | C29H40N9O7 | 1 |
| 682.39227 | C35H52N7O7 | 1 |
| 1023.52983 | C53H71N10O11 | 1 |
| 1040.55638 | C53H74N11O11 | 1 |
| 1068.55129 | C54H74N11O12 | 1 |
| **Microcystin-YR** | | |
| 77.03858 | C6H5 | 1 |
| 79.05423 | C6H7 | 1 |
| 84.04439 | C4H6NO | 1 |
| 86.09643 | C5H12N | 1 |
| 87.07910 | C3H9N3 | 1 |
| 91.05423 | C7H7 | 1 |
| 95.04914 | C6H7O | 1 |
| 98.06004 | C5H8NO | 1 |
| 103.05423 | C8H7 | 1 |
| 105.06988 | C8H9 | 1 |
| 112.08692 | C5H10N3 | 1 |
| 113.07094 | C5H9N2O | 1 |
| 115.05423 | C9H7 | 1 |
| 117.06988 | C9H9 | 1 |
| 119.04914 | C8H7O | 1 |
| 127.08659 | C6H11N2O | 1 |
| 130.04987 | C5H8NO3 | 1 |
| 131.08553 | C10H11 | 1 |
| 133.10118 | C10H13 | 1 |
| 135.08044 | C9H11O | 1 |
| 136.07569 | C8H10NO | 1 |
| 140.08184 | C6H10N3O | 1 |
| 141.06585 | C6H9N2O2 | 1 |
| 143.08553 | C11H11 | 1 |
| 145.10118 | C11H13 | 1 |
| 147.04406 | C9H7O2 | 1 |
| 155.08150 | C7H11N2O2 | 1 |
| 157.10839 | C6H13N4O | 1 |
| 161.09609 | C11H13O | 1 |
| 163.11174 | C11H15O | 1 |
| 183.08765 | C7H11N4O2 | 1 |
| 195.07642 | C9H11N2O3 | 1 |
| 196.06043 | C9H10NO4 | 1 |
| 200.11420 | C7H14N5O2 | 1 |
| 209.13248 | C16H17 | 1 |
| 213.08698 | C9H13N2O4 | 1 |
| 226.11862 | C10H16N3O3 | 1 |
| 241.12952 | C10H17N4O3 | 1 |
| 251.11387 | C11H15N4O3 | 1 |
| 258.15607 | C10H20N5O3 | 1 |
| 258.18524 | C17H24NO | 1 |
| 265.15869 | C19H21O | 1 |
| 268.14042 | C11H18N5O3 | 1 |
| 269.12443 | C11H17N4O4 | 1 |
| 282.18524 | C19H24NO | 1 |
| 286.15098 | C11H20N5O4 | 1 |
| 318.14483 | C16H20N3O4 | 1 |
| 375.19200 | C20H27N2O5 | 1 |
| 431.20374 | C20H27N6O5 | 1 |
| 432.18776 | C20H26N5O6 | 1 |
| 447.18743 | C21H27N4O7 | 1 |
| 449.21431 | C20H29N6O6 | 1 |
| 520.25142 | C23H34N7O7 | 1 |
| 571.36025 | C30H47N6O5 | 1 |
| 599.35516 | C31H47N6O6 | 1 |
| 603.28854 | C27H39N8O8 | 1 |
| 625.33442 | C32H45N6O7 | 1 |
| 911.46214 | C43H63N10O12 | 1 |
| 1045.53531 | C52H73N10O13 | 1 |
| **Microginin 527 Methyl Ester** | | |
| 55.05423 | C4H7 | 1 |
| 59.06037 | C2H7N2 | 1 |
| 69.06988 | C5H9 | 1 |
| 70.06513 | C4H8N | 1 |
| 88.03930 | C3H6NO2 | 1 |
| 91.05423 | C7H7 | 1 |
| 98.06004 | C5H8NO | 1 |
| 102.05496 | C4H8NO2 | 1 |
| 128.14338 | C8H18N | 1 |
| 130.08626 | C6H12NO2 | 1 |
| 136.07569 | C8H10NO | 1 |
| 140.14338 | C9H18N | 1 |
| 142.15903 | C9H20N | 1 |
| 148.07569 | C9H10NO | 1 |
| 154.12264 | C9H16NO | 1 |
| 158.15394 | C9H20NO | 1 |
| 168.13829 | C10H18NO | 1 |
| 180.06889 | C6H14NO3S | 1 |
| 195.08899 | C10H13NO3 | 1 |
| 319.12885 | C16H19N2O5 | 1 |
| 349.21555 | C16H33N2O4S | 1 |
| 357.14787 | C16H25N2O5S | 1 |
| 383.12713 | C17H23N2O6S | 1 |
| 415.15335 | C18H27N2O7S | 1 |
| 478.29116 | C25H40N3O6 | 1 |
| 510.26323 | C25H40N3O6S | 1 |
| 541.28217 | C26H43N3O7S | 1 |
| **Microginin 690 Methyl Ester** | | |
| 68.04948 | C4H6N | 1 |
| 69.06988 | C5H9 | 1 |
| 70.06513 | C4H8N | 1 |
| 88.03930 | C3H6NO2 | 1 |
| 88.07569 | C4H10NO | 1 |
| 98.06004 | C5H8NO | 1 |
| 99.05529 | C4H7N2O | 1 |
| 107.04914 | C7H7O | 1 |
| 116.07061 | C5H10NO2 | 1 |
| 128.14338 | C8H18N | 1 |
| 134.06004 | C8H8NO | 1 |
| 136.07569 | C8H10NO | 1 |
| 140.07061 | C7H10NO2 | 1 |
| 140.14338 | C9H18N | 1 |
| 142.15903 | C9H20N | 1 |
| 147.04406 | C9H7O2 | 1 |
| 148.07569 | C9H10NO | 1 |
| 149.02669 | C5H9O3S | 1 |
| 158.15394 | C9H20NO | 1 |
| 162.05833 | C6H12NO2S | 1 |
| 164.07398 | C6H14NO2S | 1 |
| 168.13829 | C10H18NO | 1 |
| 179.07027 | C10H11O3 | 1 |
| 180.06889 | C6H14NO3S | 1 |
| 190.05324 | C7H12NO3S | 1 |
| 194.08117 | C10H12NO3 | 1 |
| 196.09682 | C10H14NO3 | 1 |
| 205.09715 | C11H13N2O2 | 1 |
| 208.09682 | C11H14NO3 | 1 |
| 222.07608 | C11H12NO4 | 1 |
| 227.17540 | C12H23N2O2 | 1 |
| 255.20670 | C14H27N2O2 | 1 |
| 265.11828 | C13H17N2O4 | 1 |
| 279.13393 | C14H19N2O4 | 1 |
| 297.12674 | C14H21N2O3S | 1 |
| 319.20499 | C15H31N2O3S | 1 |
| 325.12165 | C15H21N2O4S | 1 |
| 358.15232 | C19H22N2O5 | 1 |
| 383.12713 | C17H23N2O6S | 1 |
| 418.27003 | C23H36N3O4 | 1 |
| 446.26495 | C24H36N3O5 | 1 |
| 482.26832 | C24H40N3O5S | 1 |
| 492.25267 | C25H38N3O5S | 1 |
| 510.26323 | C25H40N3O6S | 1 |
| 641.35449 | C34H49N4O8 | 1 |
| 705.35278 | C35H53N4O9S | 1 |
| **Microginin FR1** | | |
| 58.06513 | C3H8N | 1 |
| 59.06037 | C2H7N2 | 1 |
| 69.06988 | C5H9 | 1 |
| 100.11208 | C6H14N | 1 |
| 102.05496 | C4H8NO2 | 1 |
| 123.11683 | C9H15 | 1 |
| 128.10699 | C7H14NO | 1 |
| 128.10699 | C7H14NO | 1 |
| 128.14338 | C8H18N | 1 |
| 130.04987 | C5H8NO3 | 1 |
| 136.07569 | C8H10NO | 1 |
| 140.07061 | C7H10NO2 | 1 |
| 140.14338 | C9H18N | 1 |
| 142.15903 | C9H20N | 1 |
| 145.13354 | C7H17N2O | 1 |
| 154.12264 | C9H16NO | 1 |
| 156.10191 | C8H14NO2 | 1 |
| 158.15394 | C9H20NO | 1 |
| 168.13829 | C10H18NO | 1 |
| 171.14919 | C9H19N2O | 1 |
| 182.11756 | C10H16NO2 | 1 |
| 194.15394 | C12H20NO | 1 |
| 197.12845 | C10H17N2O2 | 1 |
| 199.14410 | C10H19N2O2 | 1 |
| 211.18049 | C12H23N2O | 1 |
| 227.13902 | C11H19N2O3 | 1 |
| 239.13902 | C12H19N2O3 | 1 |
| 239.17540 | C13H23N2O2 | 1 |
| 257.14958 | C12H21N2O4 | 1 |
| 257.18597 | C13H25N2O3 | 1 |
| 263.17540 | C15H23N2O2 | 1 |
| 291.17032 | C16H23N2O3 | 1 |
| 345.14450 | C18H21N2O5 | 1 |
| 366.27512 | C20H36N3O3 | 1 |
| 367.25913 | C20H35N2O4 | 1 |
| 384.28568 | C20H38N3O4 | 1 |
| 472.24421 | C25H34N3O6 | 1 |
| 547.34901 | C29H47N4O6 | 1 |
| 548.35684 | C29H48N4O6 | 1 |
| 728.42290 | C38H58N5O9 | 1 |
| **Nodularin-R** | | |
| 59.06037 | C2H7N2 | 1 |
| 60.05562 | CH6N3 | 1 |
| 69.03349 | C4H5O | 1 |
| 79.05423 | C6H7 | 1 |
| 84.04439 | C4H6NO | 1 |
| 91.05423 | C7H7 | 1 |
| 98.06004 | C5H8NO | 1 |
| 103.05423 | C8H7 | 1 |
| 105.06988 | C8H9 | 1 |
| 112.08692 | C5H10N3 | 1 |
| 115.05423 | C9H7 | 1 |
| 115.08659 | C5H11N2O | 1 |
| 117.06988 | C9H9 | 1 |
| 130.04987 | C5H8NO3 | 1 |
| 131.08553 | C10H11 | 1 |
| 133.10118 | C10H13 | 1 |
| 135.08044 | C9H11O | 1 |
| 140.08184 | C6H10N3O | 1 |
| 141.06585 | C6H9N2O2 | 1 |
| 143.08553 | C11H11 | 1 |
| 145.10118 | C11H13 | 1 |
| 157.10839 | C6H13N4O | 1 |
| 163.11174 | C11H15O | 1 |
| 171.11683 | C13H15 | 1 |
| 183.08765 | C7H11N4O2 | 1 |
| 199.10772 | C9H15N2O3 | 1 |
| 200.11420 | C7H14N5O2 | 1 |
| 209.09207 | C10H13N2O3 | 1 |
| 210.07608 | C10H12NO4 | 1 |
| 223.11895 | C10H15N4O2 | 1 |
| 225.08698 | C10H13N2O4 | 1 |
| 226.11862 | C10H16N3O3 | 1 |
| 227.10263 | C10H15N2O4 | 1 |
| 244.12918 | C10H18N3O4 | 1 |
| 253.08190 | C11H13N2O5 | 1 |
| 258.18524 | C17H24NO | 1 |
| 265.15869 | C19H21O | 1 |
| 268.14042 | C11H18N5O3 | 1 |
| 269.12443 | C11H17N4O4 | 1 |
| 282.18524 | C19H24NO | 1 |
| 286.15098 | C11H20N5O4 | 1 |
| 383.20374 | C16H27N6O5 | 1 |
| 466.24086 | C20H32N7O6 | 1 |
| 691.37735 | C32H51N8O9 | 1 |
| 781.46069 | C40H61N8O8 | 1 |
| 825.45052 | C41H61N8O10 | 1 |
| **Oscillamide Y** | | |
| 70.02874 | C3H4NO | 1 |
| 72.08078 | C4H10N | 1 |
| 84.04439 | C4H6NO | 1 |
| 84.08078 | C5H10N | 1 |
| 86.06004 | C4H8NO | 1 |
| 86.09643 | C5H12N | 1 |
| 107.04914 | C7H7O | 1 |
| 112.07569 | C6H10NO | 1 |
| 114.05496 | C5H8NO2 | 1 |
| 115.08659 | C5H11N2O | 1 |
| 120.08078 | C8H10N | 1 |
| 129.10224 | C6H13N2O | 1 |
| 133.06479 | C9H9O | 1 |
| 136.07569 | C8H10NO | 1 |
| 146.06004 | C9H8NO | 1 |
| 148.07569 | C9H10NO | 1 |
| 150.09134 | C9H12NO | 1 |
| 155.08150 | C7H11N2O2 | 1 |
| 165.05462 | C9H9O3 | 1 |
| 182.08117 | C9H12NO3 | 1 |
| 188.07061 | C11H10NO2 | 1 |
| 190.08626 | C11H12NO2 | 1 |
| 205.13354 | C12H17N2O | 1 |
| 233.12845 | C13H17N2O2 | 1 |
| 263.13902 | C14H19N2O3 | 1 |
| 263.17540 | C15H23N2O2 | 1 |
| 268.16557 | C13H22N3O3 | 1 |
| 291.17032 | C16H23N2O3 | 1 |
| 318.14483 | C16H20N3O4 | 1 |
| 320.16048 | C16H22N3O4 | 1 |
| 336.15540 | C16H22N3O5 | 1 |
| 376.22308 | C20H30N3O4 | 1 |
| 412.22308 | C23H30N3O4 | 1 |
| 455.22890 | C24H31N4O5 | 1 |
| 483.22381 | C25H31N4O6 | 1 |
| 540.28166 | C28H38N5O6 | 1 |
| 568.27657 | C29H38N5O7 | 1 |
| 649.37081 | C35H49N6O6 | 1 |
| 651.38646 | C35H51N6O6 | 1 |
| 663.35007 | C35H47N6O7 | 1 |
| 677.36572 | C36H49N6O7 | 1 |
| 681.36064 | C35H49N6O8 | 1 |
| 682.35589 | C34H48N7O8 | 1 |
| 830.44470 | C44H60N7O9 | 1 |
| 858.43962 | C45H60N7O10 | 1 |
